# Views on Using Social Robots in Professional Caregiving: Content Analysis of a Scenario Method Workshop

**DOI:** 10.2196/20046

**Published:** 2021-11-10

**Authors:** Theresa Sophie Busse, Sven Kernebeck, Larissa Nef, Patrick Rebacz, Ilona Kickbusch, Jan Peter Ehlers

**Affiliations:** 1 Department of Didactics and Educational Research in Healthcare Faculty of Health Witten/Herdecke University Witten Germany; 2 Careum Foundation Zurich Switzerland; 3 Visionom Witten Germany; 4 Department and Institute for Anatomy and Clinical Morphology Faculty of Health Witten/Herdecke University Witten Germany

**Keywords:** social robots, robotics, health care sector, health personnel, ethics, forecasting, trends, technology, digital transformation, professional caregiving

## Abstract

**Background:**

Interest in digital technologies in the health care sector is growing and can be a way to reduce the burden on professional caregivers while helping people to become more independent. Social robots are regarded as a special form of technology that can be usefully applied in professional caregiving with the potential to focus on interpersonal contact. While implementation is progressing slowly, a debate on the concepts and applications of social robots in future care is necessary.

**Objective:**

In addition to existing studies with a focus on societal attitudes toward social robots, there is a need to understand the views of professional caregivers and patients. This study used desired future scenarios to collate the perspectives of experts and analyze the significance for developing the place of social robots in care.

**Methods:**

In February 2020, an expert workshop was held with 88 participants (health professionals and educators; [PhD] students of medicine, health care, professional care, and technology; patient advocates; software developers; government representatives; and research fellows) from Austria, Germany, and Switzerland. Using the scenario methodology, the possibilities of analog professional care (Analog Care), fully robotic professional care (Robotic Care), teams of robots and professional caregivers (Deep Care), and professional caregivers supported by robots (Smart Care) were discussed. The scenarios were used as a stimulus for the development of ideas about future professional caregiving. The discussion was evaluated using qualitative content analysis.

**Results:**

The majority of the experts were in favor of care in which people are supported by technology (Deep Care) and developed similar scenarios with a focus on dignity-centeredness. The discussions then focused on the steps necessary for its implementation, highlighting a strong need for the development of eHealth competence in society, a change in the training of professional caregivers, and cross-sectoral concepts. The experts also saw user acceptance as crucial to the use of robotics. This involves the acceptance of both professional caregivers and care recipients.

**Conclusions:**

The literature review and subsequent workshop revealed how decision-making about the value of social robots depends on personal characteristics related to experience and values. There is therefore a strong need to recognize individual perspectives of care before social robots become an integrated part of care in the future.

## Introduction

### Background

#### Social Robots in Care

**Figure 1:**
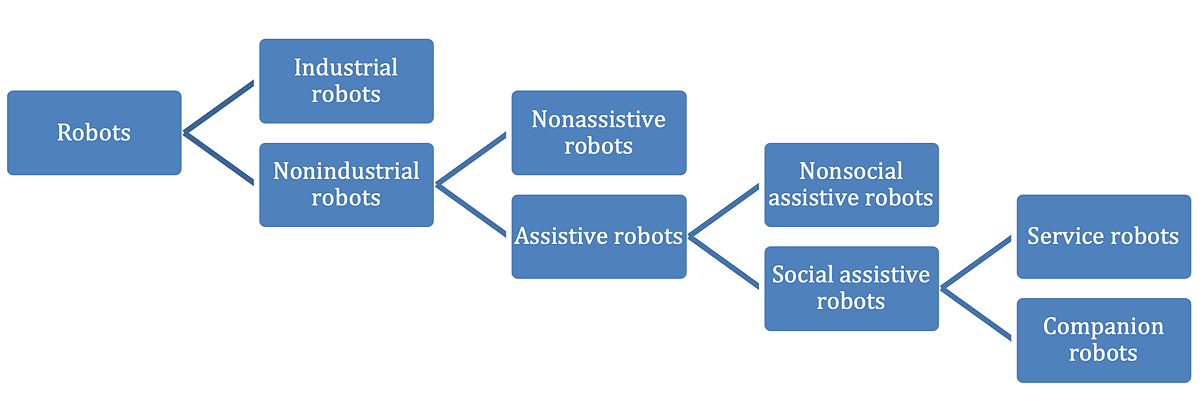
Focused representation of the subdivision of the different robot types [4].

Robotics represents a special case of technology use. Industrial robots are socially accepted to relieve humans from hard work [1]. We here focus on nonindustrial robots, which are relatively more complex than industrial robots and cover a broader range of applications being deployed in many different branches of commercial or private use [2] to assist people, including in health care. Nonindustrial robots can be divided into assistive and nonassistive robots. Assistive health robots can be used for surgery, therapy, or care [3], and can be subdivided into social or nonsocial assistive robots. Social assistive robots can be service robots (eg, a lifting aid) or companion robots (eg, animal-like entertainment robots) (see Figure 1) [4]. They interact with people or work closely with them. Social robots differ from pure service robots in that they emulate human behavior while providing services, thus establishing a form of interpersonal communication. For example, a socially acting lifting aid would not only reposition patients but also ask compassionately whether the lying position is comfortable. In this context, “interaction” does not necessarily have to take place via spoken language but can also take place exclusively via social and emotional cues [2]. Hereafter, the term “robot” is used to refer specifically to social robots.

Robots are often researched in terms of their ability to express empathy and engage in interactive exchanges. Empathy can increase patient satisfaction and motivation for improvement as well as adherence to therapy programs in the patient-therapist interaction [5]. Robots simulate empathy mostly by facial and verbal expressions [6]. A small-scale study (N=36) found that people interacting with robots equipped with an “empathic module” communicated over a longer period of time and perceived the robots as more trustworthy, intelligent, and empathic than the control group who interacted with a robot that did not express empathy. In addition, the participants that interacted with the “empathetic” robot had a stronger sense of knowing the robot and perceived the interaction as comfortable [7]. However, another study showed that if there were incongruities between the affective state of the user and the emotional reaction of the robot, users rated the robot negatively [8]. A comparative study found that people were kinder to a robot that emulated empathy relative to the comparison group [4].

#### Growing Interest in the Use of Social Robots in Care

There are three core reasons for the growing interest in the use of robotics in care.

First, the demand for professional caregivers is likely to outstrip the supply [9]. Professional caregivers (ie, those working as trained specialists in the fields of health and nursing care or geriatric care in rehabilitation facilities, hospitals, and nursing homes, as well as in outpatient care in the home environment and related areas) are in demand in times of an aging population. In Switzerland, 367,000 people are being cared for by professional caregivers, which accounts for 28.9% of the population aged 80 or above; in 2018, 92,000 people, including 15.3% of the population older than 80 years, lived in a nursing home [10]. In the coming years, the availability of care is likely to decline as population growth slows and life expectancy increases. More people will need health care while fewer family members will be available to provide support. Due to the declining birth rate, future generations will consist of fewer people who could potentially take on the care of their parents or grandparents [11]. Other factors making it more difficult for family members to provide care include the rise in women’s employment, with women traditionally taking on the caring role, and the growing mobility of future generations [11]. According to population forecasts, the percentage of people over 67 years of age will rise steeply [12]. The probability of neurodegenerative or chronic illness increases with age, which accordingly increases the care dependency of the population [13]. Assistive technologies may be one way to maintain care and ease this tense care situation. Robots may compensate for functional losses experienced by older people and promote everyday skills that help to maintain independence [2]. Robots can also enhance patient adherence to medications and exercise [14].

Second, robotics offers ways to increase the autonomy of service recipients. Elderly people are impaired in their autonomy if they are no longer able to perform certain activities independently, such as preparing food, housework, telephoning, and shopping. For 16% of people living at home in Switzerland aged 65-79 years, one or more of these activities are either impossible or very difficult; 32% of people aged 80 years and over confirmed this statement, and minor difficulties were reported by 14% and 24% of the respondents aged 65-79 years and 80 years or older, respectively [15]. This shows that care often does not necessarily include physical care but that people also need support in areas that may be improved by technological assistance [3].

Third, robotics may take over the heavy work that puts a strain on professional caregivers. Three-quarters of professional caregivers state that heavy physical work is very common or frequent in their daily work [16]. Professional caregivers also report a high workload and associated stress. There is a mismatch between the workload and the time available [13]. Robots can relieve caregivers of the workload by supporting or even completely taking over heavy physical work tasks, thus saving time [3].

#### Acceptability of Social Robots

The development and potential of robots are still at an early stage. In 2018, 271,000 service robots were sold, representing a rise of 61% as compared with the market in 2017 [17]; however, implementation is progressing slowly in robotics as in other eHealth interventions [18]. The implementation raises many ethical questions such as data protection, responsibilities, or trust [19]. Many studies on barriers and facilitators have been published in recent years [20], indicating that concepts and applications should be widely debated by developers, experts, and users to ensure feasibility. Social acceptance is currently still low. For example, in the Eurobarometer survey, participants (N=26,751) generally had a positive attitude toward robots, but not in all areas: 27% stated that robotics should not be used in the health sector [21].

To maximize the benefits of using robots in health care, and especially in professional caregiving, it is necessary to learn about the attitudes and acceptance of these robots by society and health care professionals. A study has shown that people assess robotics as an opportunity. They expressed criticism of scenarios where robots performed care services for close relatives but liked the idea of robots undertaking dangerous tasks. The assessment of robots revealed only minor differences between different age groups, in which younger people were slightly less anxious about robots than the older or middle-aged groups [22]. Studies of perceived usefulness and perceived ease of use, which are critical to robot acceptance, are currently rare [23].

In speculating whether robots will have a role in providing care, we need to consider acceptance by both service recipients and professionals. This depends on the roles that robots undertake and the potential for expressing empathy [24,25]. The development of empathic robots is still at an early stage. To promote this development, we need to understand the factors that contribute to better care from the perspective of potential users of robotics. To determine whether robots are acceptable and how future caregiving can be designed, we organized a workshop with a series of focus group interviews among a diverse group of participants using future scenario planning.

### Aim of the Study

This paper focuses on the development of a vision for the use of robotics in geriatric social and health care. It addresses the potential of social robotics to augment care for older people and supports the work of professional caregivers. This vision was developed during an expert workshop, including participants from Switzerland, Austria, and Germany, who have experience in the issues related to the care for older people. The workshop was based on four future scenarios, which served as inspiration for the participants’ imagination regarding the possible changes in the field of professional caregiving through the use of social robots. The scenarios were developed in advance of the workshop using existing evidence from the literature. The workshop focused on potential and desirable future scenarios and on the steps required to prepare and implement these scenarios by 2025.

The aim of the workshop was to gather the views of health care professionals and educators, (PhD) students, patients, developers, scientists, and governments regarding the potential of robot use in future care and to stimulate debate and research on their use.

The research questions were: (1) Which (robotic) care scenario do the experts consider likely for 2025? (2) Which (robotic) care scenario do the experts consider desirable for 2025? (3) What can we do to make this scenario a reality?

## Methods

### Scenarios

An expert workshop (Careum Dialogue 2020) supported by the Careum Foundation was held in Zurich in February 2020. The workshop was designed according to the scenario method, which is suitable for statements about future goals with special consideration of the influencing factors and their effect on the goals. Consequences for future actions are to be derived from the scenario method. For this purpose, a best-case scenario and a worst-case scenario are formulated, which limit the range of conceivable developments and deviate from the long-term trend into positive or negative outcomes [26]. However, since the workshop participants had to discuss which scenarios represent the worst and best cases, two poles were formed: Analog Care (professional caregiving without any technical support) and Robotic Care (professional caregiving performed by robots without humans). In the sense of the scenario method, it is possible to define further characteristics between the poles of best-case and worst-case scenarios. However, the following criterion must be taken into account in the creation: the developments within the different scenarios must not cancel each other out. Furthermore, the scenarios must not be susceptible to collapse due to changes in minor factors. The extreme scenarios have the highest possible degree of severity and are thus as close as possible to the edges of the funnel of the possible future [26]. To support the experts in their discussion, two further scenarios were added: the scenario of professional caregivers supported by robotics (Deep Care) and the scenario in which both professional caregivers and robots work in teams and perform tasks independently (Smart Care). These scenarios are positioned between the two extreme scenarios described above (Analog Care and Robotic Care). This was intended to improve understanding of the detailed different attitudes of the participants.

The scenarios used in this workshop were created with the help of literature collected by searching the PubMed and Google Scholar databases using the search terms “caregivers,” “care,” “robot,” “future,” “scenario,” “vision,” “utopia,” and “dystopia.” The development included all articles (N=28) dealing with the preferred care scenarios of the future, possibilities of robot use in care, ethical aspects of robot use in health care, changes in care through digitization, and the acceptance of robots (see Multimedia Appendix 1). Despite the wide range of scenarios, special attention was paid to developing the scenarios as “simple” scenarios. In the literature, “simple” scenarios are characterized in particular by few factors in their construction and description [27]. The reduced complexity in the description, even during the workshop, should allow a short time for the explanation of the scenario construct and create a larger scope for discussion. Thus, the scenario method enables the development of a spectrum of future visions. Participants were able to develop their own scenarios within this spectrum in the workshop, corresponding to their own ideas. The scenarios developed in advance served as support for creativity.

The scenarios were created according to the recommendations for scenario development by Fink and Siebe [27]. The addressees were experts from the health care sector in Switzerland, Germany, and Austria, who were to be supported in orienting the planning of future health care in politics, practice, and society. The workshop served as an orientation situation, which is distinct from a decision situation, and is not necessarily associated with a concrete decision between several alternatives for action but rather serves to orient and prepare for future decisions [27]. The scenarios were initially regionally limited to German-speaking countries, since the experts are employed in this field and regional particularities such as legal regulations or training guidelines are decisive when considering future developments in the health care system.

### Data Collection

The data were collected by the experts using a flip chart exercise and detailed field notes of the group discussion.

In the first round, one specific scenario was assigned to two tables each as the subject of debate. The advantages and disadvantages of Robotic Care, Analog Care, Smart Care, and Deep Care were addressed. The guiding questions for the first round of the group discussion were: (1) Has the scenario been described properly? (2) How likely is the scenario? (3) How desirable is the scenario?

In the second round, seating arrangements were changed according to plan so that experts from each scenario who sat at different tables beforehand now sat together. They briefly presented the discussion held at their table and began to talk about the likelihood and attractiveness of the different scenarios as well as the factors accelerating or limiting this health care future. Subsequently, the participants began to build their own health care scenarios such as a mix of the options given or a completely new alternative. The guiding questions for the second round of the group discussion were: (1) Which scenario is the most likely/desirable scenario? (2) Which aspects can accelerate or slow down the scenario? (3) Is there any other scenario you can imagine?

The groups at the eight tables reassembled again after this round. The last round focused on possible ways to compile and implement robotics in future professional caregiving. The question for the last group discussion was: Which steps are needed for implementation?

**Figure 2:**
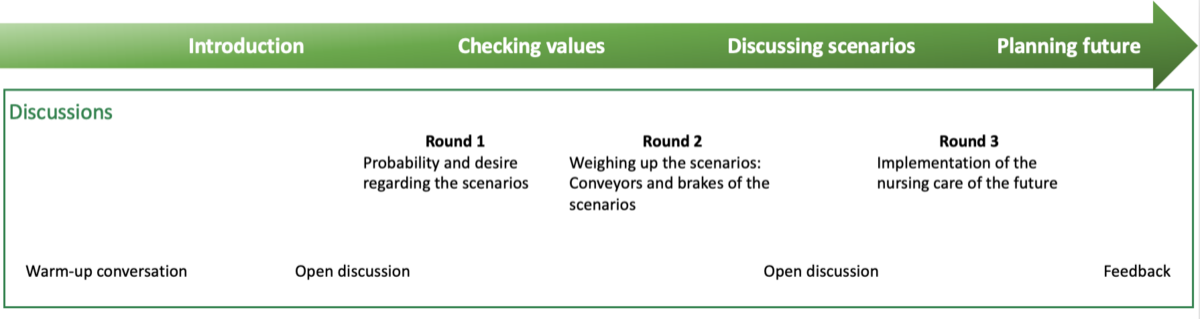
Workshop design.

The procedure of the workshop is shown in Figure 2.

To stimulate discussion, keynote speeches were given by experts between the various rounds. These dealt with the possible areas of application of robots, artificial intelligence, ethical requirements, the collaboration of robots with health care professionals, and the social interaction between robots and humans. Transcripts of the keynote speeches can be found in Multimedia Appendix 2.

### Data Analysis

For evaluation, the research team’s records of the plenary discussions, the presentation of the respective discussions, as well as the sequential records of the discussions at the tables were analyzed using qualitative content analysis. The notes of the experts during the discussion at the tables were also included in the content analysis. The content analysis was carried out using MAXQDA line by line, sentence by sentence, or section by section according to the strategy of the inductive category formation described by Mayring [28]. With this approach, categories were formulated directly in the material from text passages that were evaluated according to the category definition. After a first step of category formation, some categories could be generalized step by step into main categories by means of the summary. A total of 176 codes were identified (85 in the authors’ notes and 91 in the participants’ notes). These codes were organized into a common category system with five superordinate categories and a total of 25 subcategories and 21 subsubcategories. The following superordinate categories were formed: attitude toward robotics (25 codes), requirements for the care of the future (22 codes), discussion of scenarios (33 codes), implementation of professional caregiving in the future (20 codes), and development of professional caregiving in the future (76 codes).

The superordinate categories were based on the main components of the workshop: the recording of the general attitudes of the participants toward robotics, illumination of the needs for future care, discussion of the scenarios created in advance, as well as the development and implementation of the care in the future. The subcategories were also derived from the analyzed data according to the main focus of the workshop. For example, one subcategory contained all statements on the Robotic Care scenario. In turn, the subsubcategory dealt with different versions of the statements on the Robotic Care scenario, such as concern about the danger of two-tiered medicine. All statements made by participants that dealt with a concern in this respect were summarized in this subsubcategory.

### Ethical Aspects

Ethical principles of research in accordance with the Helsinki Declaration were observed [29]. The participants did not suffer any damage or impairments. The anonymity of the data was guaranteed by the form of data collection. Discussions were documented anonymously. Notes on flip charts were photographed and written down. The lecturers mentioned by name were asked for their consent. The participants had already been informed in the invitation about the focus of the workshop and the data collection planned. Participation was subject to consent.

## Results

**Figure 3:**
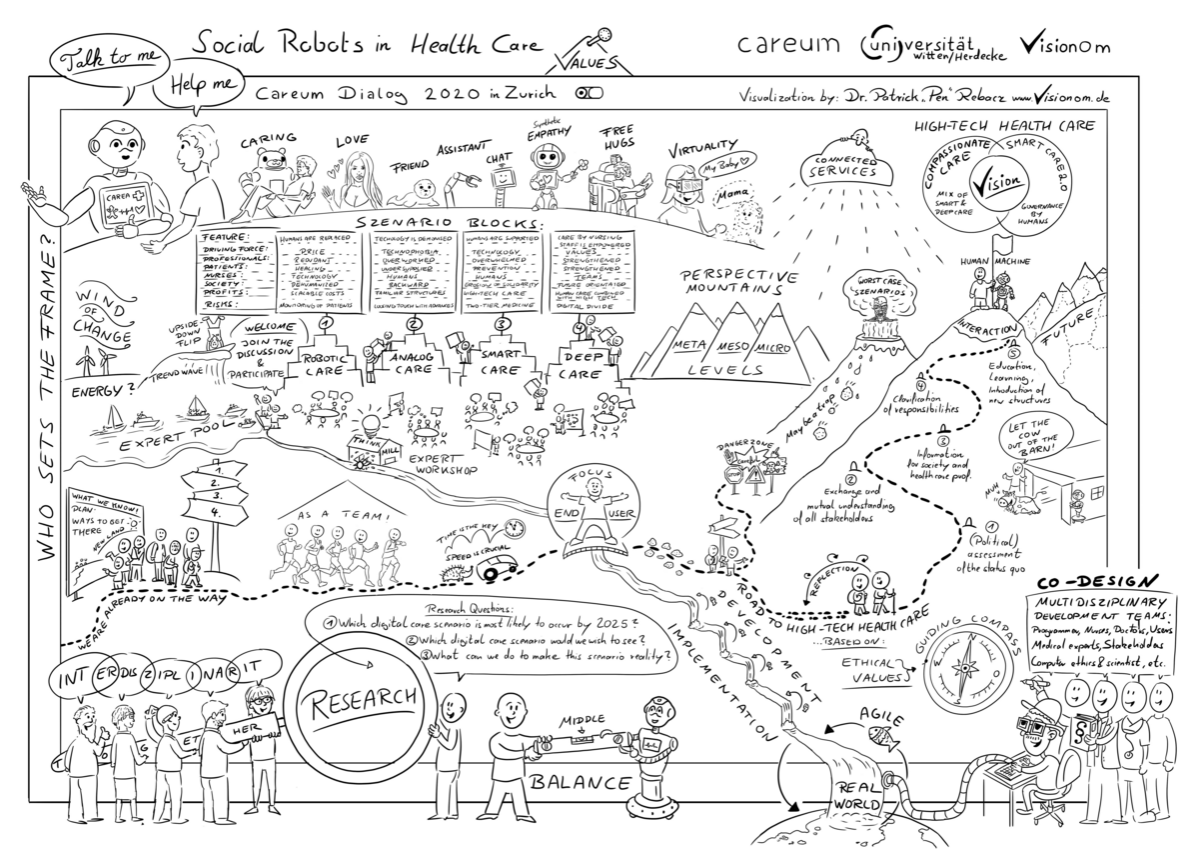
Graphic recording of the Careum Dialogue 2020 workshop.

Figure 3 shows the various discussion contents of Careum Dialogue 2020 as a graphic recording. This graphic was presented in the follow-up workshop by one of the authors, who also took part in the discussion, based on the qualitative content analysis and serves here as a short overview before explicitly answering the research questions and presenting the further results.

From the experts’ point of view, either the occurrence of the Smart Care scenario (teams of professional caregivers and robots) or a mixture of the Smart Care and Deep Care scenarios (professional caregivers supported by robotics) were the most likely (robotic) care scenarios for 2025. From their point of view, such a development would be close to current health care and would enable professional caregivers to support patients without depriving them of the human contacts that are crucial for their well-being. The two extreme scenarios were felt to be unlikely. The experts saw neither sufficient technical nor social prerequisites for the Robotics Care scenario to occur. In their view, the current state of technical development does not allow for such a complete takeover of care by robots. The experts also stated that not only technical progress was necessary to enable changes in the future but also the progress of society. For example, it would be necessary to implement training in eHealth literacy for the acceptance and critical evaluation of technology in the health care sector. The experts saw the occurrence of the Analog Care scenario as unrealistic owing to the limited human resources. In addition, they believed that a complete departure from technology would mean a step backward for both professional caregivers and patients, which would not be supported by society.

As part of the discussion on the most desirable (robotic) care scenario for 2025, the experts initially developed two scenarios on their own in small groups (Compassionate Care and Deep Care 2.0). These scenarios were based on the predeveloped Deep Care and Smart Care scenarios and supported the discussion about the various aspects of a desirable future scenario. For the future, the experts wished for the support of professional caregivers by technology and robotics. The experts believed that a combination of technology and professional caregivers is unbeatable, as technology could act on the latest evidence to support evidence-based care that leads to better results. In the experts’ view, professional caregivers could thus possibly provide higher-quality care according to “traditional” values: safety can be increased by robotic support; caregivers can spend more time with patients by using robots to relieve them of routine tasks; patients’ autonomy can be improved and extended by robotic support in their home environment; and patients can put together their own individual range of care services, which can consist of robotic and personal support. It was stated that patients might like the choice between robotics or professional caregivers, depending on their situation and the daily condition. In the debate, the experts mentioned that patients may perceive personal assistance as a burden at times. As an example, it was expounded that on some days, personal interaction and corresponding necessary courtesy are not pleasant. In addition, the experts reasoned that while health professionals may treat people differently depending on personal sympathies or other factors, robots might treat all patients equally.

The experts saw health care professionals as users whose acceptance is critical to the project. In addition to personal attitudes toward technology, the experts also considered education and the development of skills to be relevant for a high level of acceptance. Therefore, experts also advocated improving education and training to better prepare professional caregivers for this work. The long time needed for education and training while technology is changing so rapidly could be a problem in realizing this scenario. The experts saw the opportunity to enhance the value and reputation of health care through technology as the greatest benefit of this scenario. Professional caregivers could benefit from a redistribution of power as a result of the increasing use of digital tools. They could also benefit from a new professional profile that would attract people who are both highly socially oriented and technically interested.

By uniting humans and technology, a two-tiered health care system could be avoided, which in the experts’ view could develop through an increase in technical support. However, the experts were not sure whether the first-class health care would be robotic or personal health care.

The Robotic Care scenario was considered problematic because of the danger of two-tiered medicine and uncertainty about who would have the power to make decisions in this environment. The experts described care as highly individual and sensitive work, and saw the danger that standardized decisions could not reflect this complexity in all areas due to the exclusive use of robots. Another counterargument was the description of self-determination as the highest value. People should be able to refuse health care at any time. The experts expressed the fear that a robot could restrict self-determination if it offered standardized care that could not be controlled by the patients.

Further, it was discussed that patient acceptance is likely to have a large role in the adoption of robotics in health care. Similar to the case for health care professionals, the experts also saw the care recipients as users of robotics. The experts assumed that their acceptance depends not only on the implementation of robotics (eg, robotics enabling individualized care) but also on the area of application of the robotics and the scope of decision-making authority.

The Analog Care scenario was not considered desirable owing to its backward-looking nature and the resulting heavy burden on professional caregivers.

In the discussion on how to achieve the scenario they perceived as desirable, the experts saw a need for change in politics, institutions, health care professionals, science, health insurance funds, and society. The theory-practice-transfer route was urgently requested by the experts. In the experts’ view, the hard road to high-tech health care based on ethical values therefore requires the following steps: (1) (political) assessment of the status quo; (2) exchange and mutual understanding of all stakeholders; (3) information for society and health care professionals; (4) clarification of responsibilities; and (5) education, learning, and introduction of new structures.

Within the framework of the analysis, the results were structured using the frequently used social science classification into three levels of analysis (macro, meso, micro) [30]. At the macro level, political systems or society as a whole are examined, whereas at the meso level, the focus is on parts of these systems such as institutions. At the micro level, individuals are analyzed along with their actions, decisions, and relationships.

At the macro level, the experts called for laws, norms, and regulations to be adapted to new needs. The financing of technical solutions and the setting of financial incentives for the further development of technical solutions must be made possible by political decision-makers. Education and training must also be comprehensively filled with new content at this level. For example, robotics and eHealth should be part of the curriculum. Such new guidelines could be developed by national groups with an international focus. The experts also saw a need for social change. A broad discussion on the desired care of the future was needed with the aim of defining values that are important to society.

With regard to the meso level, institutions will have to introduce several changes for a positive health care scenario to succeed. The experts recommended bringing together information from Switzerland, Germany, and Austria to gain an overview. An exchange of information would create a network that could help to deal with the complexity of the sectors and the requirements of interoperability. Health care professionals should use this network as well as scientists. The experts also stated that institutions should set up experimental wards where health care professionals and developers can explore and test specific applications. This coordinated development with innovation labs and simulation centers could generate positive examples for practice and training. Moreover, cocreation was seen as the best way to develop new solutions for the future. Feedback between science and practical experience was seen as helpful to fine-tune possible solutions. In exploring the potential uses of robotics, experts considered the scientific study of the impact of robots on patient well-being and health as essential.

At the micro level, the experts saw a great need for empowerment of professional caregivers and patients. Professional caregivers would need to understand why changes in health care can enhance the value of their jobs. This can be achieved through sensitization and qualification, combined with participation and transparency. At several points in the discussion, the question arose as to which tasks could be performed by robots. In the experts’ view, this indicates the need for a clearer definition of the professional caregivers’ profession as distinguished from other professions.

Challenges at all levels were seen in users’ unrealistic expectations, the possibility of collective standardization, and negative attitudes toward technology in the process of development. Communication might be another challenge. People of different professional groups need to find a mutual language to work together. The awareness of this fact is important for a successful work process.

## Discussion

### Principal Insights

During the workshop, the acceptance of robots, changes in the organization of work resulting from the use of robots, and new ethical and legal requirements were the main topics of discussion raised. These topics were also identified in a previous study as central challenges in the introduction of robotics [31]. The discussions were stimulated by the possible visions of the future. Ultimately, the experts spoke in favor of a future in which professional caregivers are supported by robots. The experts considered this scenario to be both the most desirable and the most probable.

The experts’ opinions regarding the most probable and desirable future scenario are in accordance with the results of other international studies. First and foremost, a similar sentiment was expressed in a workshop with 25 Australian research or health care experts regarding future prospects on the subject of digital health. The scenarios developed by the participants, which represented their wishes and ideas for future care, included the use of robots as a support for older people to maintain independence and health (interaction, housework, transmission of health data) as well as the use of robots in the field of public health care and diagnostics [32]. In addition, it is important to consider that the visions of the future described by the experts in the workshop coincide with the desires of health care professionals and trainees that have been collected in studies. For example, in one study, professional caregivers most frequently named the desired use of robots in the areas of play, occupation, and activity; support for functional mobility; and in the supply/disposal of materials [33]. In another study, medical and nursing students (N=178) described a desire to use robots to remind the elderly to take medication, monitor their health, and promote physical and mental exercise [34].

The topic of acceptance was a particularly prominent focus of the discussion during the workshop. The experts believed that user acceptance is decisive for further implementation. In this case, the users are the professional caregivers but also the care recipients. From the experts’ perspective, both groups should receive the necessary education and information to be able to assess and understand robotics and thus increase acceptance. Acceptance is also named in the literature as the decisive factor for the use of robotics [35] and is therefore discussed more intensively below.

The acceptance of robots must be considered separately for different user groups. First, we consider whether and how robots are accepted by the older population. A review of the use of robots in therapy and care found that robots were perceived positively by the older population [36]. They felt safer because the robots were able to detect emergency events. In addition, the potential of robots to improve social skills of the users or to alleviate loneliness and isolation was evaluated positively by older people. However, this positive acceptance of robotics is also accompanied by various fears: people named the fear of losing human contact, being deceived by robots with regard to their abilities, and the fear of infantilization by using robots as toys [36].

Second, the acceptance of users working together with robots is considered. For instance, in a review on the social acceptance of robots in different professional fields, 336 articles were extracted from four databases [37]. After consideration of the exclusion criteria, 39 articles remained to be included in the review. In general, the review for the health and social services sector indicated a much more positive basic attitude toward robots than in other sectors. However, the studies also found that people who had no experience with robots more often had a negative attitude toward robots [37].

Third, acceptance must also be considered in relation to the respective field of activity and the degree of decision-making power. The concerns of experts regarding automated decisions of robots that lead to care that is not value-congruent or not desired by patients could be similarly considered. Poulsen and Burmeister [38] tested a new framework for care robots: the robot should be able to provide value-sensitive, individually adapted patient care. The study investigated the willingness of the end users to trust the decisions of the artificial intelligence. They described different scenarios of care for elderly people with robotic support, which were linked to personal values (autonomy, respect, dignity, privacy, independence, social connectedness) of the person. In the first phase of the study, the interviewed experts (N=4), including a registered nurse, a robotics academic, a computer ethicist, and a computer scientist, were able to see how the robot behaved differently in the same situation depending on which of the values was the most important for the person receiving care. The same scenario was shown several times, but with the supervised person giving a different rating of the personal values. For example, a person who was particularly concerned with autonomy would ask for help at a later time than a person who had rated this value as less important. Three of the four people subsequently stated that they considered the robot’s care to be value-sensitive and that this would enable them to receive good and individual care. In the second phase of the study, subjects (N=102) were shown two of the scenarios from phase 1 in a slightly adapted form. After each value had been changed, a questionnaire was administered to assess whether the participants would accept the decisions of the robots and use them in their daily lives. In addition, it was asked how trustworthy the users rated the robot outside the scenarios. The participants rated the robot as trustworthy for the scenarios in the questionnaires (50/102 questionnaires were completely filled out). However, in considering the supply chain beyond the given scenarios, the majority of respondents (66%) were not prepared to trust the robot if they were not clear about how decisions are made. In addition, 82% of the respondents indicated that they would like to have the ability to change the way the robot makes decisions [38]. This shows that the willingness to trust a robot is strongly dependent on how comprehensible and controllable its decisions are.

In summary, from these studies, people generally have a positive attitude toward robots and want to interact with them. A review on the acceptance of robots supports this statement [39]. Nevertheless, the field of health care should be evaluated with high sensitivity and there is a need for further research to fully understand crucial factors for the acceptance of robots.

### Conclusion

Health care that is characterized by the combination of robotics and humans has great potential to support the independence of care recipients, improve health outcomes, and relieve the burden for caregivers. There are already some approaches to support professional caregivers with technology. The results of this workshop show that technology-supported care is the care of the future favored by experts. To determine the exact characteristics of this type of care in the future, it is necessary to ascertain the wishes of society in the German-speaking countries in addition to the wishes of the experts. It is also necessary to adapt the legal regulations to create incentives for technical progress, legally define the necessary competencies for the professional caregivers, and implement them in education and training with the help of suitable teaching materials [40,41].

Future research should focus on what society, and in particular those in need of care, demand for the care of the future. However, the perspective of professional caregivers is also critical to development. One review pointed out that existing studies on health care workers’ perceptions have mainly focused on the impact robots might have on their patients rather than on themselves [42]. Moreover, the development of assistive technologies within the framework of scientific projects should be carried out in multidisciplinary teams with the involvement of users in the sense of co-design [43-45]. The co-design process can help to increase the acceptance in society and thereby also of the users, and to develop the technology oriented to the needs of the users [46].

Thus, care recipients are enabled to use technical support in their everyday lives. In addition to the already existing research in the field of eHealth literacy and technology acceptance, it is crucial to develop approaches for the training and education of society adapted to the scenarios of the future and the respective settings.

## Appendix

Multimedia Appendix 1 Literature on the basis of which the scenarios for the Careum Dialogue were developed.

Multimedia Appendix 2 Presentations held at Careum Dialogue 2020.
