# Supplementary material for: Views on Using Social Robots in Professional Caregiving: Content Analysis of a Scenario Method Workshop
Source: J Med Internet Res. 2021 Nov 10;23(11):e20046. doi: 10.2196/20046 (PMC8663608; doi:10.2196/20046)
Supplement: Multimedia Appendix 1 [file jmir_v23i11e20046_app1.docx]

## Appendix 1

Apt, W., Bovenschulte, M., Hartmann, E. A., & Wischmann, S. (2016). Forschungsbericht 463. Foresight Studie "Digitale Arbeitswelt".

Battistuzzi, L., Sgorbissa, A., Papadopoulos, C., Papadopoulos, I., & Koulouglioti, C. (2018). Embedding Ethics in the Design of Culturally Competent Socially Assistive Robots.

Bedaf, S., Marti, P., & De Witte, L. (2019). What are the preferred characteristics of a service robot for the elderly? A multi-country focus group study with older adults and caregivers. Assist Technol, 31(3), 147-157. doi:10.1080/10400435.2017.1402390

Berg, M., Giehl, C., Koch, M., Memmel, M., Spellerberg, A., & Walter, R. (2019). Gutachterliche Stellungnahme zu den Auswirkungen künstlicher Systeme im Speziellen und der Digitalisierung im Allgemeinen auf das kommunale Leben in Rheinland-Pfalz 2050.

Caic, M., Avelino, J., Mahr, D., Odekerken, G., & Bernardino, A. (2019). Robotic Versus Human Coaches for Active Aging: An Automated Social Presence Perspective. International Journal of Social Robotics. doi:10.1007/s12369-018-0507-2

Caic, M., Odekerken, G., & Mahr, D. (2018). Service robots: Value co-creation and co-destruction in elderly care networks. Journal of Service Management, 29. doi:10.1108/JOSM-07-2017-0179

Fosch Villaronga, E., Tamò Larrieux, A., & Lutz, C. (2018). Did I Tell You My New Therapist is a Robot? Ethical, Legal, and Societal Issues of Healthcare and Therapeutic Robots. SSRN Electronic Journal. doi:10.2139/ssrn.3267832

Friends of Europe. (2017). Disruptive models of healthcare for Europe. Discussion Paper.

Gongora Alonso, S., Hamrioui, S., de la Torre Diez, I., Motta Cruz, E., Lopez-Coronado, M., & Franco, M. (2019). Social Robots for People with Aging and Dementia: A Systematic Review of Literature. Telemed J E Health, 25(7), 533-540. doi:10.1089/tmj.2018.0051

Institute for Alternative Futures. (2012). Primary Care 2025: A Scenario Exploration. Retrieved from http://www.altfutures.org/pubs/pc2025/IAF-PrimaryCare2025Scenarios.pdf.

Jungwirth, D., & Haluza, D. (2017). Information and communication technology and the future of healthcare: Results of a multi-scenario Delphi survey. Health Informatics Journal, 25, 146045821770425. doi:10.1177/1460458217704256

Lee, S., & Naguib, A. (2019). Toward a Sociable and Dependable Elderly Care Robot: Design, Implementation and User Study. Journal of Intelligent & Robotic Systems. doi:10.1007/s10846-019-01028-8

Lehoux, P., & Grimard, D. (2018). When robots care: Public deliberations on how technology and humans may support independent living for older adults. Social Science & Medicine, 211. doi:10.1016/j.socscimed.2018.06.038

Neves, B. B., & Vetere, F. (2019). Ageing and Digital Technology. Designing and Evaluating Emerging Technologies for Older Adults. Singapore: Springer Nature.

Nwosu, A., Sturgeon, B., McGlinchey, T., Goodwin, C., Behera, A., Mason, S., . . . Payne, T. (2019). Robotic technology for palliative and supportive care: Strengths, weaknesses, opportunities and threats. Palliative Medicine, 33, 026921631985762. doi:10.1177/0269216319857628

O'Brolchain, F. (2017). Robots and people with dementia: Unintended consequences and moral hazard. Nursing ethics, 26, 969733017742960. doi:10.1177/0969733017742960

Ojha, S., Williams, M.-A., & Johnston, B. (2017). The Essence of Ethical Reasoning in Robot-Emotion Processing. International Journal of Social Robotics, 10. doi:10.1007/s12369-017-0459-y

Pelea, C. I. (2019). The Relationship Between Artificial Intelligence, Human Communication and Ethics. A Futuristic Perspective: Utopia or Dystopia? Media Literacy and Academic Research, 2(1), 10.

Poulsen, A., & Burmeister, O. (2019). Overcoming carer shortages with care robots: Dynamic value trade-offs in run-time. Australasian Journal of Information Systems, 23. doi:10.3127/ajis.v23i0.1688

Rösler, U., Schmidt, K., Merda, M., & Merlzer, M. (2018). Digitalisierung in der Pflege. Wie intelligente Technologien die Arbeit professionell Pflegender verändern.

Savela, N., Turja, T., & Oksanen, A. (2017). Social Acceptance of Robots in Different Occupational Fields: A Systematic Literature Review. International Journal of Social Robotics, 10. doi:10.1007/s12369-017-0452-5

Shaw, J., Agarwal, P., Desveaux, L., Cornejo, D., Stamenova, V., Jamieson, T., . . . Bhattacharyya, O. (2018). Beyond “implementation”: digital health innovation and service design. npj Digital Medicine, 1. doi:10.1038/s41746-018-0059-8

Topol, E. (2019). Preparing the healthcare workforce to deliver the digital future.

Umweltveränderungen, W. W. B. d. B. G. (2019). Unsere gemeinsame digitale Zukunft. Zusammenfassun.

Vercelli, A., Rainero, I., Ciferri, L., Boido, M., & Pirri, F. (2018). Robots in Elderly Care. DigitCult, Scientific Journal on Digital Cultures, 2, 14. doi:10.4399/97888255088954

Vinanzi, S., Patacchiola, M., Chella, A., & Cangelosi, A. (2019). Would a robot trust you? Developmental robotics model of trust and theory of mind. Philosophical Transactions of The Royal Society B Biological Sciences, 374. doi:10.1098/rstb.2018.0032

Wirtz, J., Patterson, P., Kunz, W., Gruber, T., Lu, V., Paluch, S., & Martins, A. (2018). Brave New World: Service Robots in the Frontline. Journal of Service Management, 29. doi:10.1108/JOSM-04-2018-0119

World Health Organization. (2019). Report on the WHO Symposium on the Future of Digital Health Systems in the European Region
