# Supplementary material for: Views on Using Social Robots in Professional Caregiving: Content Analysis of a Scenario Method Workshop
Source: J Med Internet Res. 2021 Nov 10;23(11):e20046. doi: 10.2196/20046 (PMC8663608; doi:10.2196/20046)

Appendix 2

Speaker	Employer/Title	Translated Title (Original title)
Janowksi, Kathrin	University Augsburg<br>Faculty of Applied Computer Science<br>Chair for Multimodal Human-Technology Interaction	What are social machines already doing today?<br>(Was machen soziale Maschinen heute schon?)
Günther, Detlef	ETH Zürich<br>Vice President for Research and Industrial Relations	Are machines social?<br>(Sind soziale Maschinen sozial?)
Vayena, Effy	ETH Zürich<br>Health Ethics and Policy Lab<br>Bioethicist	(Social Robots in Health Care – ethical challenges)
Ehlers, Jan	University Witten/Herdecke<br>Faculty of Health<br>Department of Medicine<br>Chair for Didactics & Educational Research in Healthcare	Care scenarios in a digital world. On the way to a utopia or dystopia?<br>(Pflegeszenarien in einer digitalen Welt. Auf dem Weg in eine Utopie oder Dystopie?)
Korn, Marc Oliver	University Offenburg<br>Affective Cognitive Institute<br>Chair for Human Computer Interaction	Emotions and AI – how machines learn to interpret human emotions.<br>(Emotionen und AI – wie Maschinen lernen, menschliche Emotionen zu deuten)
Bendel, Oliver	University Nordwestschweiz<br>University for Business Informatics	What makes robots social?<br>(Was macht Roboter sozial?)
De Witte, Bart	Founder of the HIPPO AI Foundation & Digital Health Academy in Berlin	Synthetic empathy: The robot's new clothes<br>(Synthetische Empathie: Des Robots neue Kleider)

The order of the table is based on the order of the presentations at the workshop. The slides of the talks (except the slides of Bart de Witte) can be seen below. Bart de Wittes talk is available as a webtalk: https://www.youtube.com/watch?v=jYpfUjMUD_Y

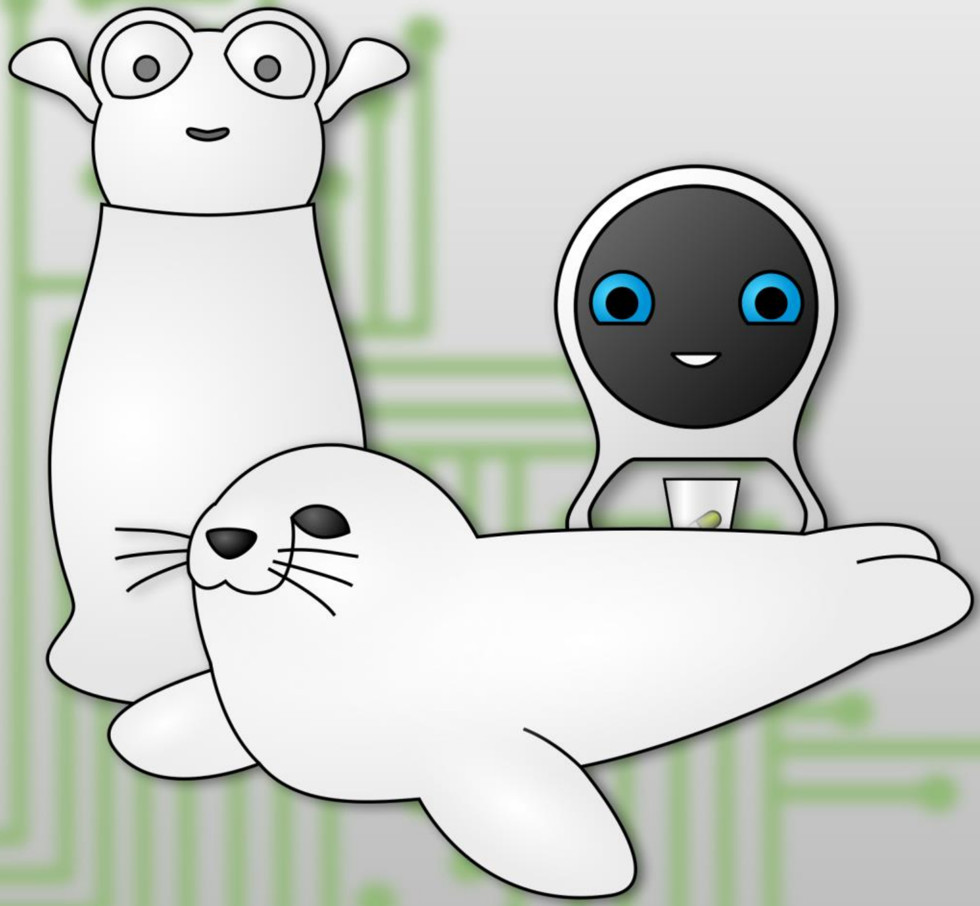

Universität Augsburg
Fakultät für Angewandte Informatik

Was machen soziale Maschinen heute schon?

Kathrin Janowski, M.Sc.

Lehrstuhl für Multimodale Mensch-Technik Interaktion

13. Februar, Careum Dialog 2020, Zürich

viva ForGenderCare EmmA emotional mobile assistant

Überblick

1 Die Maschine als Verwalter

2 Die Maschine als Vermittler

3 Die Maschine als Vertrauter

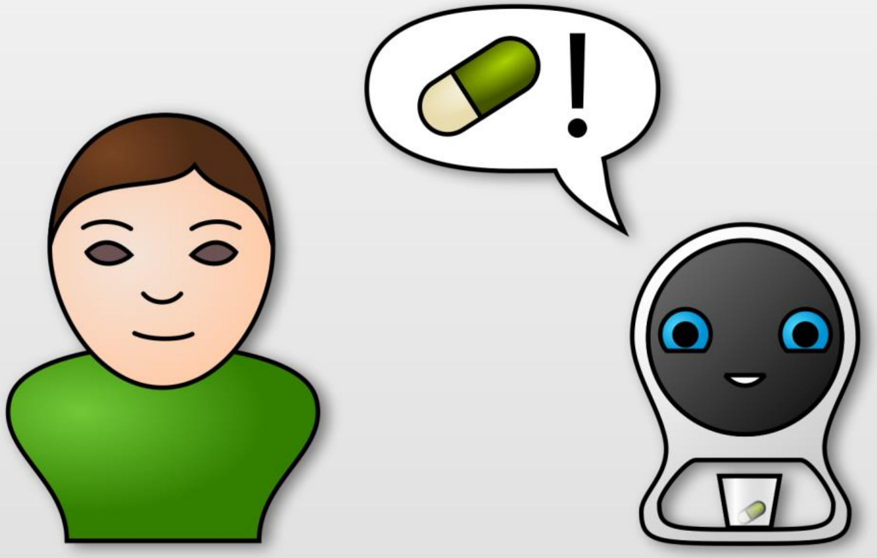

01

DIE MASCHINE ALS VERWALTER

Verwalter

- Erinnerung
- Empfehlung
- Erklärung

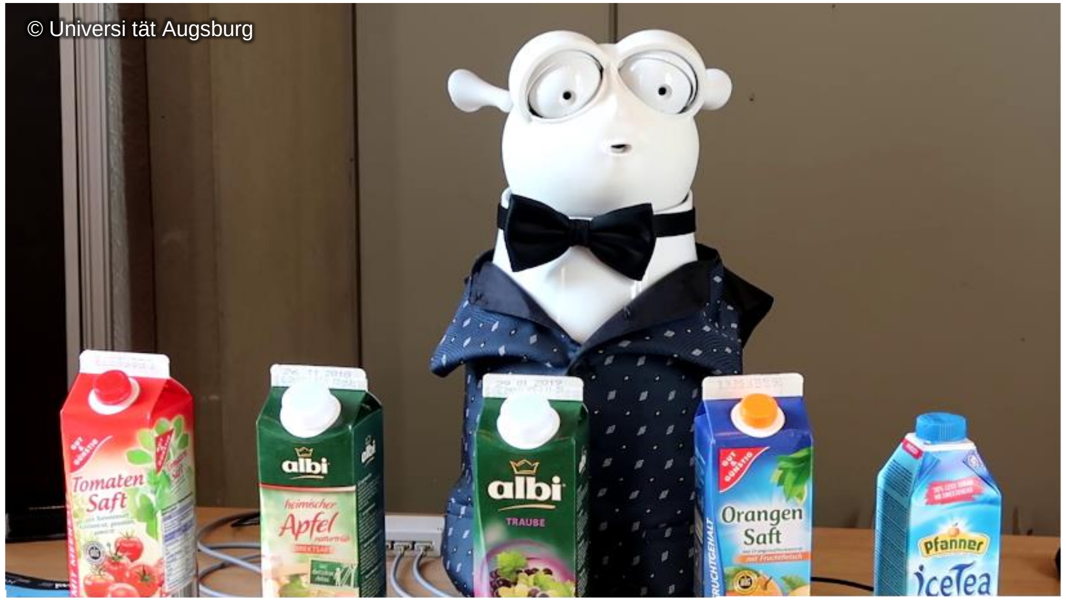

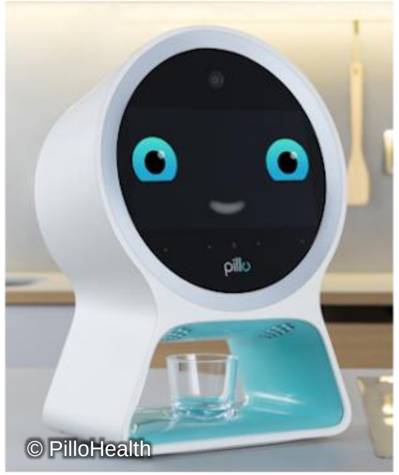

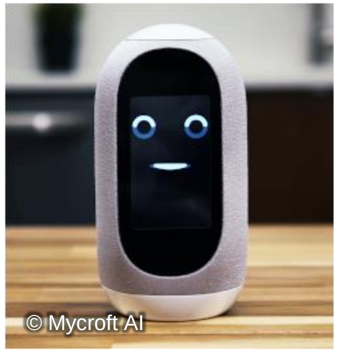

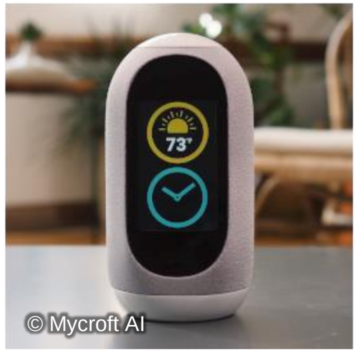

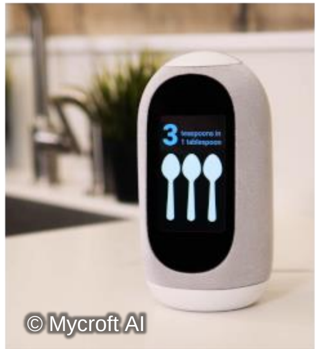

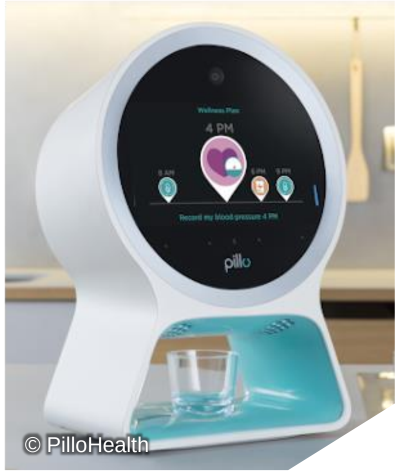

Kernkompetenz: Anpassung

- Argumentation
- Auftreten
- Angewohnheiten

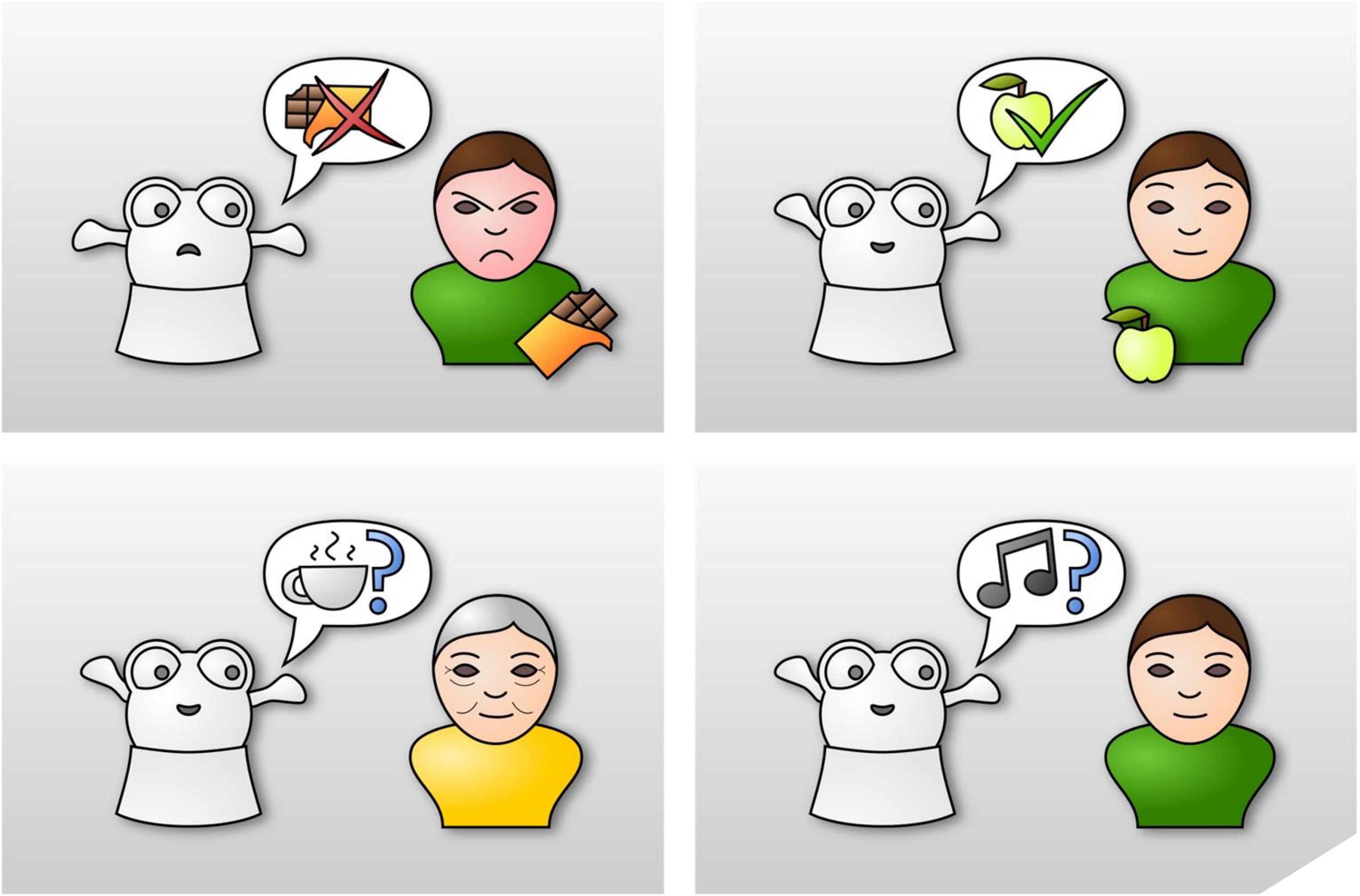

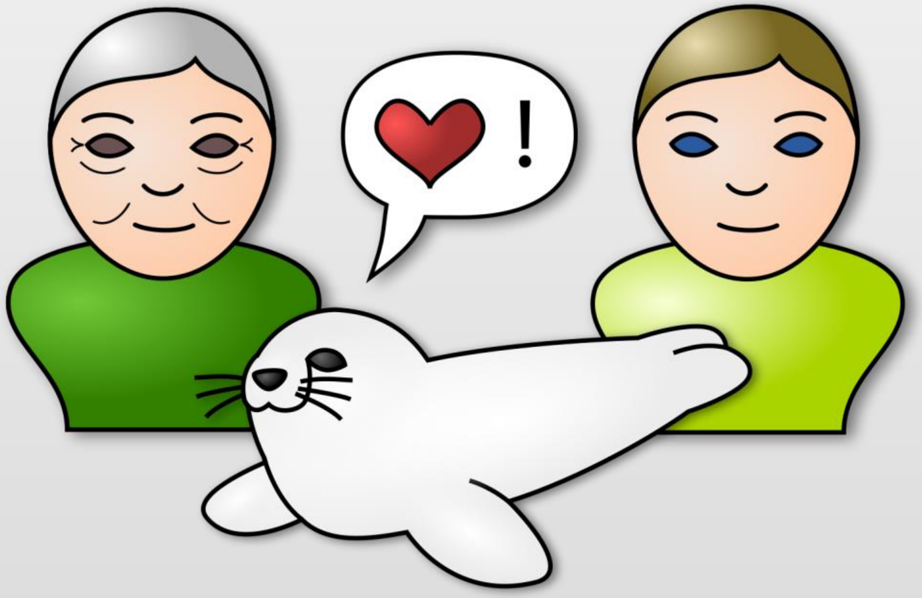

02

DIE MASCHINE ALS VERMITTLER

Vermittler

- Stimulation
- Schulung
- Stellvertreter

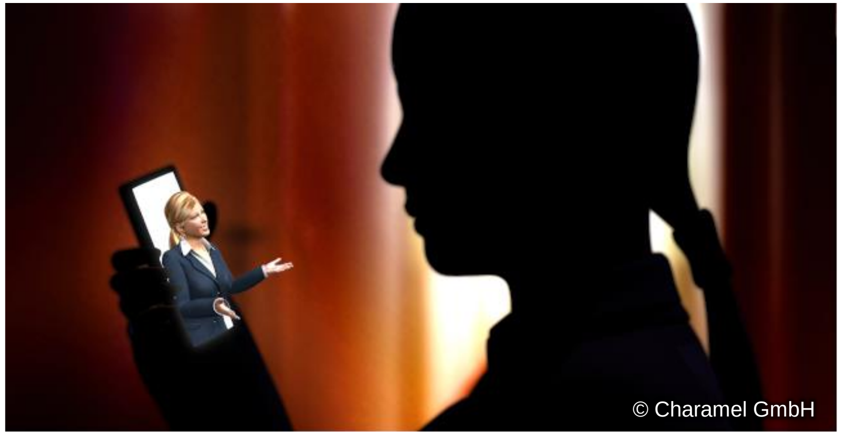
© Charamel GmbH

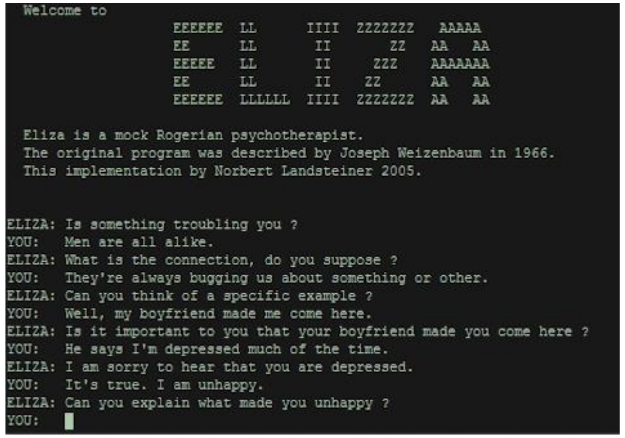

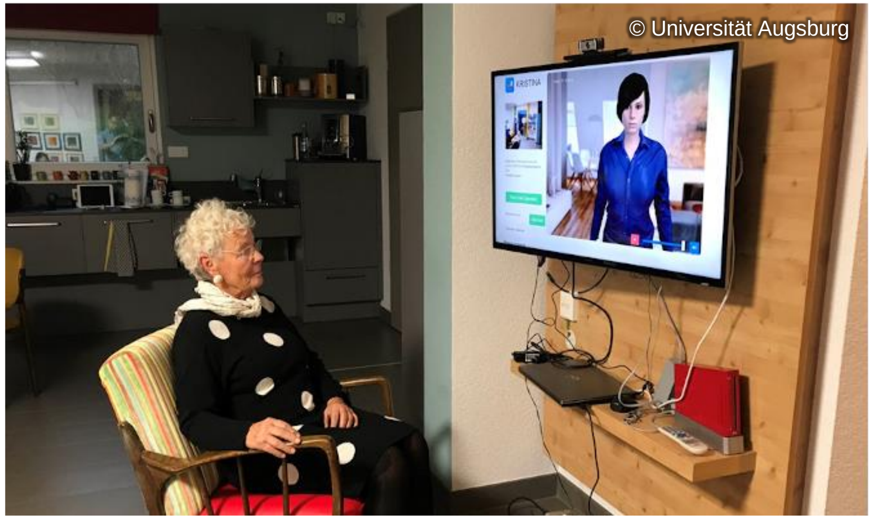
© Universität Augsburg

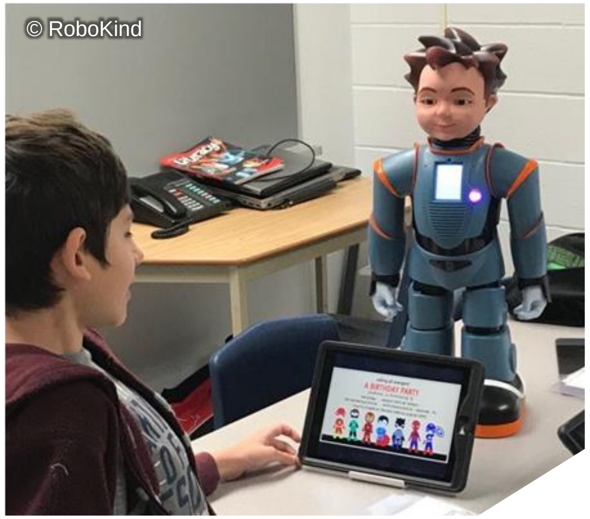
© RoboKind

Kernkompetenz: Emotionen

- Expressivität
- Erkennung
- Empathie

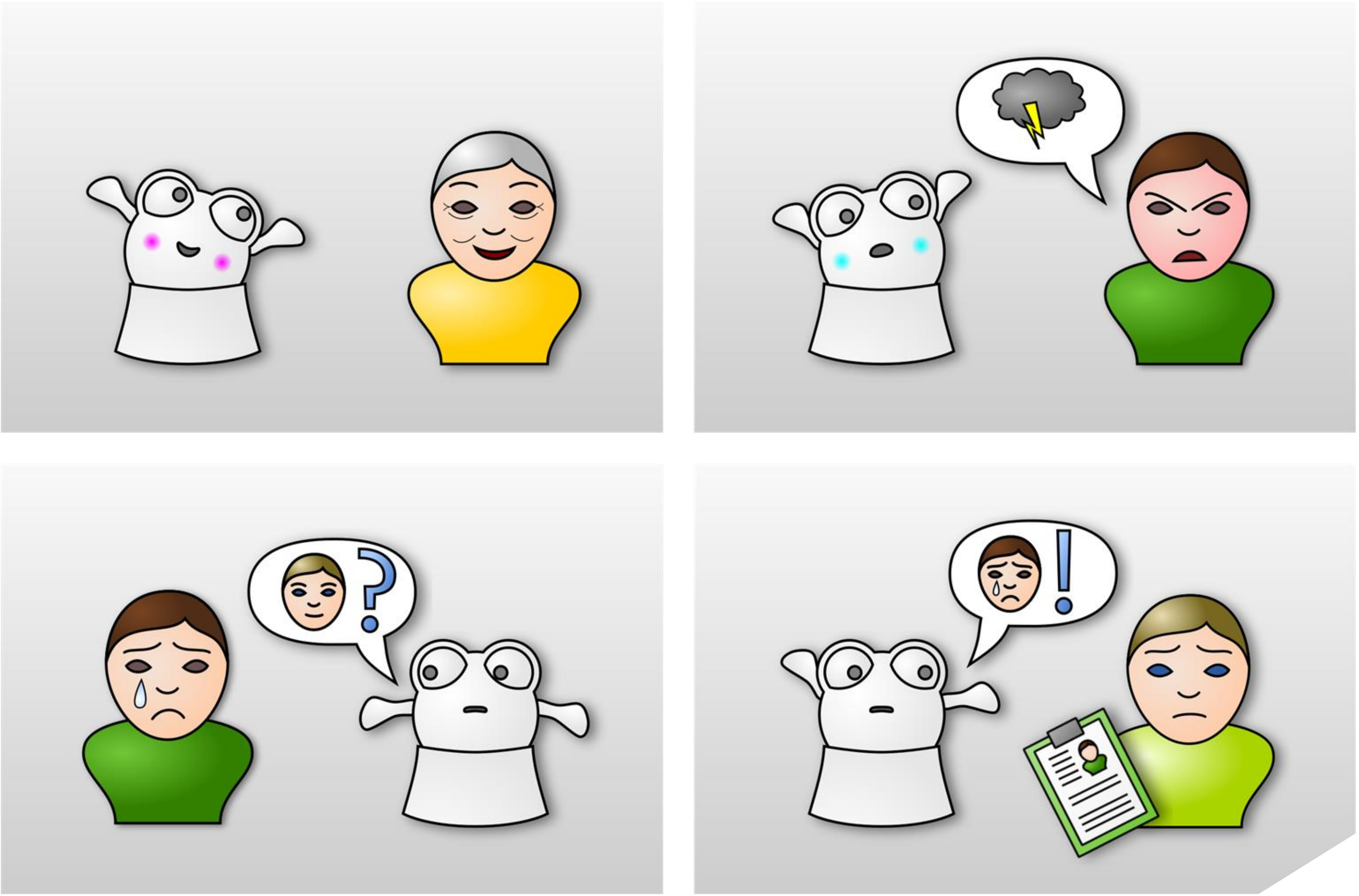

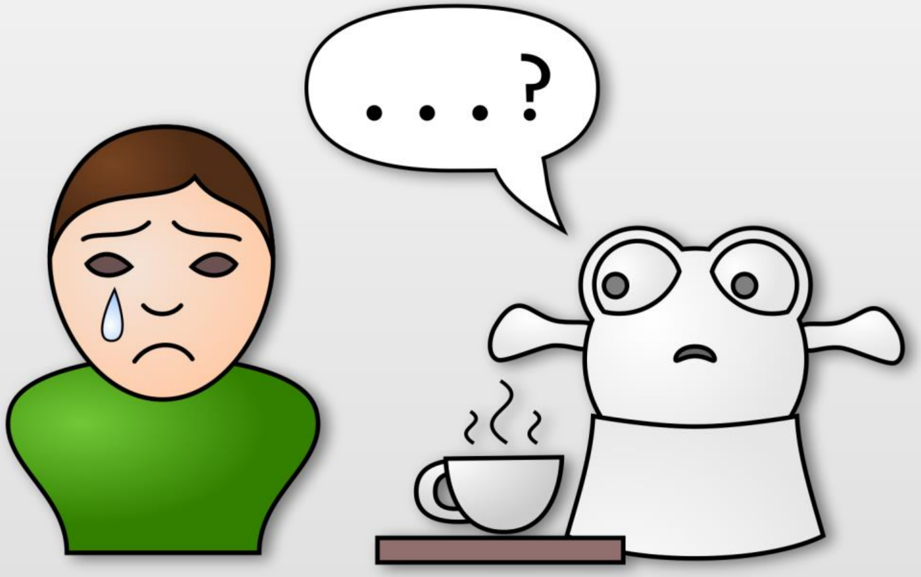

03

DIE MASCHINE ALS VERTRAUTER

Vertrauter

- Beschäftigung
- Begleitung
- Bindung

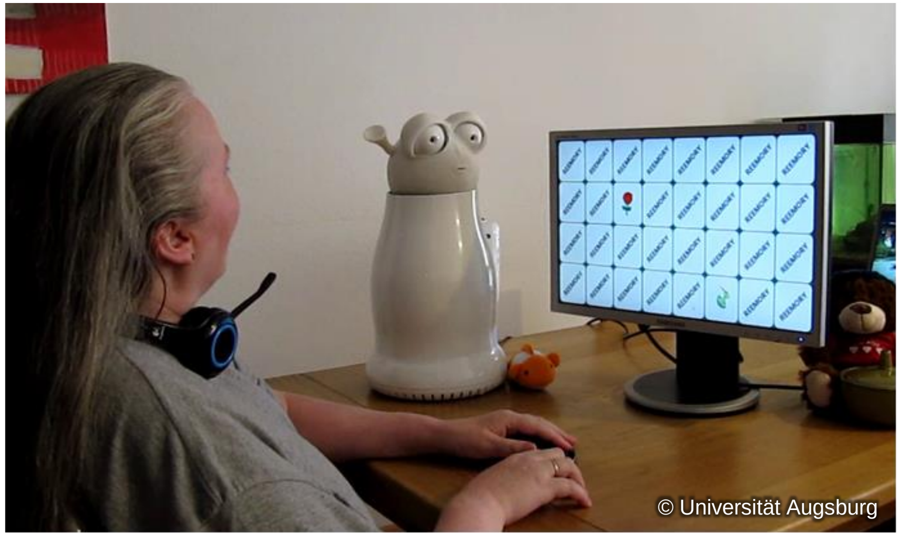

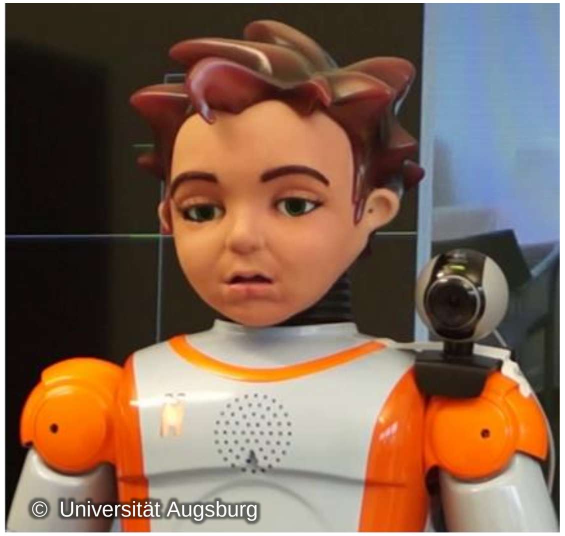

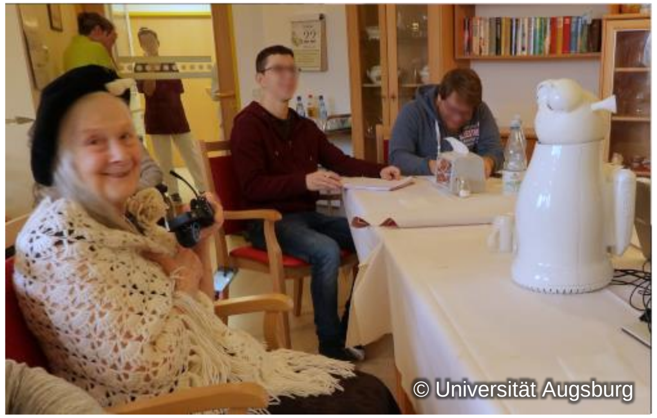

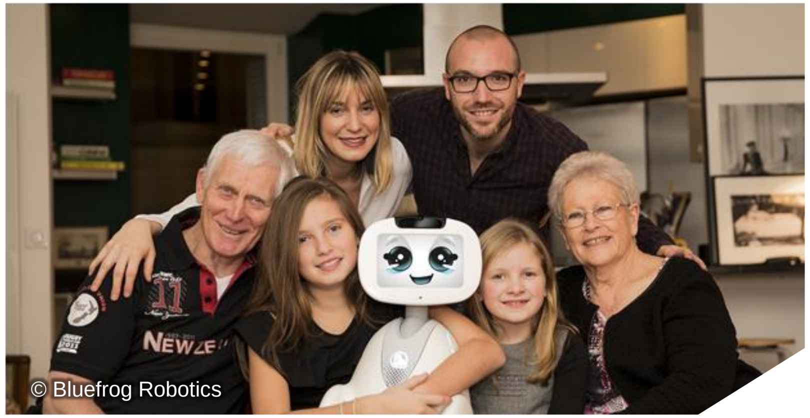

Kernkompetenz: Gemeinsamkeiten

- Geschichte
- Gespräche
- Gefühle

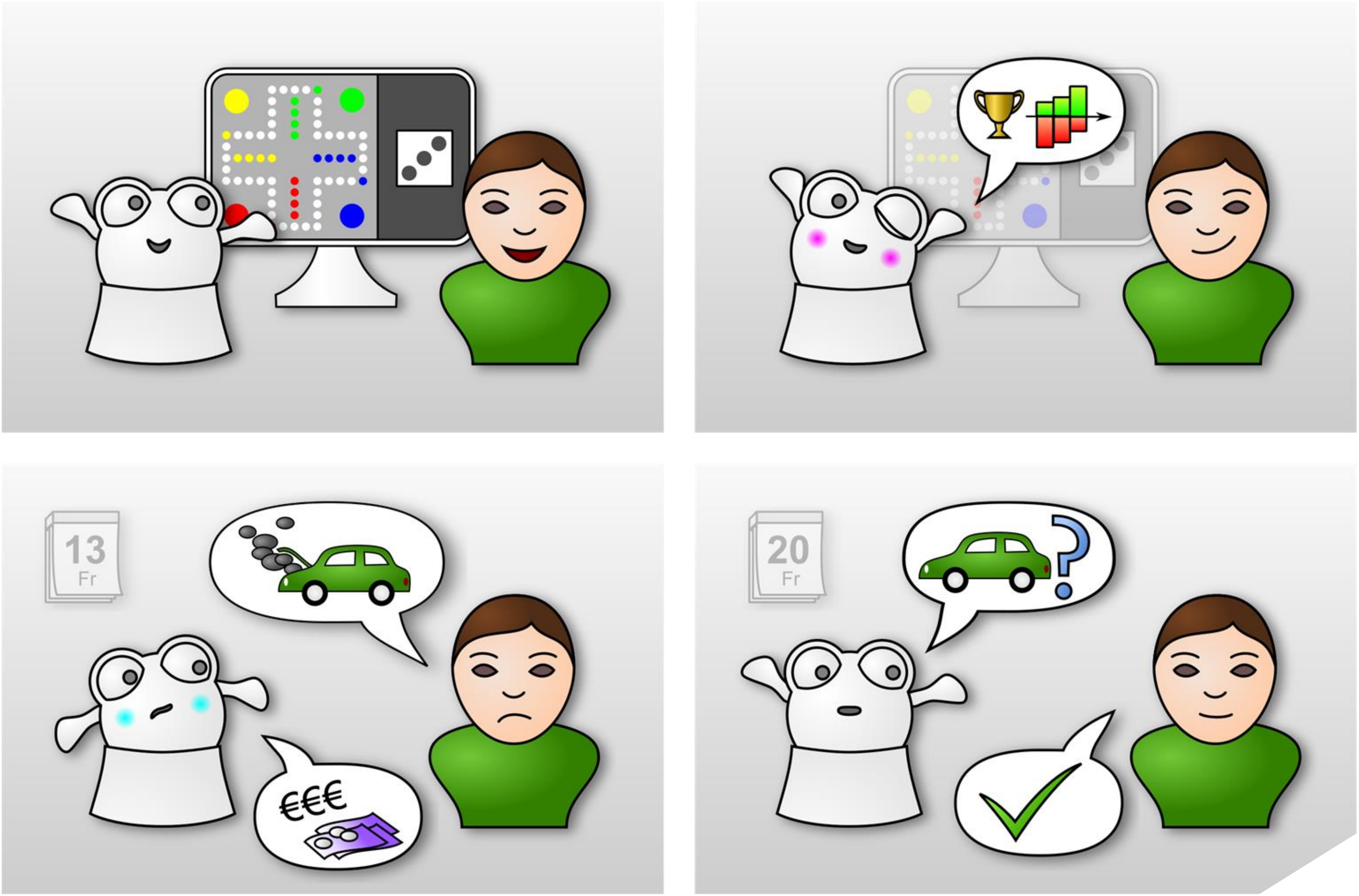

Fazit

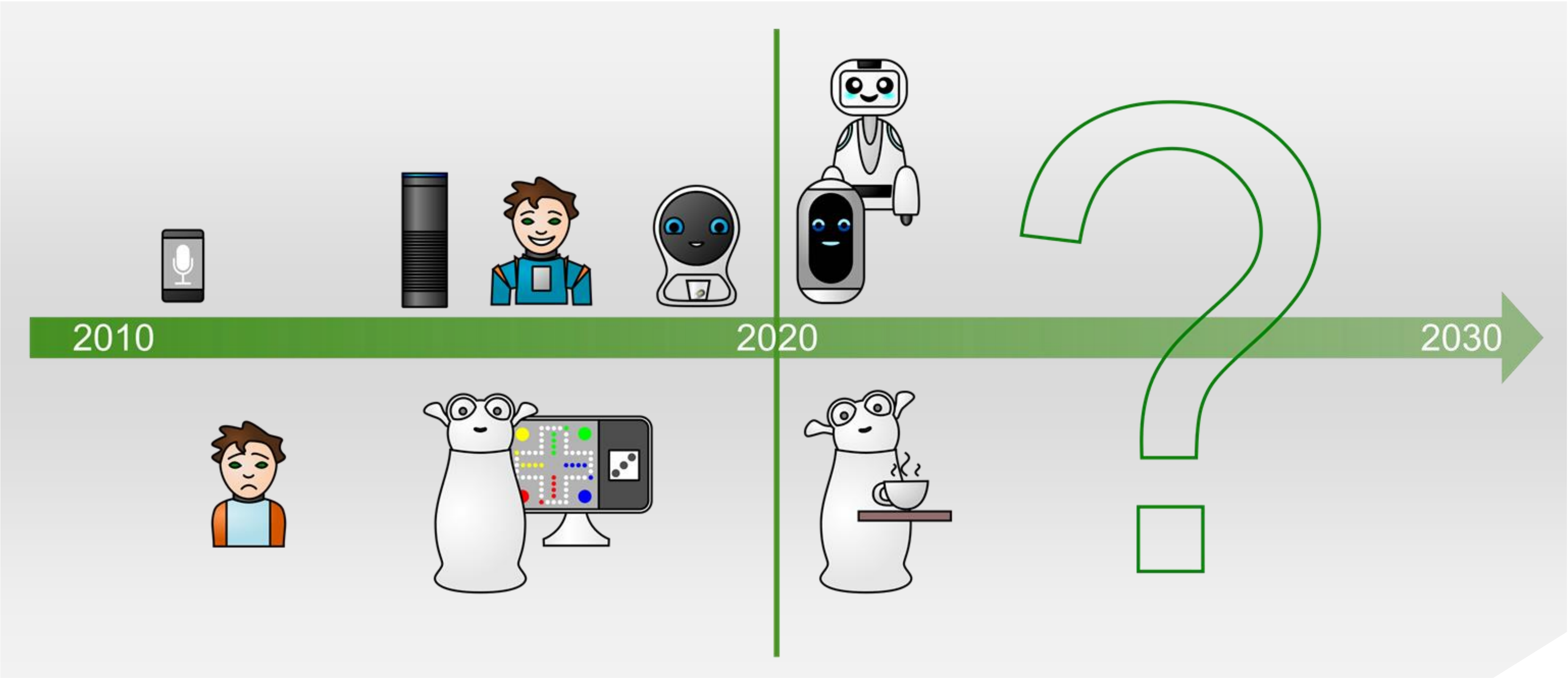

Bayerisches Staatsministerium für Bildung und Kultus, Wissenschaft und Kunst

ForGenderCare

GEFÖRDERT VOM

Bundesministerium für Bildung und Forschung

viva

HORIZON 2020

EmmA
emotional mobile assistant

Universität Augsburg
Fakultät für Angewandte Informatik

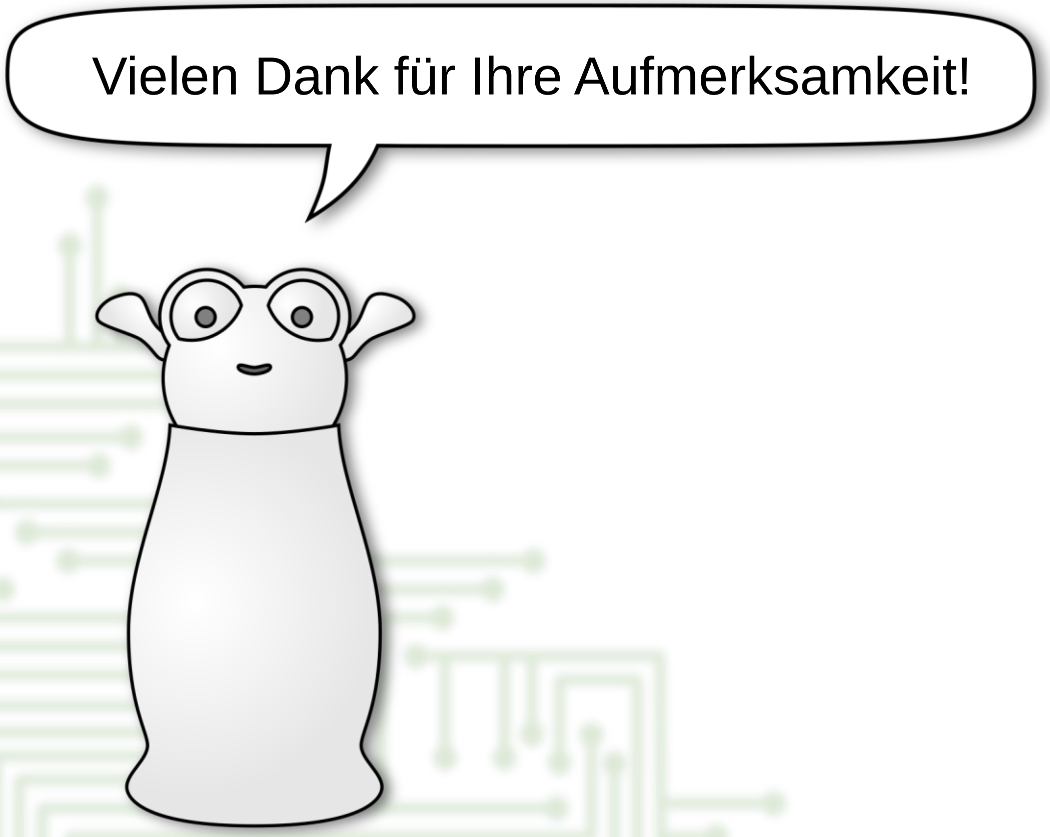

Kathrin Janowski, M.Sc.
Multimodale Mensch-Technik-Interaktion
Universität Augsburg
kathrin.janowski@informatik.uni-augsburg.de
www.uni-augsburg.de

ETH zürich

Detlef Günther, Careum Dialog 2020

Sind soziale Maschinen sozial?

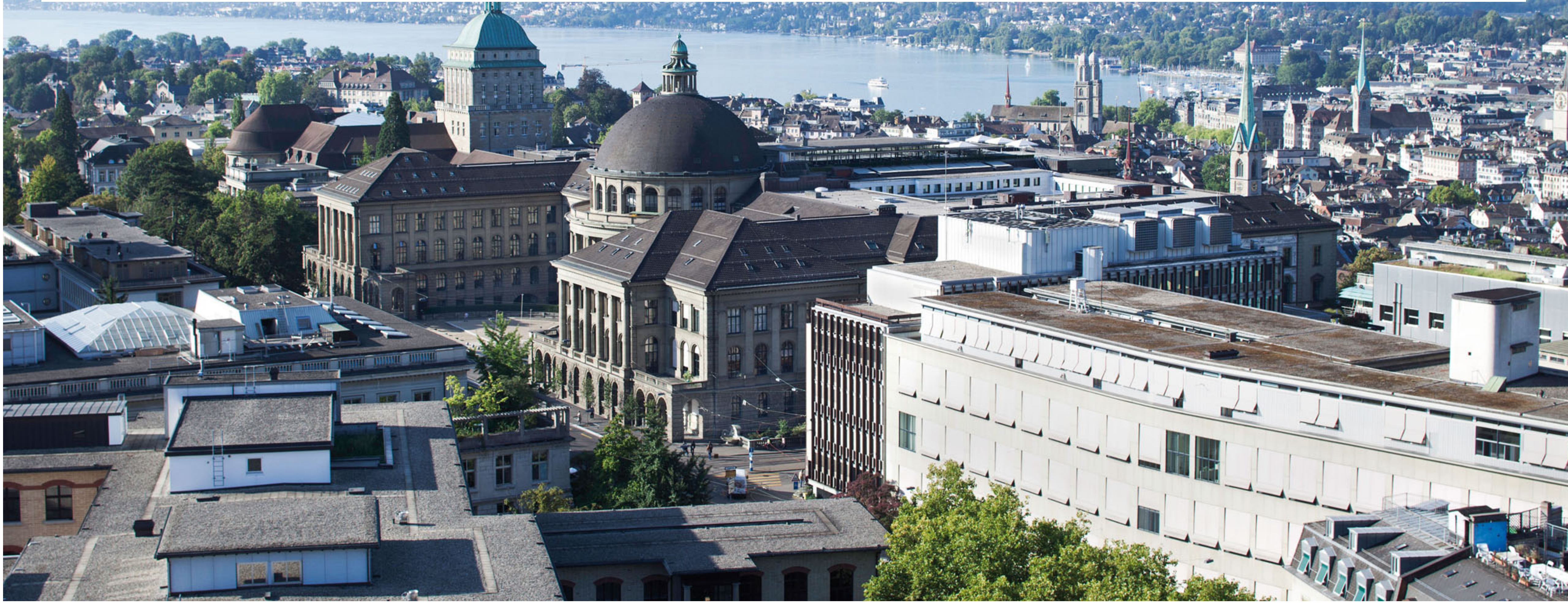

Sind soziale Maschinen sozial?

- Schwindet die Differenz Mensch-Maschine?
- Können Maschinen Partner werden?
- Gefühle erlernen?
- Eine Persönlichkeit entwickeln?
- Kann man ihnen vertrauen?

Was sind soziale Maschinen?

Was sind soziale Maschinen?

Soziale Roboter!

Pflege- oder Service-Roboter!

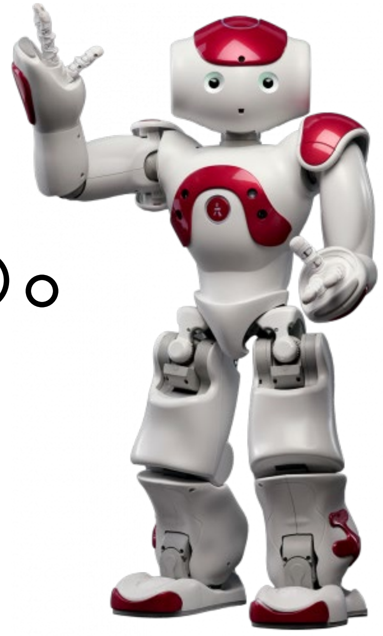

Co-creation! Gemeinsam sind wir klüger

Social Networking!

facebook

Citizen Science!

CAUSE OF HEALTH

CITIZEN SCIENCE CENTER ZURICH

Die Herausforderungen

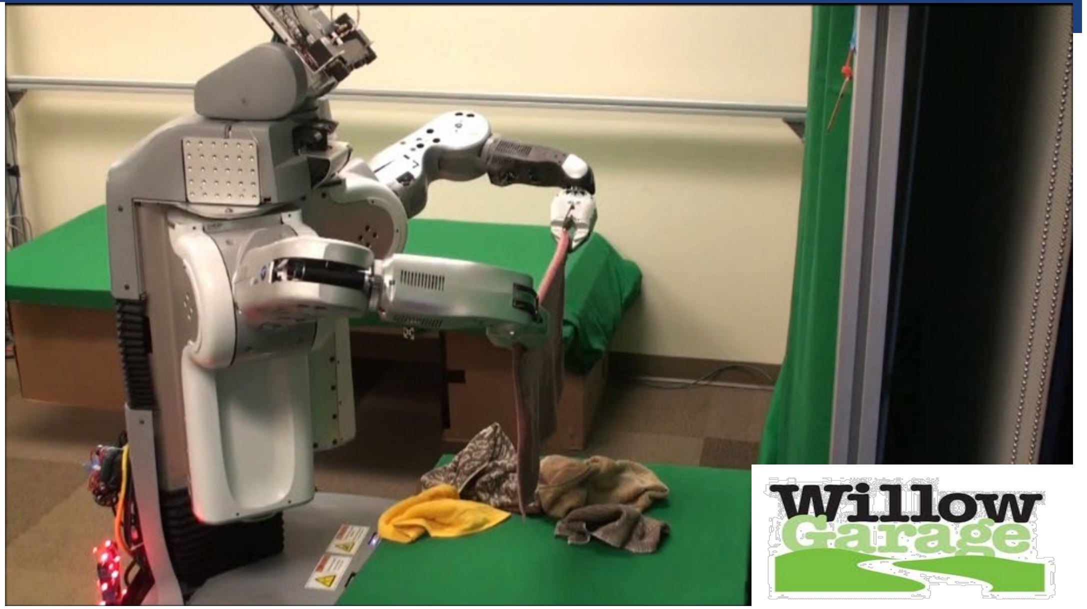

50x speed
https://www.youtube.com/watch?v=gy5g33S0Gzo

Unsere “reale” Welt ist **variantenreich** und sehr **komplex**

- Roboter müssen mit ***unsicherer*** und nur ***teilweise verfügbarer Information*** umgehen können.
- Roboter müssen ***sehen***, ***spüren*** und ***verstehen*** können.
- Roboter müssen ***taktil*** mit der Umgebung interagieren können → («soft robots» mit Kraftreglung)
- Roboter müssen ***intuitiv programmierbar*** sein
- Roboter müssen ***lern-*** und ***anpassungsfähig*** sein

→ Künstliche Intelligenz, Sensoren, Aktoren und Roboterkonzepte

Schwindet die Differenz Mensch-Maschine?

- **Vorstellungsvermögen heute**
  - ➢ Anders als in 2 Jahren!

- scewo – developed by students
- The stair-climbing wheelchair

Spinoff ETH zürich

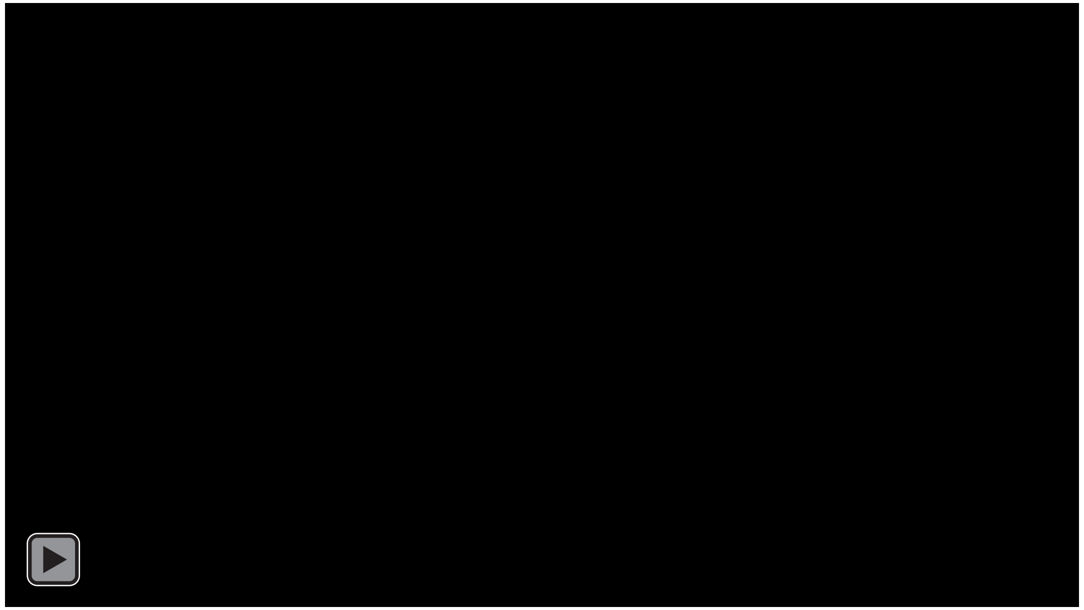

Schwindet die Differenz Mensch-Maschine?

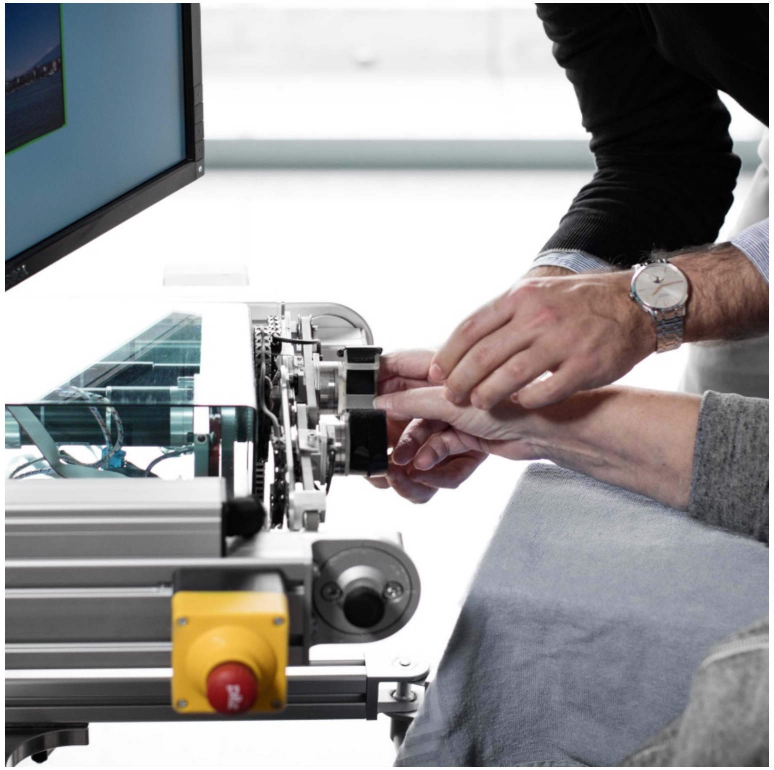

Roboter-unterstützte Rehabilitation der Handfunktion

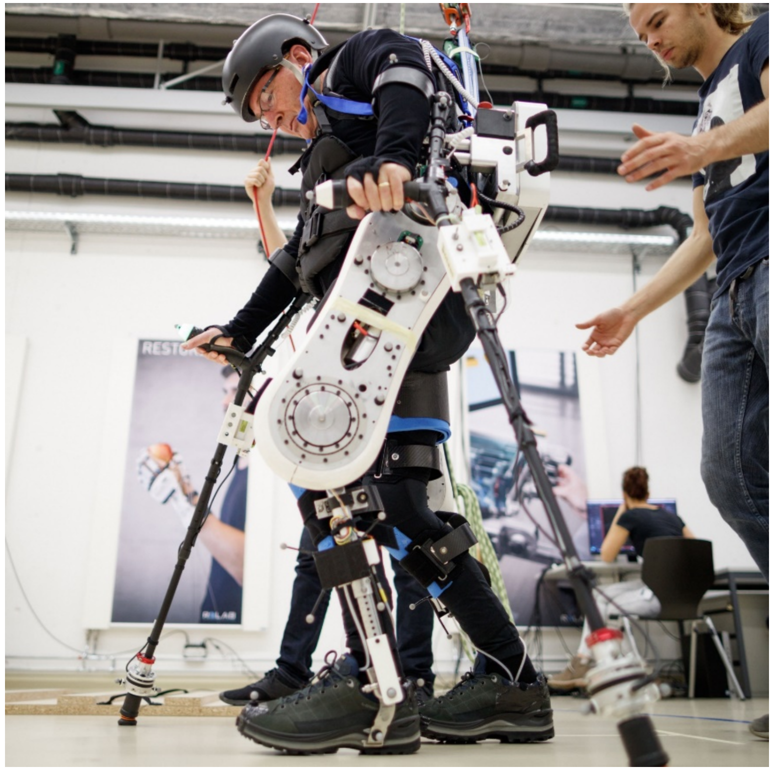

Ein Exoskelett für Querschnittsgelähmte

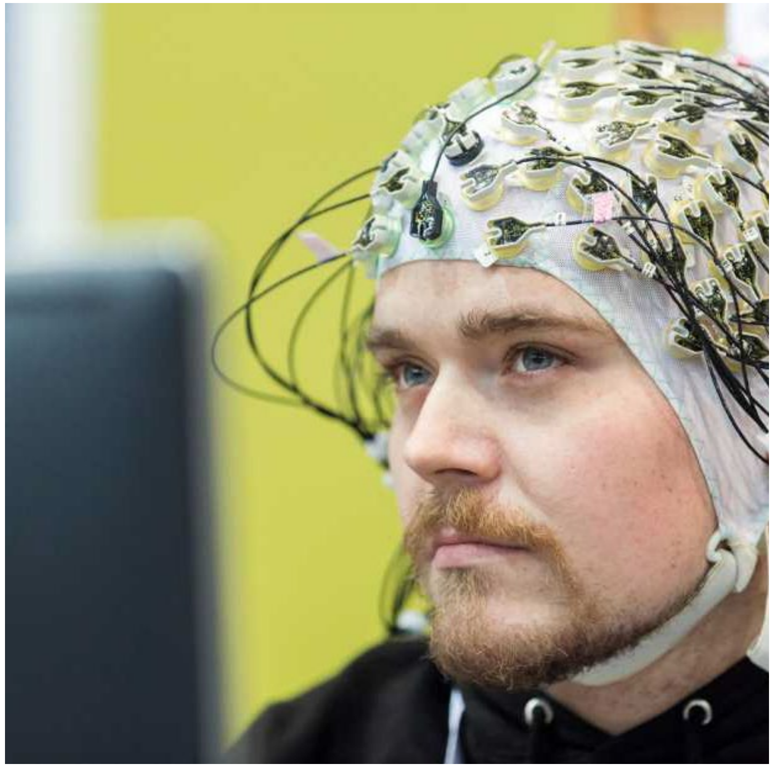

Gehirn-Computer-Schnittstelle
➢ Mit Kraft der Gedanken

Projekte Prof. Robert Gassert, ETH Zürich

Künstliche Intelligenz und Maschinelles Lernen – Ja aber

- Grosse Datenmengen für das Lernen
- Grosse Rechenleistung

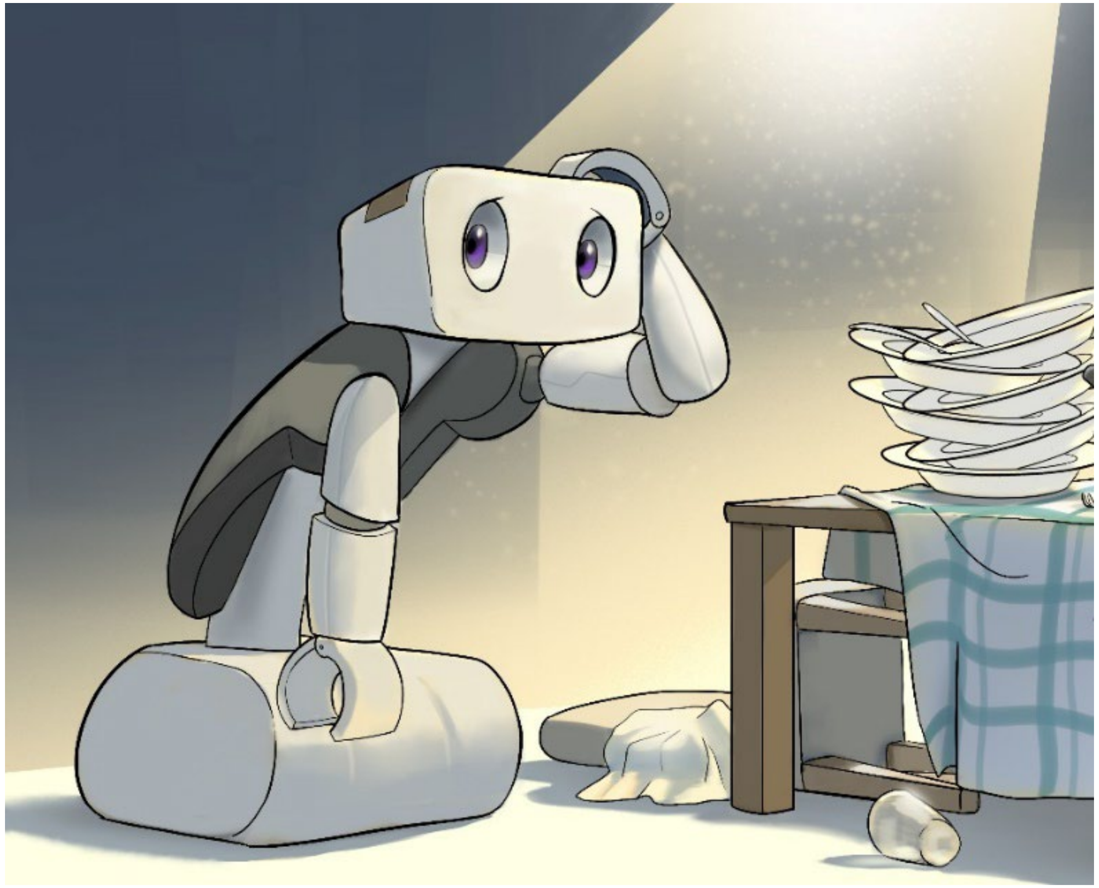

Generalisierung?

- Lernen über Interaktion mit der Welt?
- Multimodales Lernen?
- Lernen von semantischer Information
- Verstehen

Künstliche Intelligenz und Maschinelles Lernen

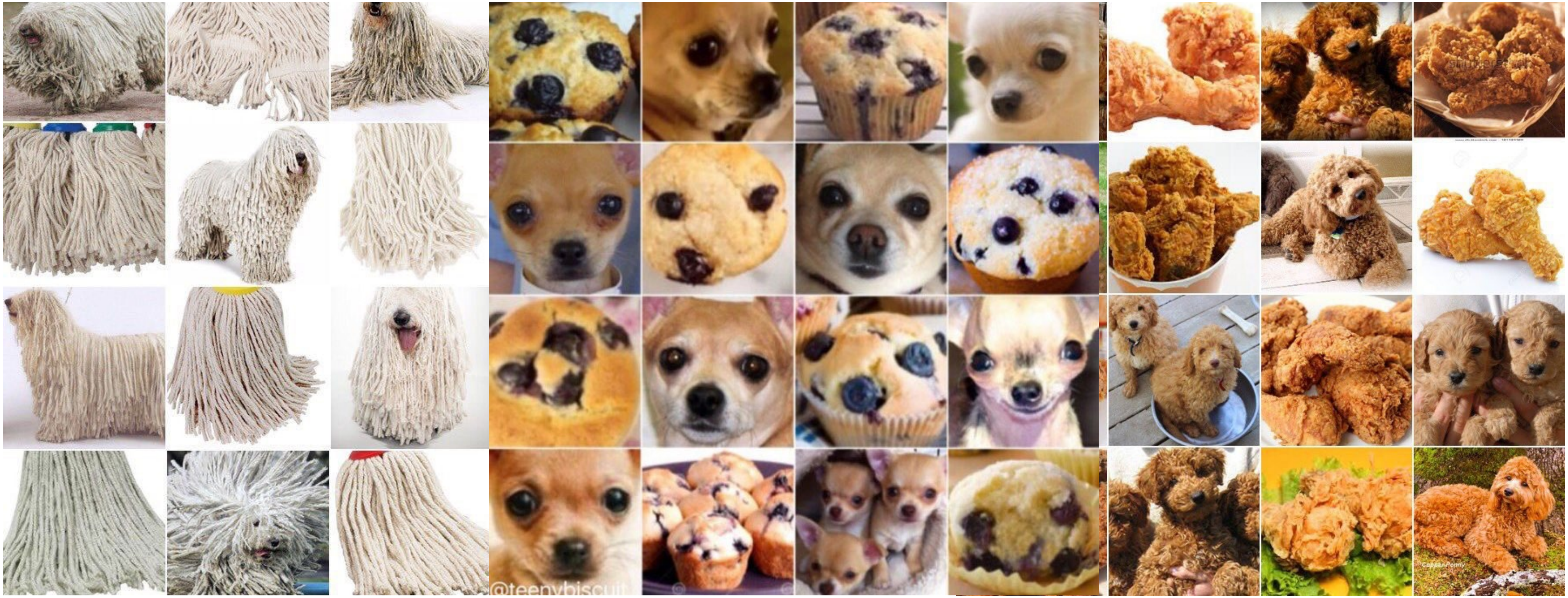

Herausforderung

- Menschen sind nicht zu schlagen wenn es darum geht
  komplexe Entscheidungen zu treffen und mit anderen zu interagieren.

- Computer sind viel besser um
  einfache aber schnelle Entscheidungen zu treffen.

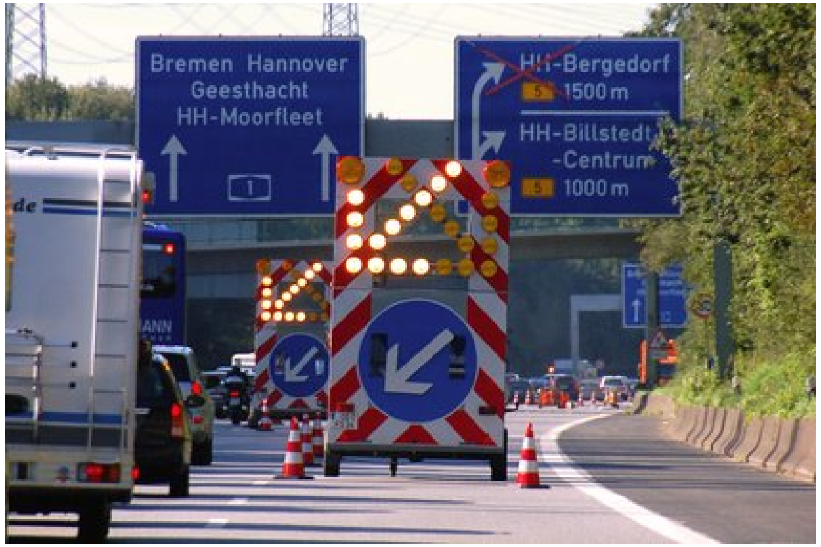

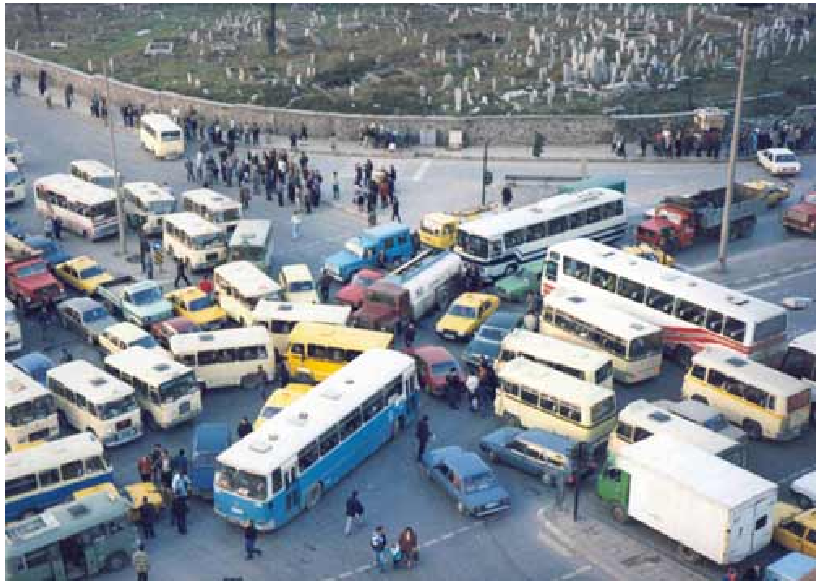

Können Maschinen Partner werden?

- Virtuelle und reale Welt vereinen

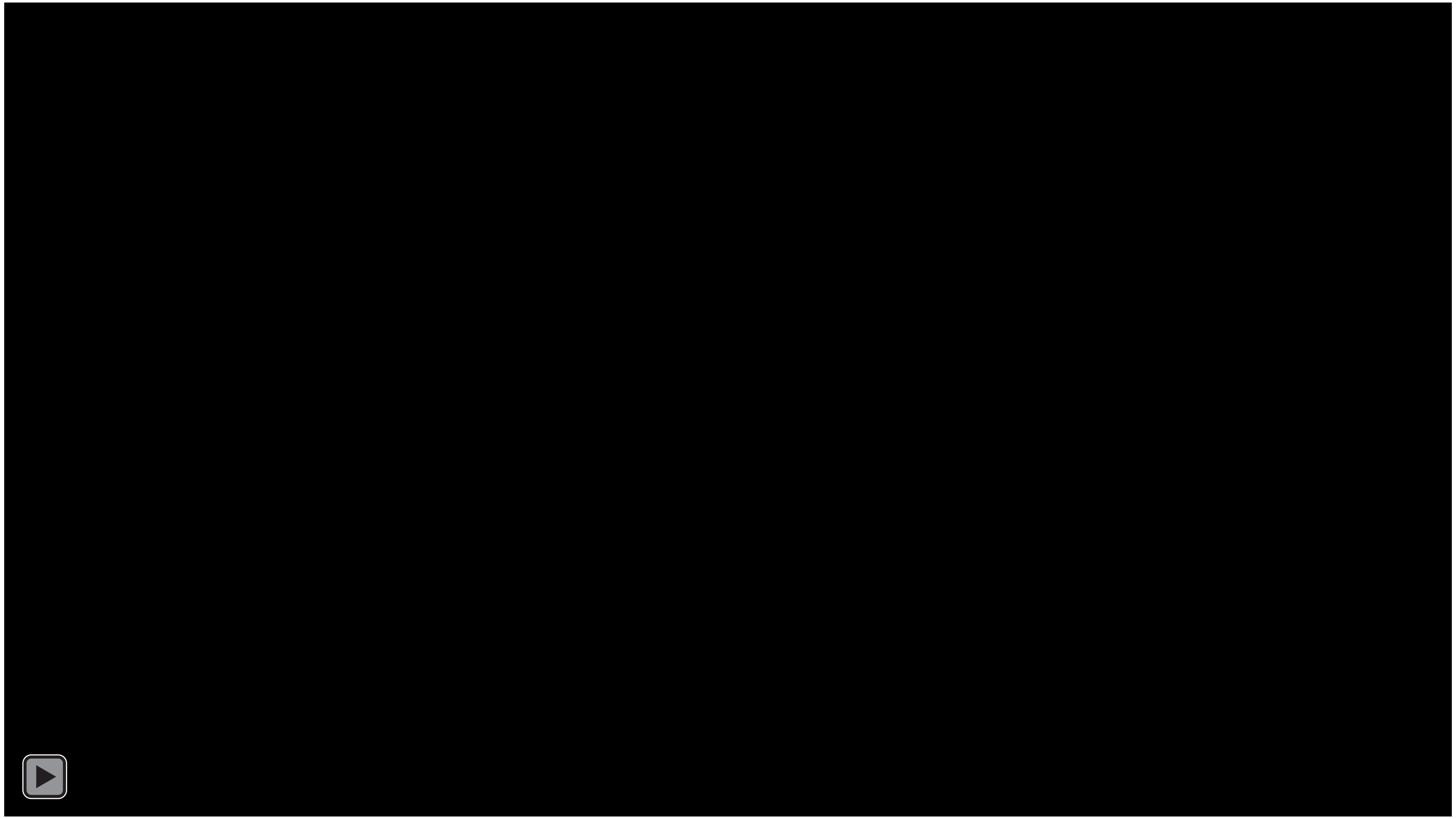

Projekt Prof. Roland Siegwart

Google

Project Tango

sevensense

Spinoff ETH zürich

Können Maschinen Partner werden?

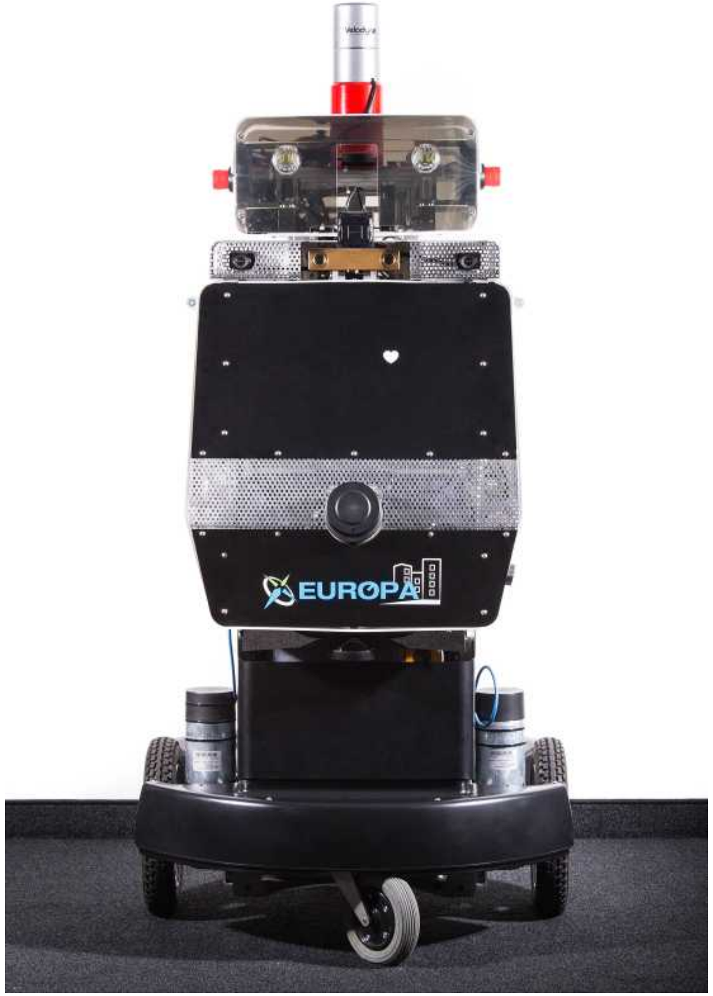

EUROPA | *European Robotic Pedestrian Assistant*

Serviceroboter, bewegt sich autonom in städtischen Umgebungen

ETH Zürich in Zusammenarbeit mit:

- University of Freiburg,
- Univ. of Oxford
- KU Leuven
- RWTH Aachen
- BlueBotics

Können Maschinen Gefühle erlernen?

Beinprothese mit Berührungs-Empfinden erleichtert das Gehen.

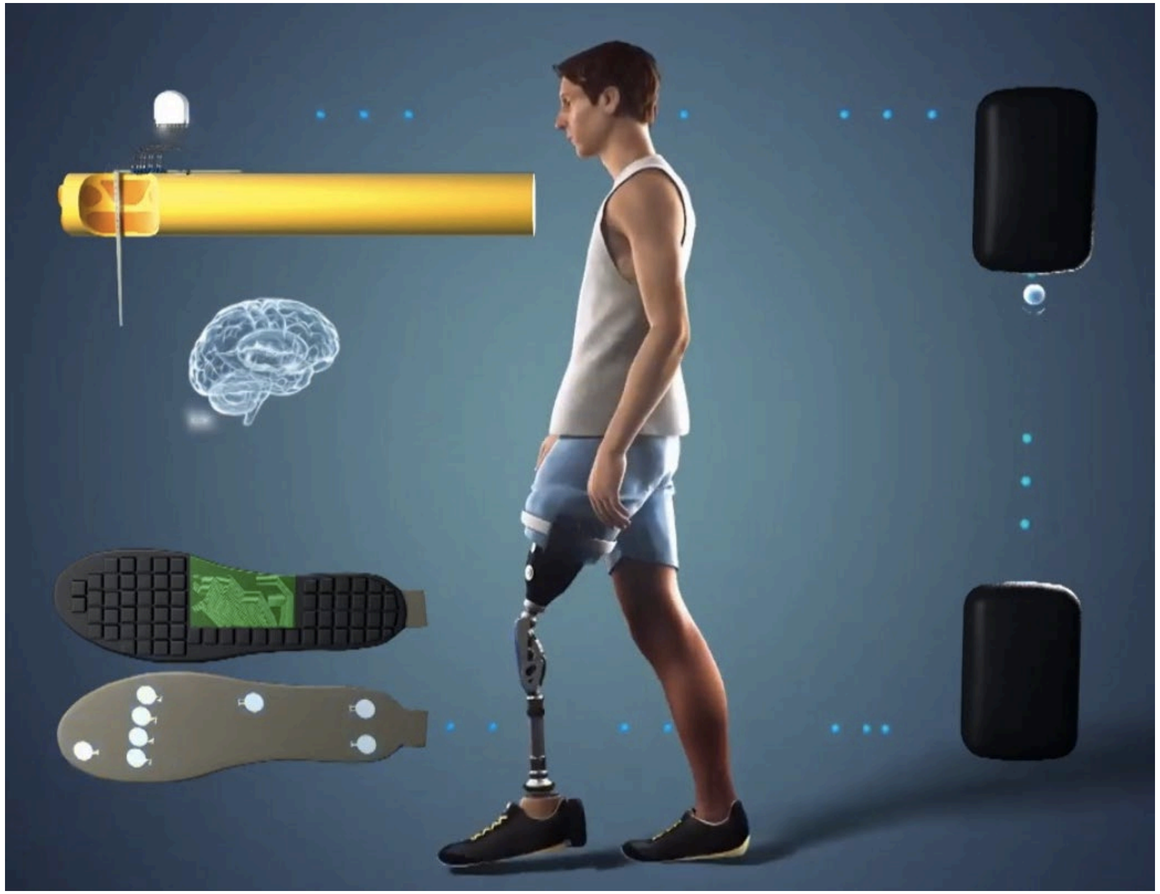

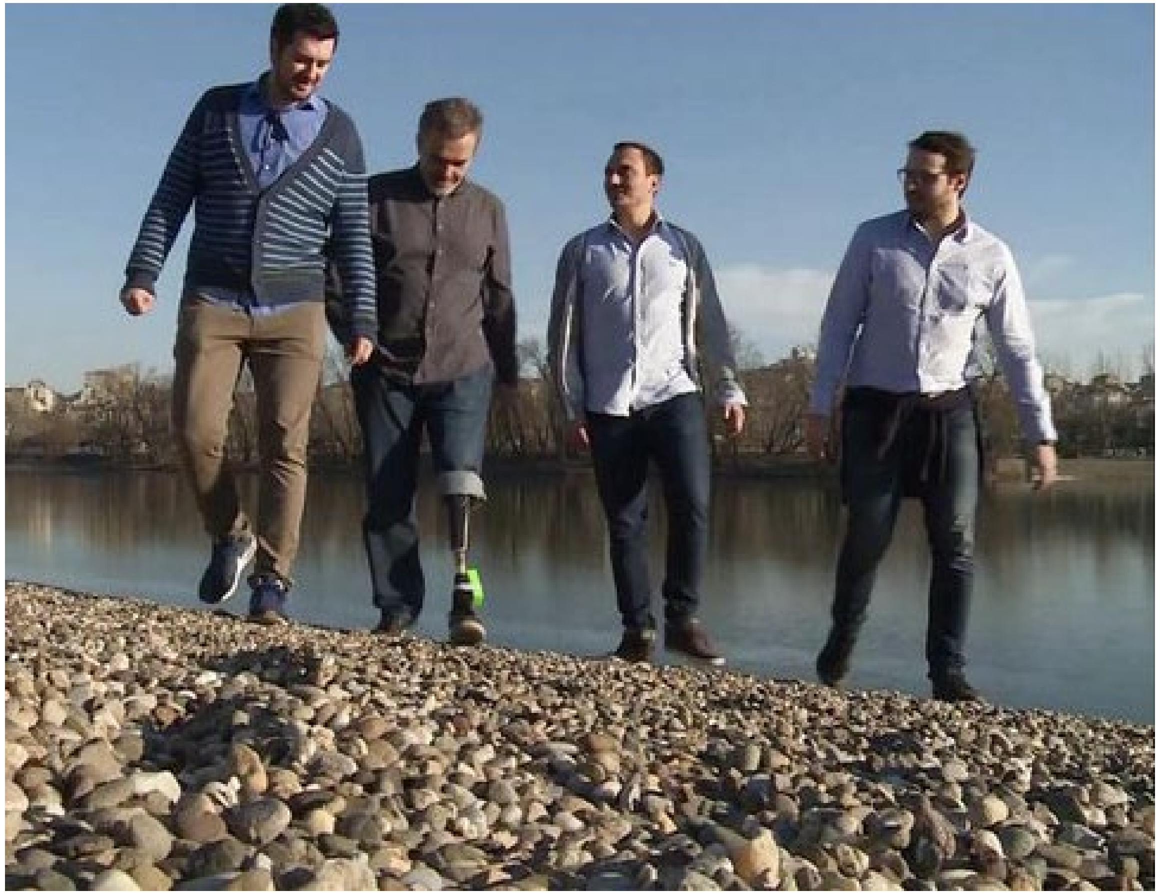

Prof. Stanisa Raspopovic, ETH Zürich

Können Maschinen eine Persönlichkeit entwickeln? Kann man ihnen vertrauen?

Künstliche Intelligenz und Robotik

- Lösungen für ein breites Spektrum von Problemen der Gesellschaft.
- Diskussion über ihren Einsatz, Nutzung und die ethische Dimension ist wichtig.

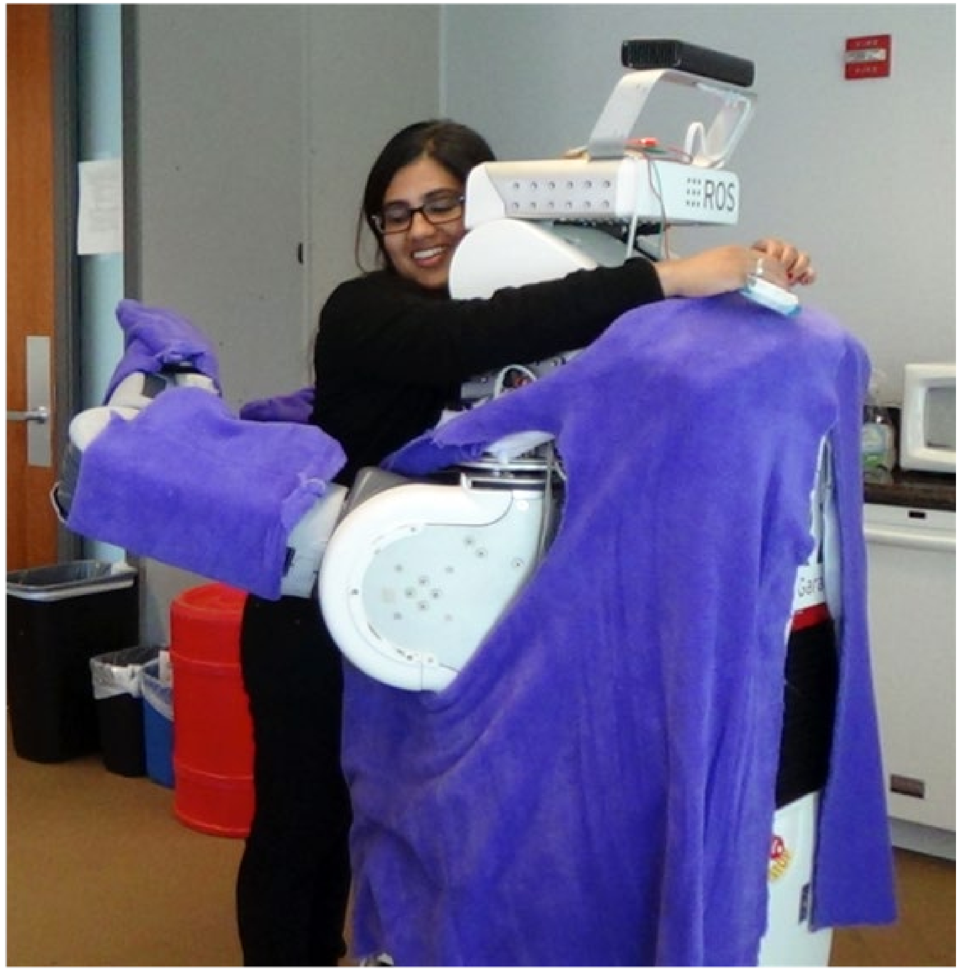

HuggieBot
Dr. Alexis Block, ETH Zürich

ETH zürich

Thank you for your attention!

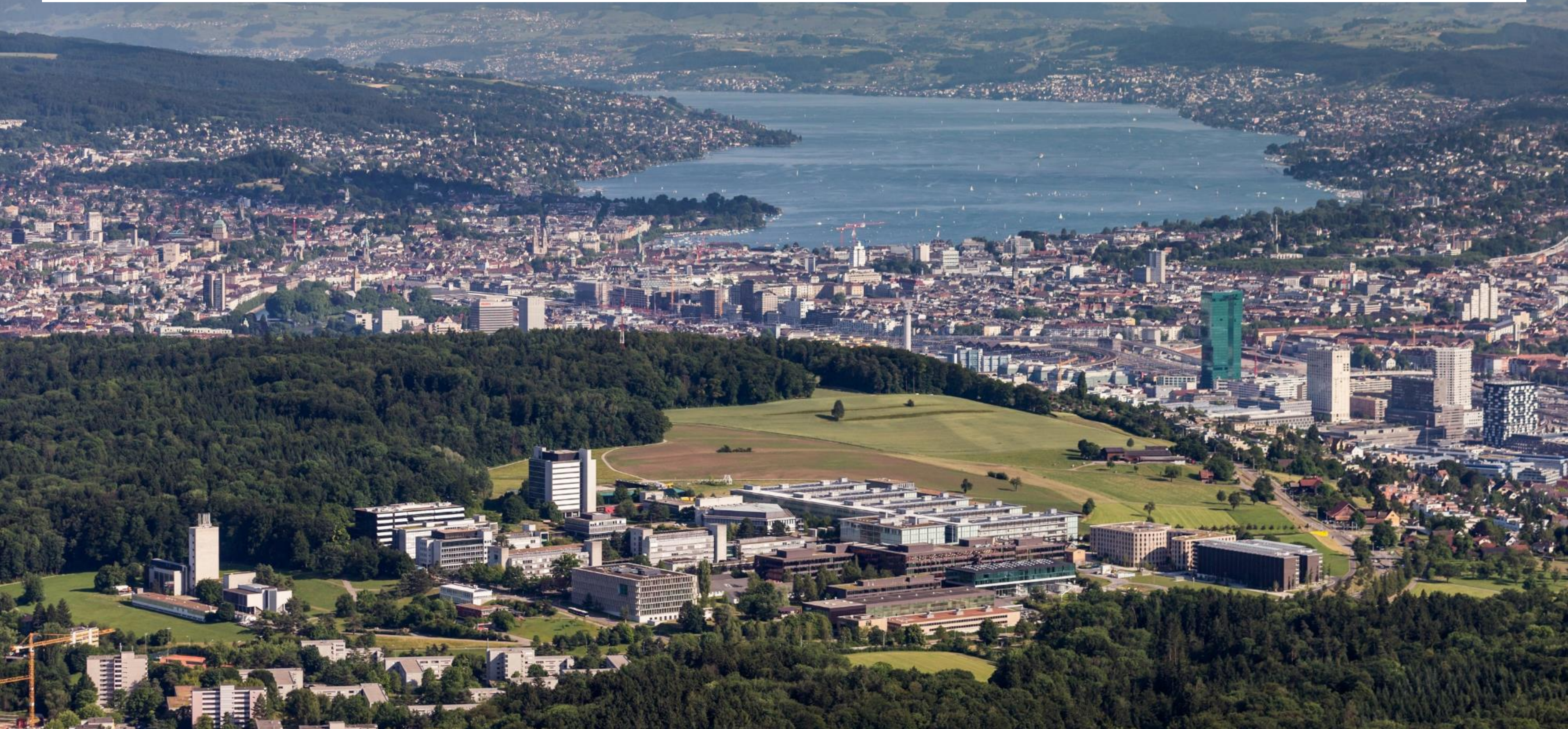

ETH zürich

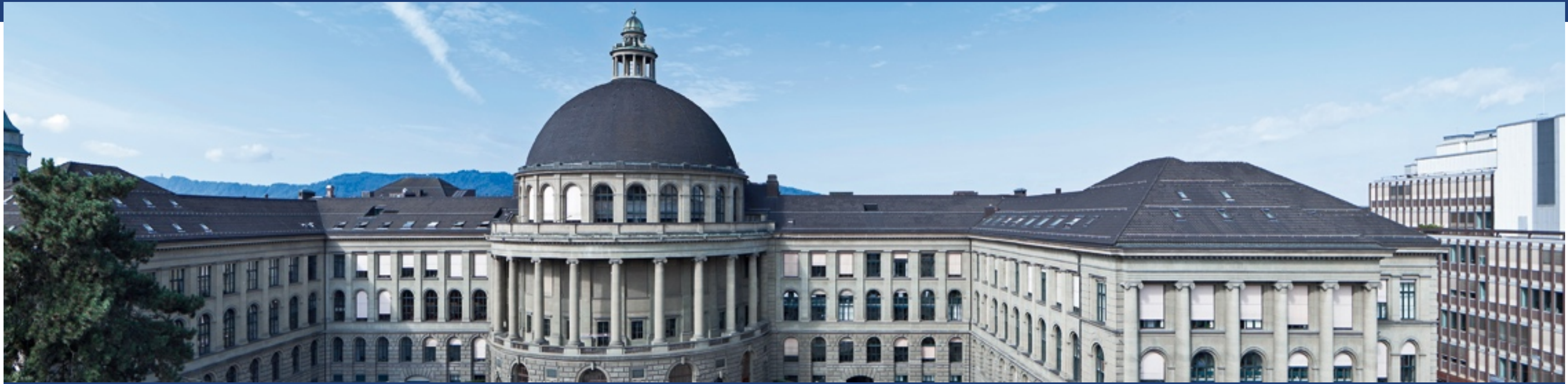

Social Robots in Health Care- ethical challenges

Prof. Effy Vayena

Health Ethics and Policy Lab
effy.vayena@hest.ethz.ch
@EffyVayena

Health Ethics & Policy Lab

Outline

- AI and Robots
- Hype and hope

- Autonomy: consent and patient choice
- Human relationships
- Privacy
- Trust
- Disparities

Figure 2. Sectors leading in AI adoption today

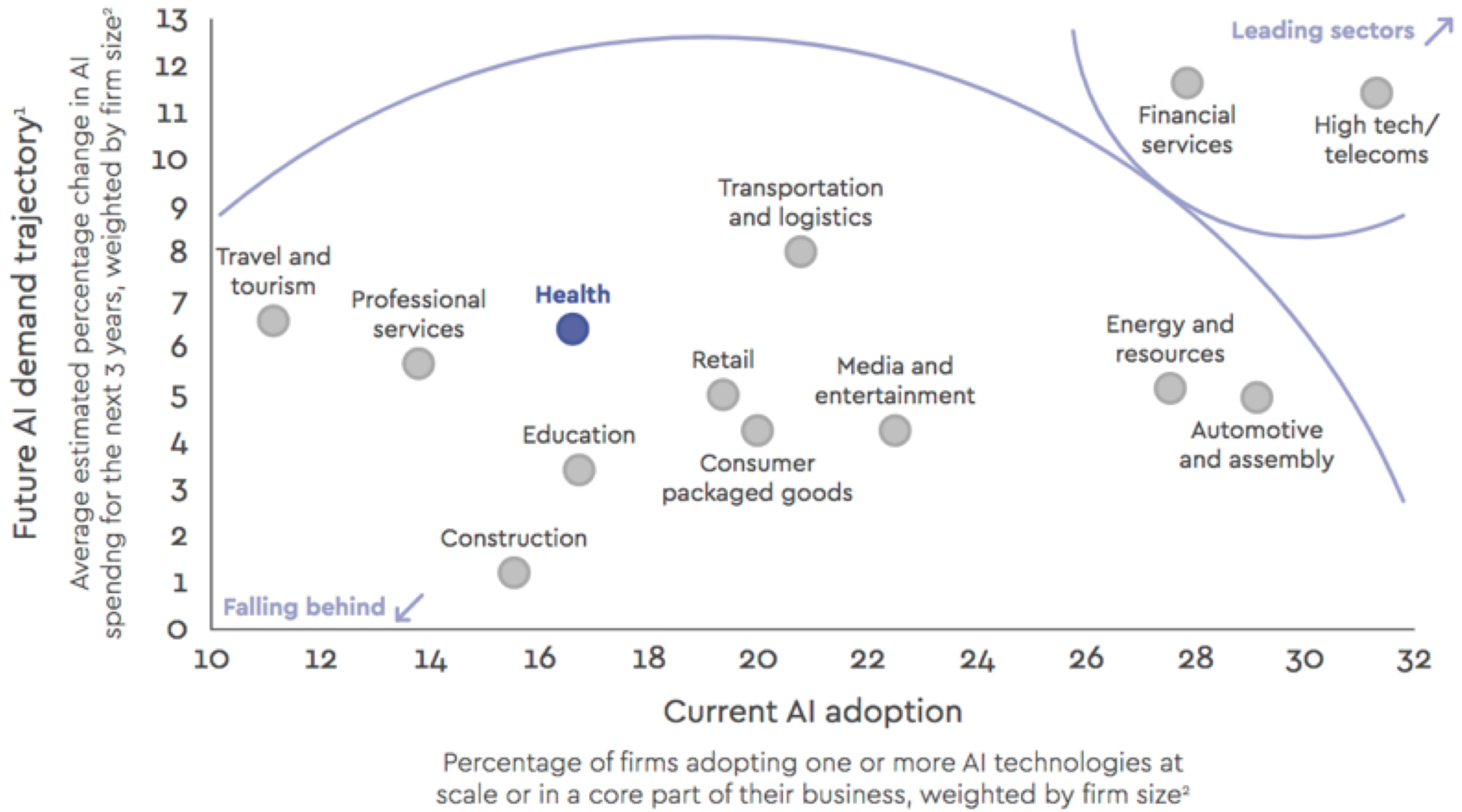

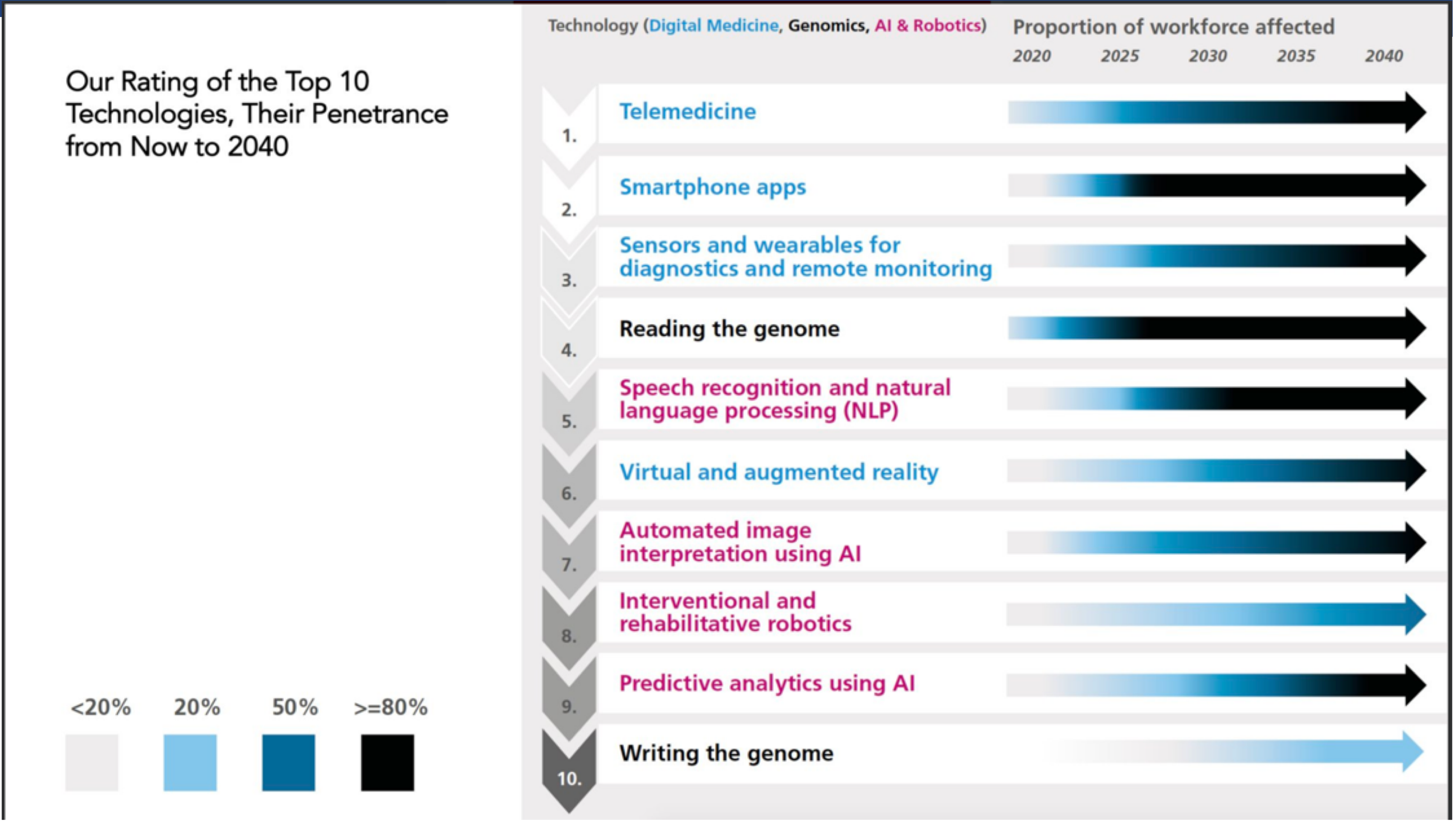
Our Rating of the Top 10 Technologies, Their Penetrance from Now to 2040
<20%
20%
50%
>=80%
Technology (Digital Medicine, Genomics, AI & Robotics)
Proportion of workforce affected
2020
2025
2030
2035
2040
1. Telemedicine
2. Smartphone apps
3. Sensors and wearables for diagnostics and remote monitoring
4. Reading the genome
5. Speech recognition and natural language processing (NLP)
6. Virtual and augmented reality
7. Automated image interpretation using AI
8. Interventional and rehabilitative robotics
9. Predictive analytics using AI
10. Writing the genome

Different types

Robotic surgery allowing more accurate, less invasive and remote interventions relying on the availability and assessment of vast amounts of data;

Care and socially assistive robots allowing to meet the expanding demands for long-term care from an ageing population affected by multi-morbidities;

Rehabilitation systems supporting the recovery of patients as well as their long-term treatment at home rather than at a healthcare facility;

Training for health and care workers offering support for continuous training and life-long learning initiatives.

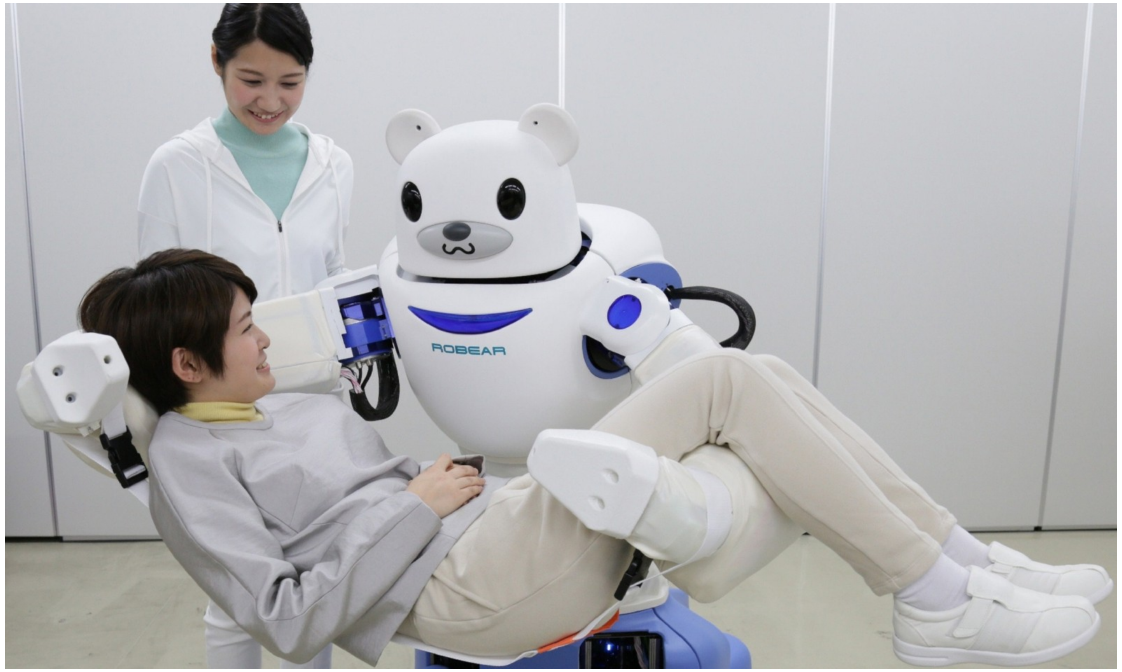

“Autonomy” and control

- Autonomous robots
  - How much?
- Who controls what?
  - Patient
  - Health care professional
- Personal autonomy
  - Consent and deferring agency

FUTURE ADVOCACY

ARTIFICIAL INTELLIGENCE: YouGov Poll 2017

AI should/shouldn't be used for...

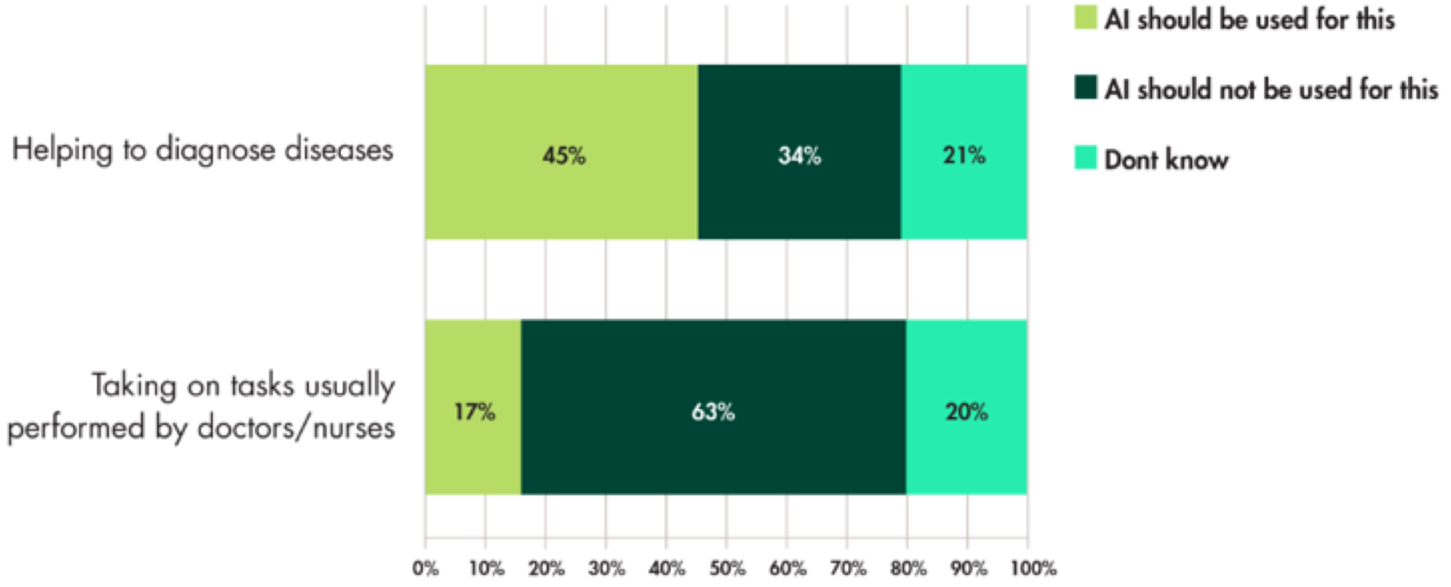

Many British people support the use of AI to help diagnose disease, but most don't think AI should be used for other tasks usually performed by doctors and nurses such as suggesting treatment

Total sample size was 2108 adults. Fieldwork was undertaken between 29th September - 2nd October 2017. The survey was carried out online. The figures have been weighted and are representative of all UK adults (aged 18+).

Human relationships

- Dehumanizing (human-like)
- Objectifying
- Deception (bonding)
- Human-to-human interaction and moral boundaries
- Therapeutic relationships

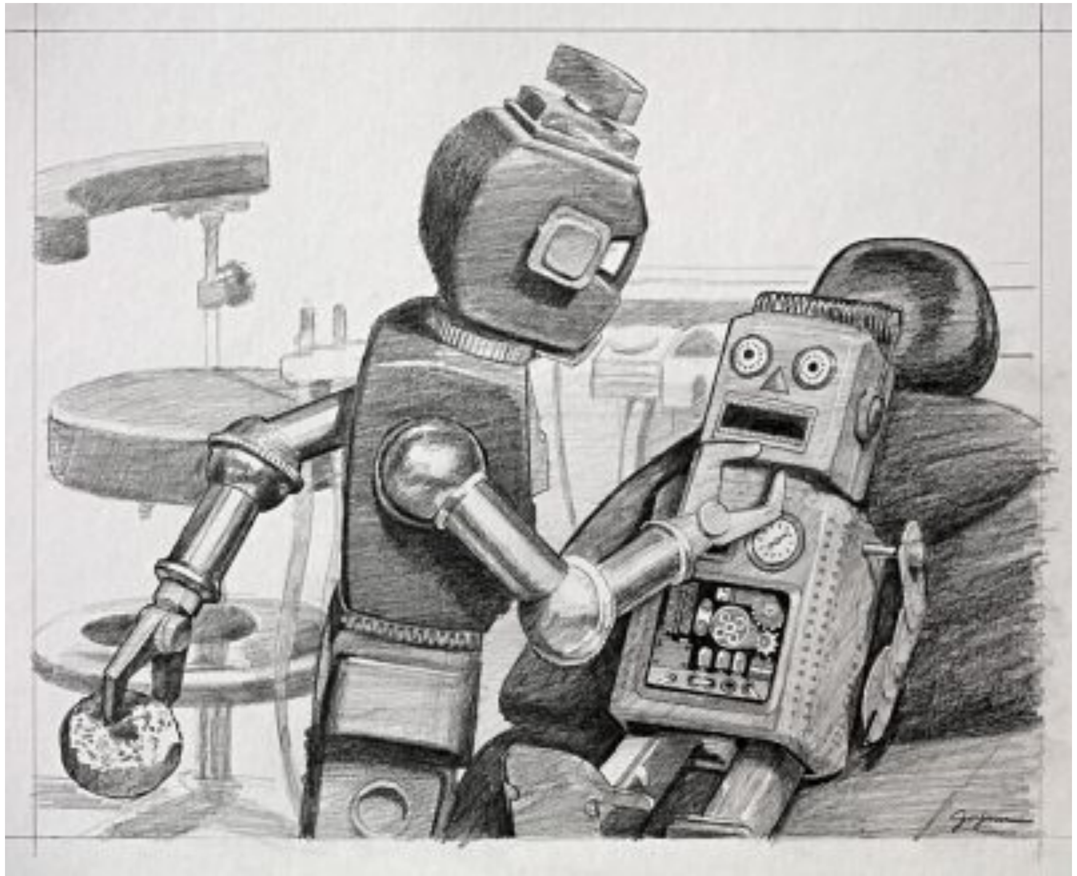

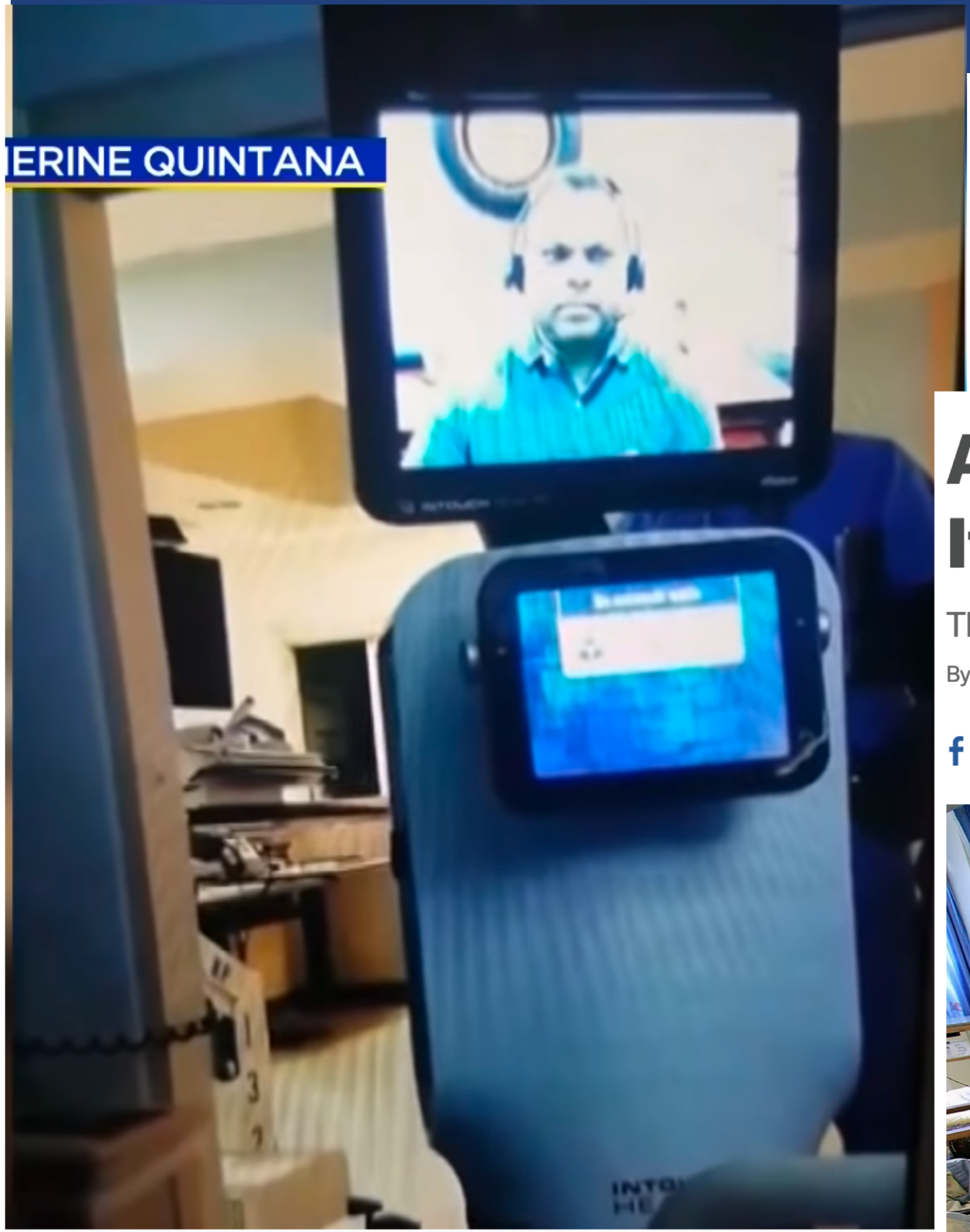

A "robot" doctor told a patient he was dying. It might not be the last time.

This is the future of care for many patients with limited access.

By Haider Warraich | Mar 13, 2019, 12:30pm EDT

SHARE

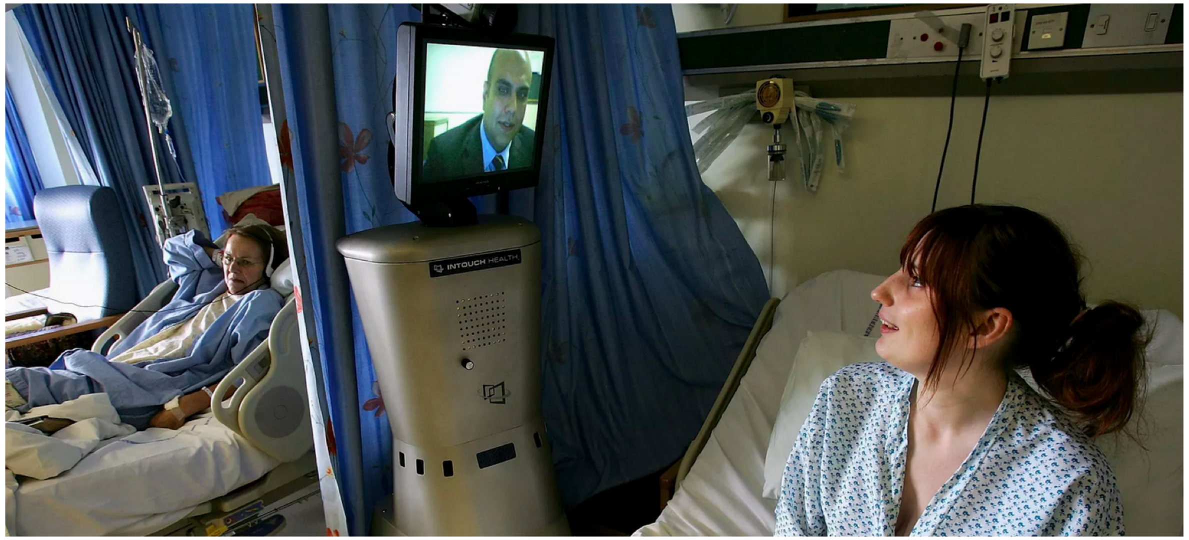

News > World > Americas

Robot rolls into hospital ward and tells 97-year-old man he is dying

Family of Ernesto Quintana say terminal diagnosis should be given by 'a human being and not a machine'

Emma Snaith | Saturday 9 March 2019 19:15 | 16 comments

Privacy

- Data collection
- Analytics and predictions
- Information flow

Informational privacy

- Physical presence & autonomy

Physical privacy

- Human-like robots
- Privacy of bystanders
- Connected systems

"Social privacy"

effy.vayena@hest.ethz.ch

Trust

- Transparency
  - Reliability
  - Over- trust
- Bias
- Accountability
- Liability

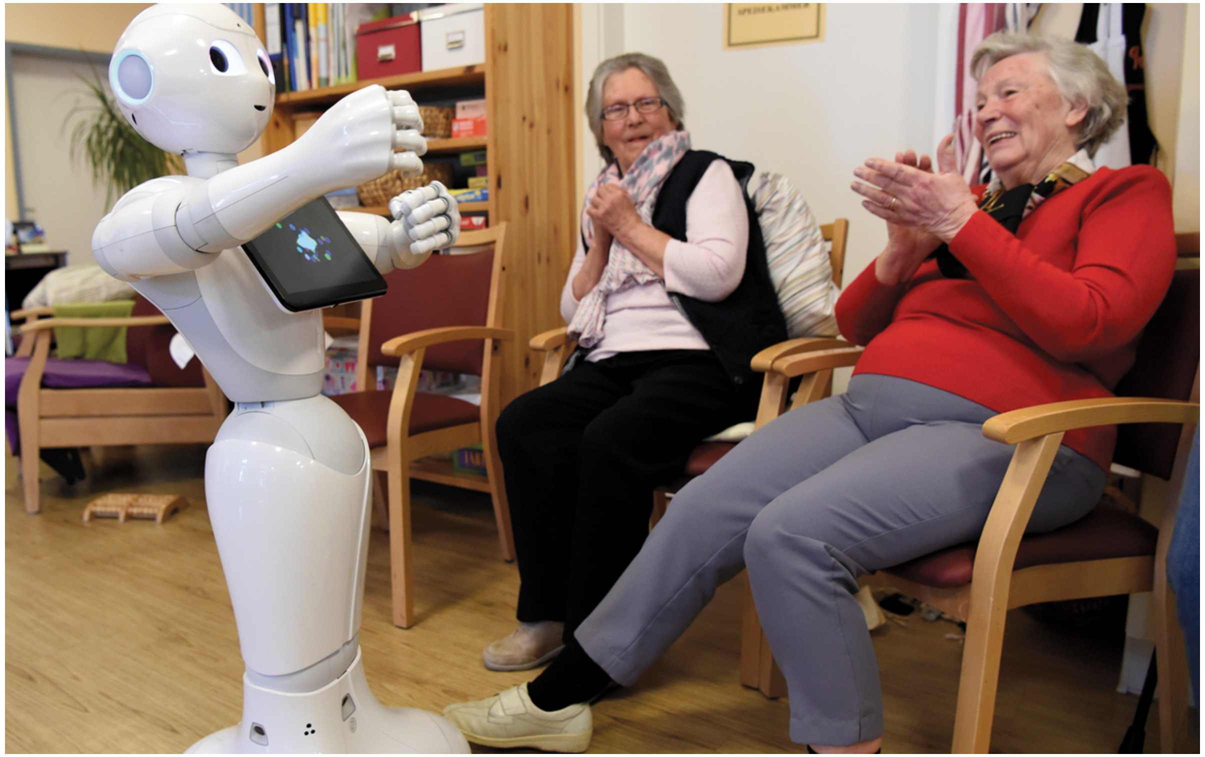

Disparities

- Discrimination
- Access
- Fairness
- Distribution of benefit

"My fear is we will end up with what I've been calling a 'health care apartheid". If we create algorithmic care and 'kiosk' it in some fashion — focusing on efficiency and throughput — the people who will end up having access and using it will be the ones who already lack privileges of various kinds"
Sonoo Thadaney Israni, Stanford University

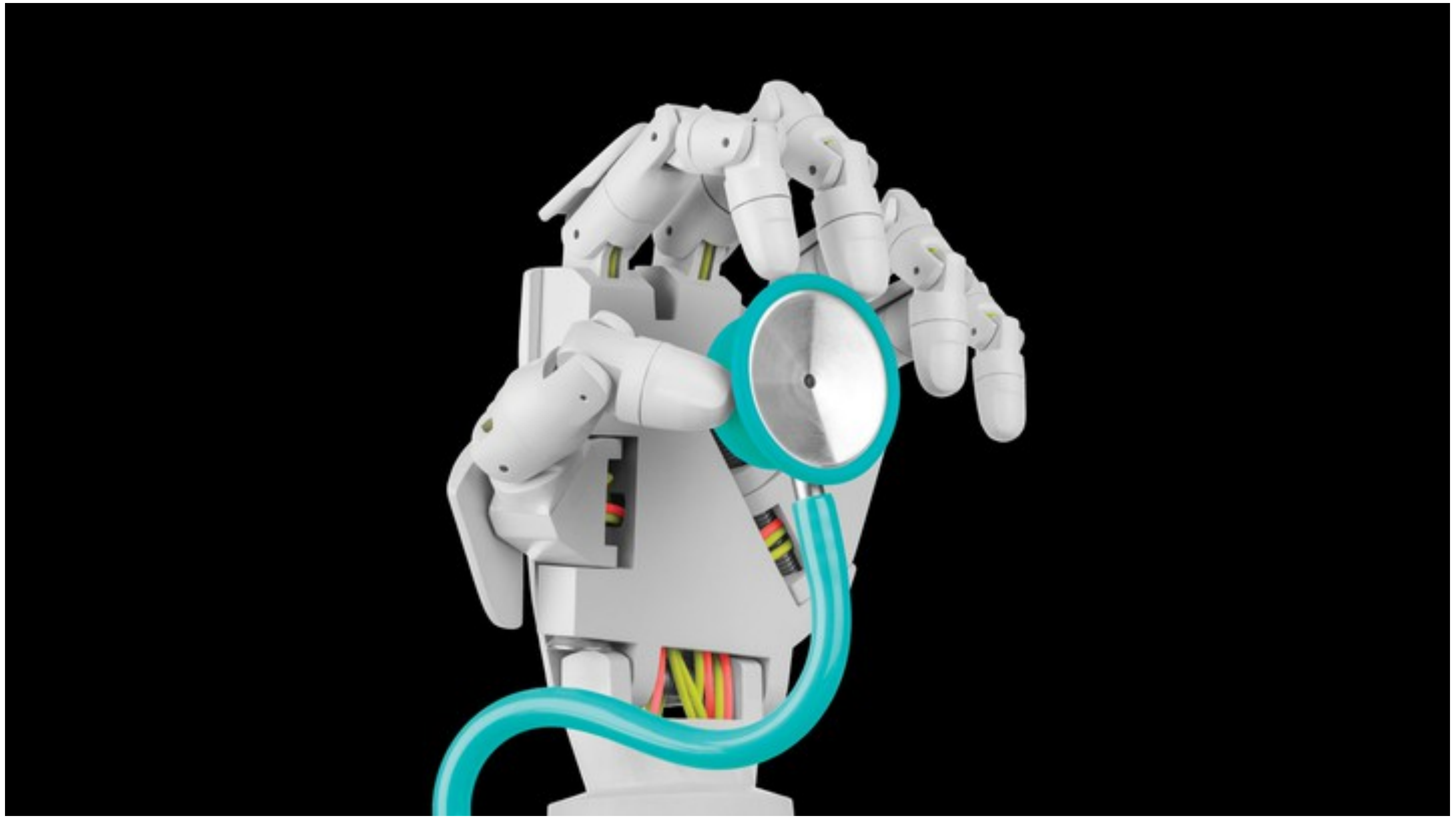

“Where is everyone? What are they doing? Hello! It’s my body, you know! “Vergehes, NYT magazine 2018

ETH zürich

THANK YOU!!

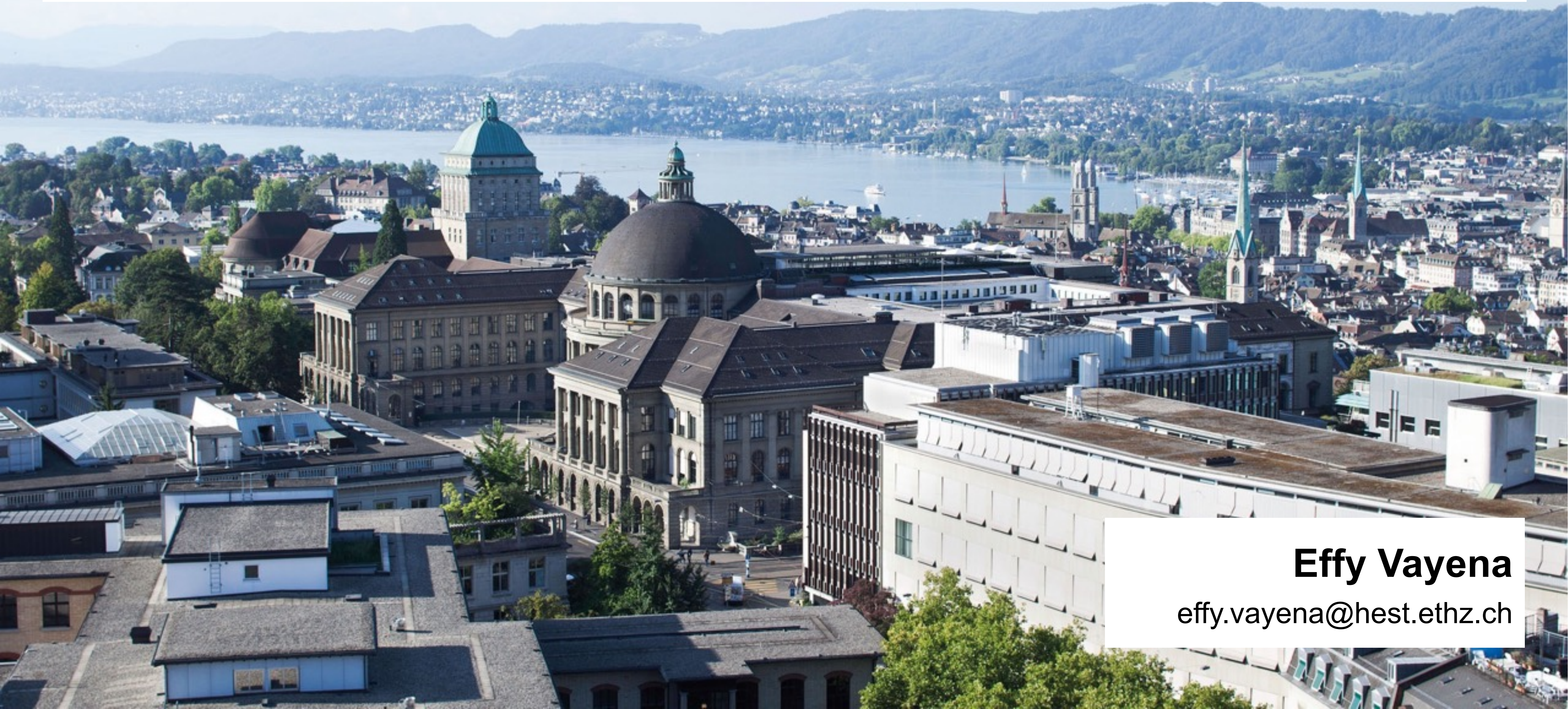

Effy Vayena
effy.vayena@hest.ethz.ch

Faculty of Health – Medicine Department
Didactics & Educational Research in Healthcare
UNI – WH.DE/
universität Witten/Herdecke

Pflegeszenarien in einer digitalen Welt

Auf dem Weg in eine Utopie oder Dystopie?

@medizindidaktik
zweikörperproblem
@medizindidaktik
Didaktik und Bildungsforschung
@JanP_Ehlers
@medizindidaktik

Digitale Transformation

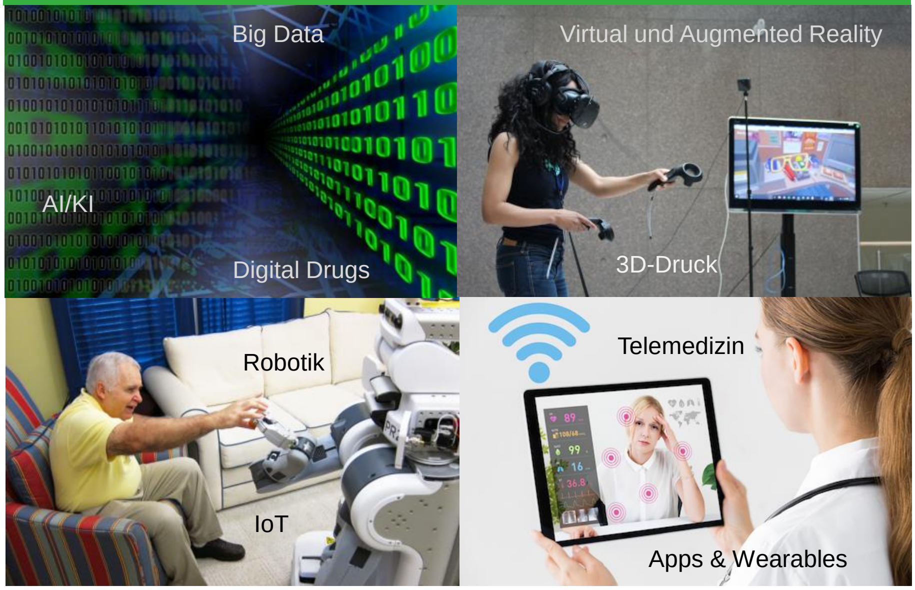

Digitale Transformation

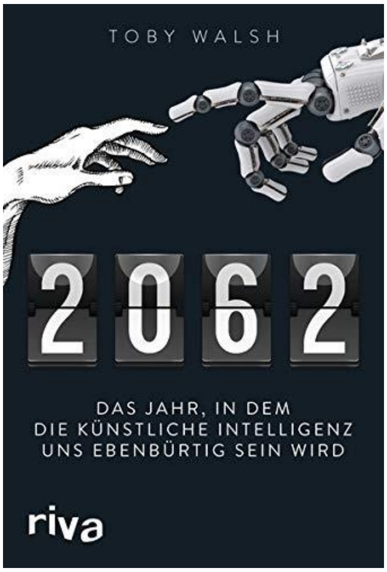

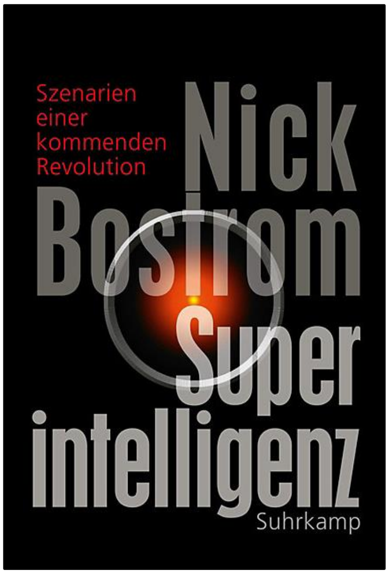

KLUG P (2018):The Debate about Responsible Artificial Intelligence in the European Union: Utopian or Dystopian? BA ThesisFaculty of Behavioral, Management and Social Science, University of Twente

Vorstellungen der Zukunft

Utopie

Fiktive Gesellschaftsordnung, meist positiv.

- **Frieden und Harmonie**
- Lebensverbesserung
- Revitalisierung der Politik
- Kontrolle von Natur und Technologie
- Würde, Privatsphäre
- Unparteilichkeit, Gleichheit
- Freiheit, Autonomie
- Selbstbestimmung
- Persönliche Erfüllung
- Fortschritt
- Individuelle und gesellschaftliche Erlösung
- Umwelterhaltung

Dystopie

Gesellschaft, die sich zum Negativen entwickelt

- **Krieg und Chaos**
- Armut
- Macht und Kontrolle
- Verlust der Kontrolle über die Technologie
- Unterdrückung
- Massenüberwachung
- Soziale und wirtschaftliche Ungleichheiten
- Dominanz, Diskriminierung
- Unterwerfung, Sklaverei
- Umweltzerstörung
- Aufhebung der Rechte

Zukunft der Gesundheitsversorgung

Utopie

- Miteinander von Mensch und Technik
- Antworten auf Pflegekräftemangel und Überalterung
- Menschen konzentriert auf soziale Interaktion
- Geringere Fehleranfälligkeit
- Aufbau einer neuen, friedlichen Gesellschaftsform
- **„Alles wird gut!“**

Dystopie

- Überwachung
- Entmenschlichung
- Entsolidarisierung/ Zweiklassenpflege
- Internationale Datenkraken
- Energieverbrauch / Klimakatastrophe
- Arbeits- und Nutzlosigkeit von Menschen
- **„Wir haben es ja schon immer geahnt!“**

Foresight-Studie „Digitale Arbeitswelt“ (2016)
für das Bundesministerium für Arbeit und Soziales

Technisierung der Pflege

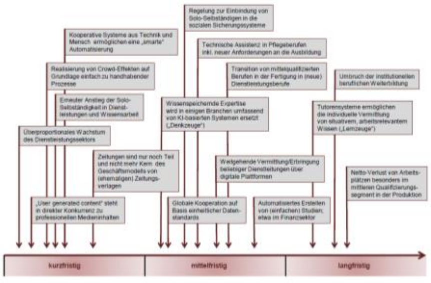

Abb. 1 Meilensteine in der Entwicklung der digitalen Arbeit auf Basis einer umfassenden und konsolidierten Analyse für die Branchen Produktion, Dienstleistungen und Medien

„Zeitgleich werden für standardisierbare, manuelle Arbeiten zunehmend ***robotische Lösungen*** *erwartet. Analog zur Produktion werden* ***assistive Systeme*** *mittelfristig in Dienstleistungsbereichen zur Verfügung stehen, die bisher durch einen vergleichsweise niedrigen Technisierungsgrad gekennzeichnet waren, so etwa in der Reinigung oder der* ***Altenpflege****.“*

„Unbestritten ist jedoch, dass die Technisierung der Pflege zu einer zusätzlichen *Qualifizierung* der in diesem Bereich Tätigen führen wird, die mit einer gewissen Wahrscheinlichkeit auch in einer **allgemeinen Aufwertung**, beispielsweise des Pflegeberufs, und der damit verbundenen **Entlohnung** Ausdruck finden wird.“

European Health Forum (2017)
The EHFG Health Future Project

EHF: Scenarios for health in 2037

Scenario 1: The Future is Local
Lokale Verantwortung für Gesunderhaltung mit Risiko der eingeschränkten Freiheiten.

Scenario 2: Your Health, Your Responsibility
Selbstverantwortung mit Belohnungen aber Stigmatisierung und beschräktem Zugang zu Unterstützung.

Scenario 3: Technology Delivers
Staatliche Technologiestrategie mit Gentests, BigData und HighTech Therapien, Patien*innen werden Kund*innen.

https://www.ehfg.org/fileadmin/downloads/13-0-health-futures/EHFG_Health_Futures_Project_Report.pdf

LEHOUX P and GRIMARD D (2018): When robots care: Public deliberations on how technology and humans may support independent living for older adults. Social Science & Medicine 211, 330-337

Unterstützung im Alter

Roboter	Menschen
**Monitoring und Sicherheit**	
24/7 verfügbar, Datenspeicherung, mobile Assistenz	Physische Unversehrtheit, Anpassungs-fähigkeit, Kontext-Erfahrungen
**Aktivitäten des täglichen Lebens**	
Erinnerung an Medikamente, Unterstützung beim Anziehen/Hygiene	Wichtiger menschlicher Kontakt, Respekt der menschlichen Würde
Fehleranfälligkeit, Kosten, Kontrollver-lust, negative Gesundheitsauswirkung	Abhängigkeit von (schlechten) Menschen, geteilte Aufmerksamkeit
**Soziale Aktivitäten & emotionale Stütze**	
Zeit für soziale Aktivitäten, Kommu-nikation mit Angehörigen, Emotionen +/-	Freundlichkeit & Wärme, Erfahrungs-austausch, Vertrauen, Beziehung
Entmenschlichung, Automatisierung, Isolation, Mobilitätsverlust	

Spannungsfeld

„Robotic Care“	„Analog Care“
„Smart Care“	„Deep Care“

1. „Robotic Care“

Hauptmerkmal:	Ersetzen menschlicher Pflege durch Technologie
Treiber:	Preis
Fachkräfte:	Nicht weiter in der Pflege benötigt
Patient*innen:	Pflege und Heilung
Pflegende:	Technologie: AI, Roboter, IoT
Gesellschaft:	Entmenschlichung
Gewinne:	Einfach skalierbare Versorgung
Risiken:	Technologiedominanz, Überwachung

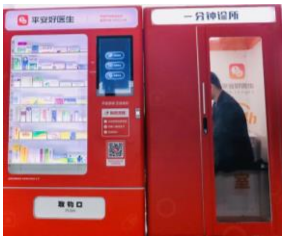

2. „Analog Care“

Hauptmerkmal:	Technologie verdammen
Treiber:	Furcht
Fachkräfte:	Überarbeitet
Patient*innen:	(statusabhängig) Unterversorgt
Pflegende:	Menschen
Gesellschaft:	Rückwärtsgerichtet
Gewinne:	Vertrautheit
Risiken:	Abgehängt werden

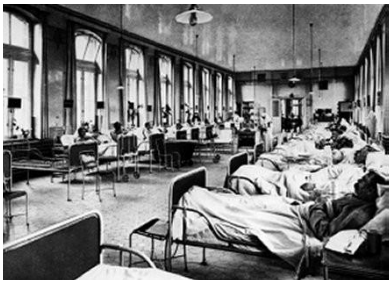

3. „Smart Care“

Hauptmerkmal:	Menschliche Pflege unterstützen
Treiber:	Technologie
Fachkräfte:	Überfordert
Patient*innen:	Pflege und Prävention
Pflegende:	Menschen
Gesellschaft:	Entsolidarisierung
Gewinne:	High-Tech Versorgung
Risiken:	Zweiklassen-Versorgung

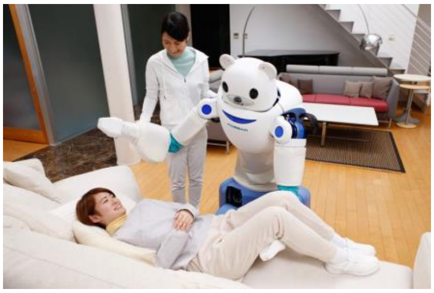

4. „Deep Care“

Hauptmerkmal:	Menschliche Pflege verstärken
Treiber:	Werte (Anspruch und Würde)
Fachkräfte:	Gestärkt
Patient*innen:	Gestärkt
Pflegende:	Teams
Gesellschaft:	Zukunftsgerichtet
Gewinne:	Menschlichkeit und High-Tech
Risiken:	Digital Divide

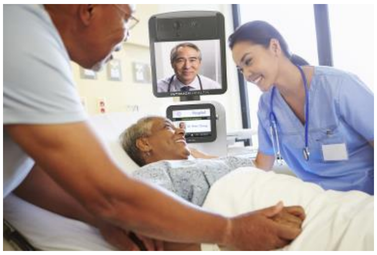

Vier Szenarien

	Robotic Care	Analog Care	Smart Care	Deep Care
Merkmal	Menschen ersetzen	Technologie verdammen	Menschen unterstützen	Menschliche Pflege stärken
Treiber	Preis	Furcht	Technologie	Werte
Fachkräfte	überflüssig	überarbeitet	überfordert	gestärkt
Patient*innen	Heilung	unterversorgt	Prävention	gestärkt
Pflegende	Technologie	Menschen	Menschen	Teams
Gesellschaft	entmenschlicht	rückwärts-gerichtet	entsolidarisiert	Zukunfts-gerichtet
Gewinne	Skalierbare Kosten	Vertrautheit	HighTech-Versorgung	Menschlichkeit und HighTech
Risiken	Überwachung	Abgehängt werden	Zweiklassen-Versorgung	Digitale Divide

Herausforderungen

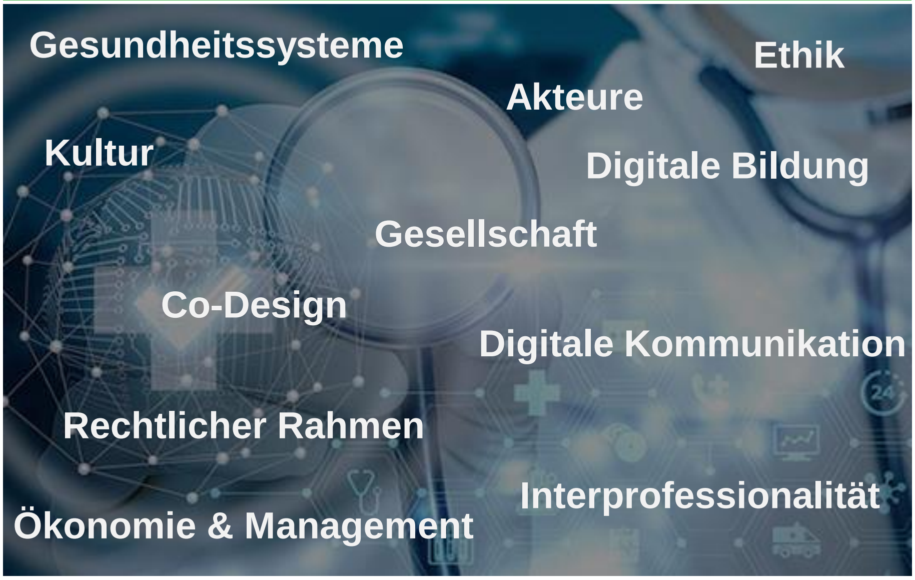

Gruppenarbeit

Dialogrunde 1: Pflegeszenarien 2025

Dialogrunde 2: Entwicklung eines bevorzugten Szenarios

Dialogrunde 3: Welche Schritte braucht es zur Umsetzung?

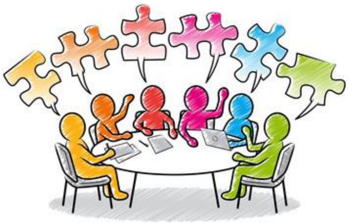

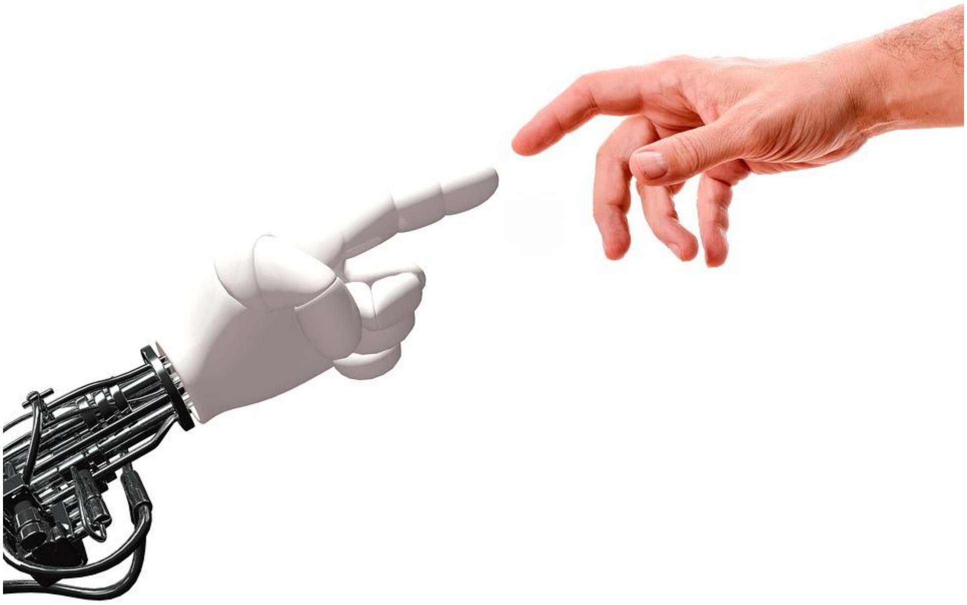

@medizindidaktik
zweikörperproblem
@medizindidaktik
Didaktik und Bildungsforschung
@JanP_Ehlers
@medizindidaktik

Emotionen und AI –
wie Maschinen lernen,
menschliche Emotionen zu deuten

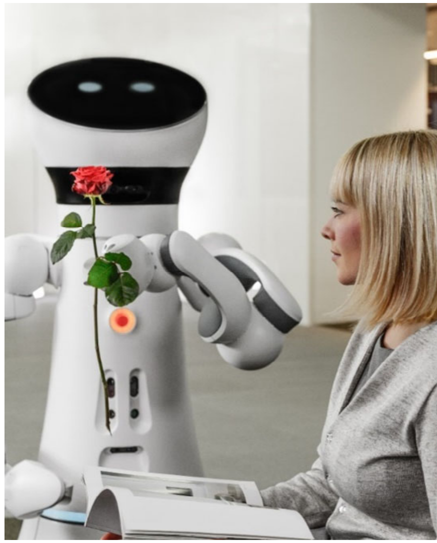

Wie werden Maschinen mit Schwerpunkt auf sozialer Interaktion entwickelt?
Wie gestaltet sich die Zusammenarbeit zwischen Entwicklern, Psychologen und Nutzern?

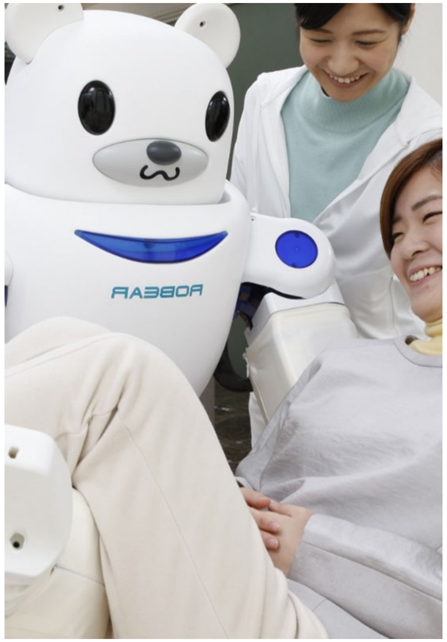

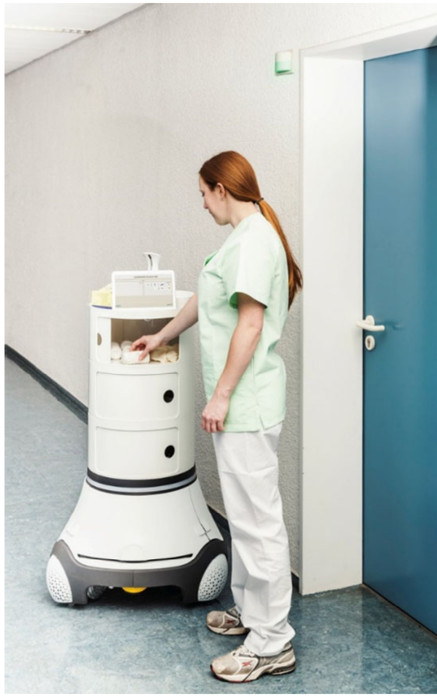

Oliver Korn, Hochschule Offenburg, 13.02.2020

Careum Dialog 2020. Zürich, 13.02.-14.02.2020
Talk to me. Soziale Roboter im Gesundheitswesen

Affective & Cognitive Institute
Hochschule Offenburg
offenburg.university

Soziale Roboter und Künstliche Intelligenz: Wo liegen die Potenziale?

- Kinder und die meisten Erwachsenen übertreffen sich darin, sozialen Robotern kognitive und emotionale Fähigkeiten zuzuschreiben, die diese eigentlich nicht haben oder demonstrieren.

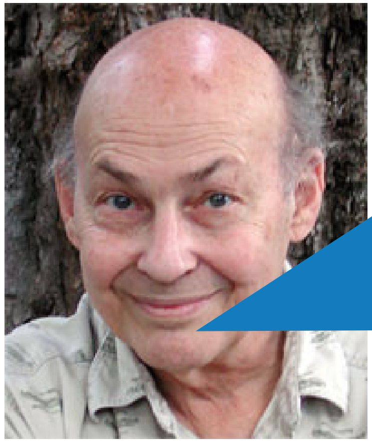

In from three to eight years we will have a machine with the general intelligence of an average human being. I mean a machine that will be able to read Shakespeare […] tell a joke, have a fight.

Marvin Minsky
Time Magazine (1970)

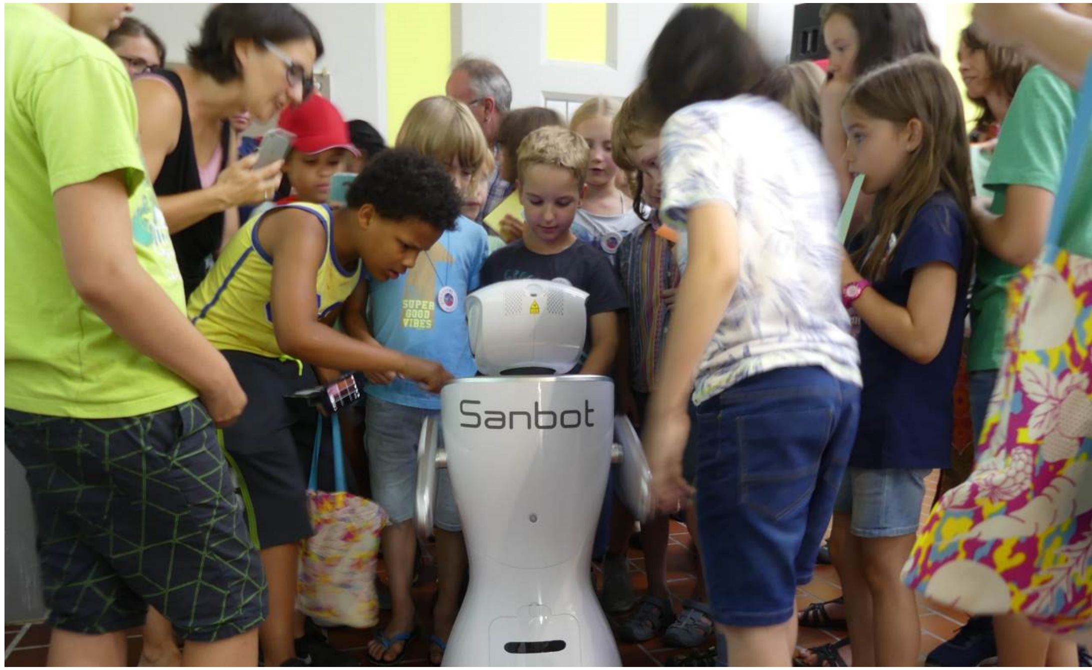

Unser Robot Sanbot interagiert mit Kindern am Hochschultag der Hochschule Offenburg.

Wann sind Roboter „sozial"?

- **Industrieroboter** und auch **kollaborative Roboter** sind für eingeschränkte Aufgaben konzipiert – der Fokus liegt auf der Arbeit, nicht dem Menschen.
- Im Gegensatz dazu werden **Serviceroboter** definiert als Roboter, die „für den Menschen nützliche Aufgaben ausführen, die physische Aufgaben unterstützen, wie z.B. Menschen zu helfen, sich zu bewegen" (ISO 8373:2012).
- **Soziale Roboter** sind zudem dafür ausgelegt, mit Personen zu **kommunizieren**.
- Sie interagieren und kommunizieren mit Menschen (oder anderen Robotern) und befolgen hierbei **soziale Verhaltensweisen und Regeln**.
- Erfolgreiche Kommunikation setzt ein Mindestmaß an **Empathie** (Einfühlungsvermögen) voraus.

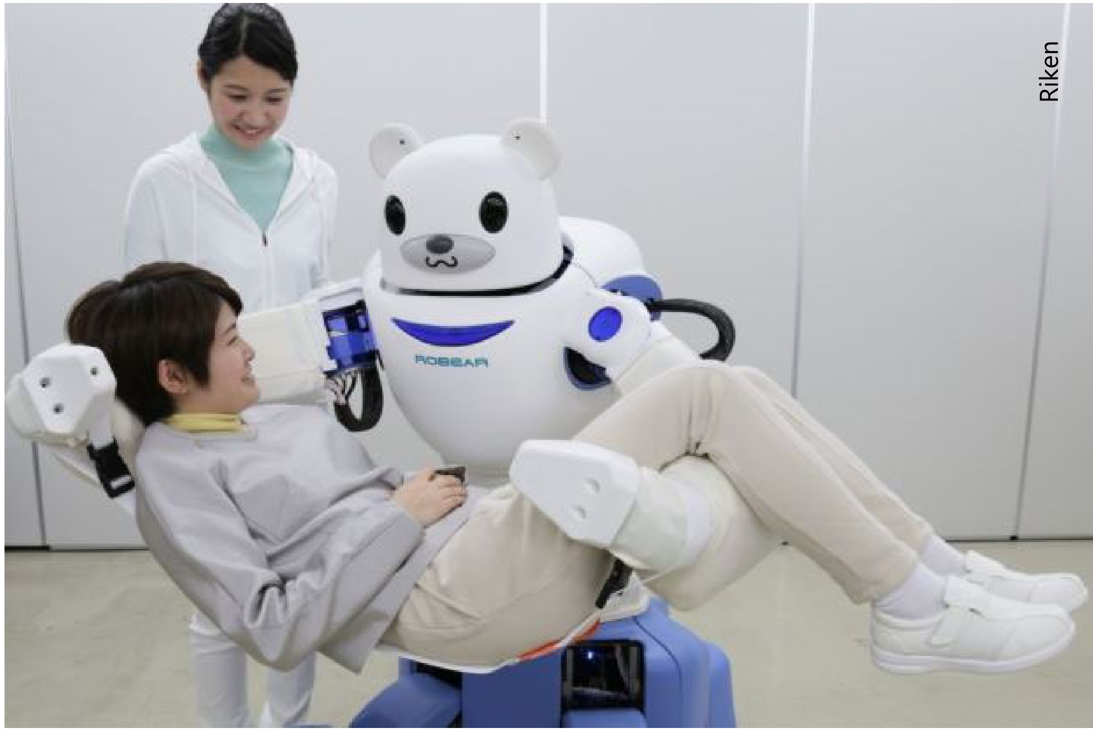

Riken

Der Serviceroboter „RoBear" von Riken war stark genug, um einen Patienten zu tragen – er hatte aber keinerlei Fähigkeiten zur Emotionserkennung.

Gibt es mehr Industrieroboter oder mehr Service-Roboter?

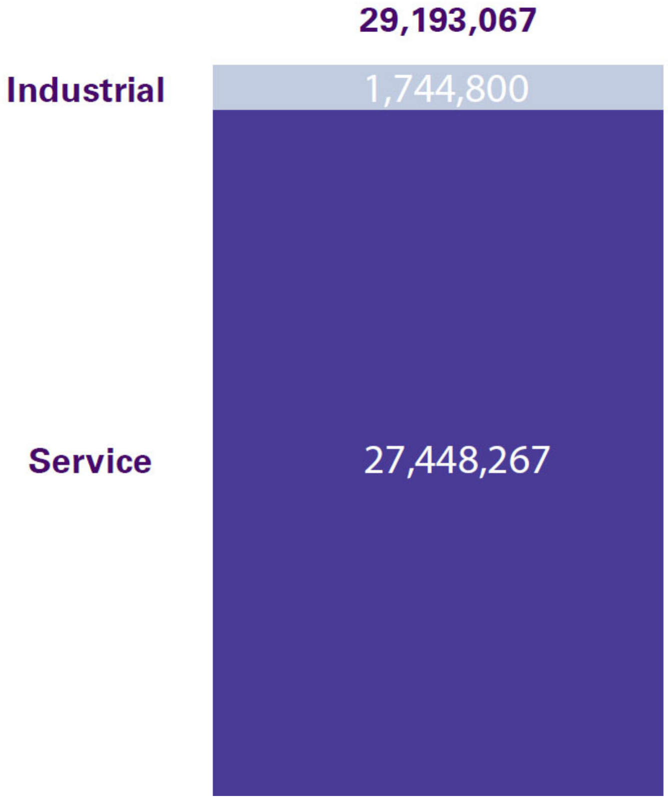

2016 KPMG Advisory N.V.

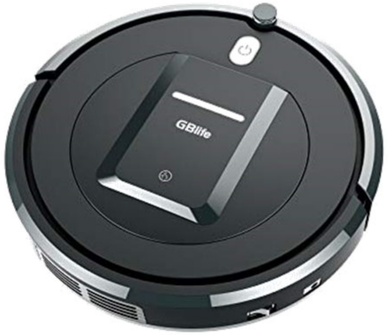

Technavio.com

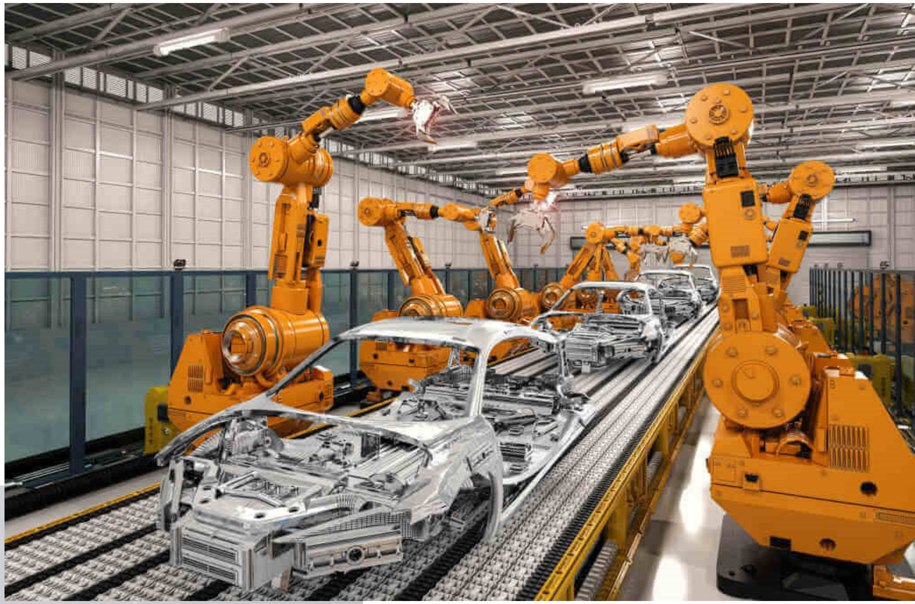

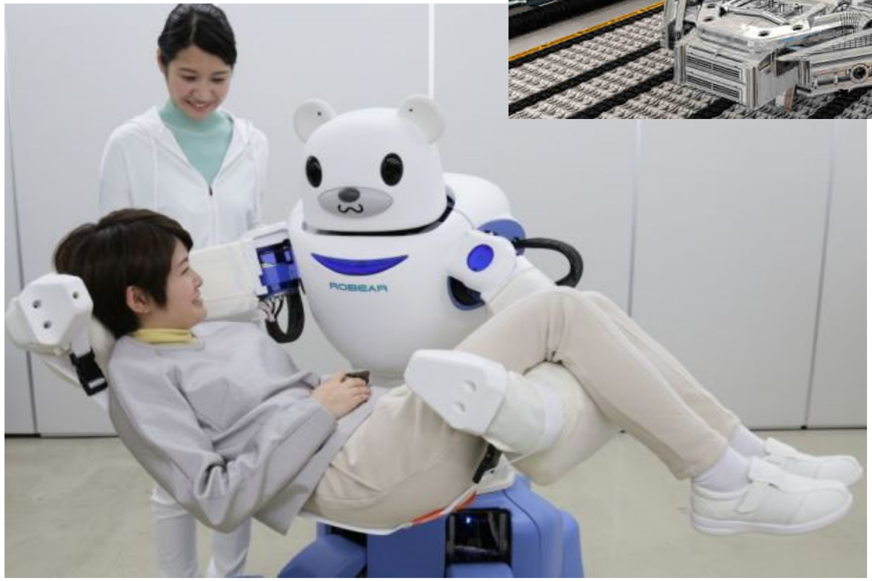

Riken

Wieviel Prozent der persönlichen Service-Roboter sind „sozial“?

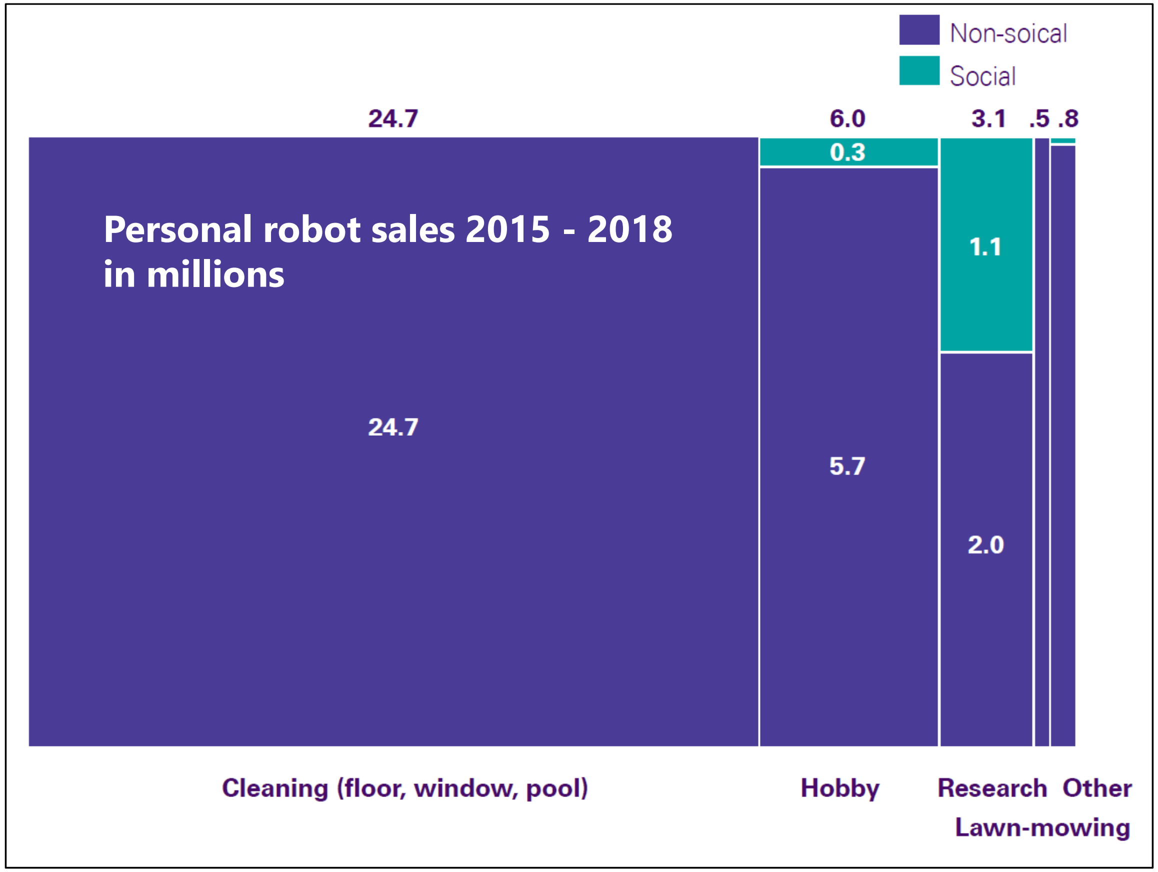

2016 KPMG Advisory N.V.

1,4 Mio. von 35,1 Mio.
= ca. 4%

Auswahl aktuell verfügbarer „sozialer“ Roboter

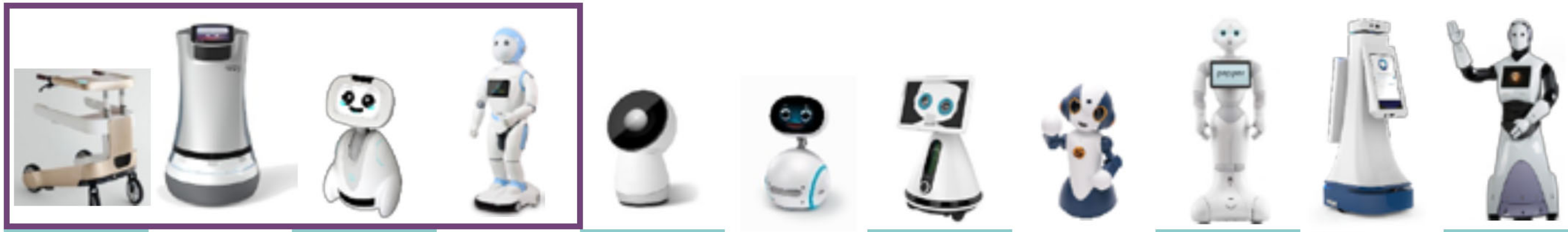

Name	LEA	Relay	Buddy	iPal	Jibo	Zenbo	Furo-i	Sota	Pepper	Oshbot	REEM
**Purpose**	LEA helps people to lead an active life, helps with daily routines, ensures safety and ease of communication	A robot that relays products from one person to another	A companion robot that protects the home, offers assistance, entertains and interfaces with smart home solutions	A friendly companion for children to play with and talk to in a natural manner	Helps adults to manage their life, helps seniors to live with greater independence and is a playmate for children	Your smart little companion	Create closer family relationships. A connection to home devices and a companion for the family	A "social dialogue" robot	Pepper is the first emotional robot. He was designed to be a true companion for daily life	Your autonomous retail robot assistant. Empowering your retail workforce with Inventory Management and Customer Service	A friendly full-size humanoid robot that will make your event unique
**Target market**	Elderly Care	Hospitality industry	Families	Children aged 3-8 years	Families	Families	Families	Elderly care	Both homes and businesses	Retail	Entertainment at events, conferences and fairs
**Company**	Robot Care Systems	Savioke	Blue Frog Robotics	Avatarmind	Jibo	Asus	Future Robot	Vstone	Aldebaran	Fellow Robots	PAL Robotics
**Headquarters**	The Netherlands	USA	France	USA	USA	Taiwan	Korea	Japan	France	USA	Spain
**Website**	robotcaresystems.com	savioke.com	bluefrogrobotics.com	avatarmind.com	Jibo.com	zenbo.asus.com	myfuro.com	vstone.co.jp	aldebaran.com	fellowrobots.com	pal-robotics.com

2016 KPMG Advisory N.V.

Aber: Was wäre, wenn Alexa sich bewegen könnte?

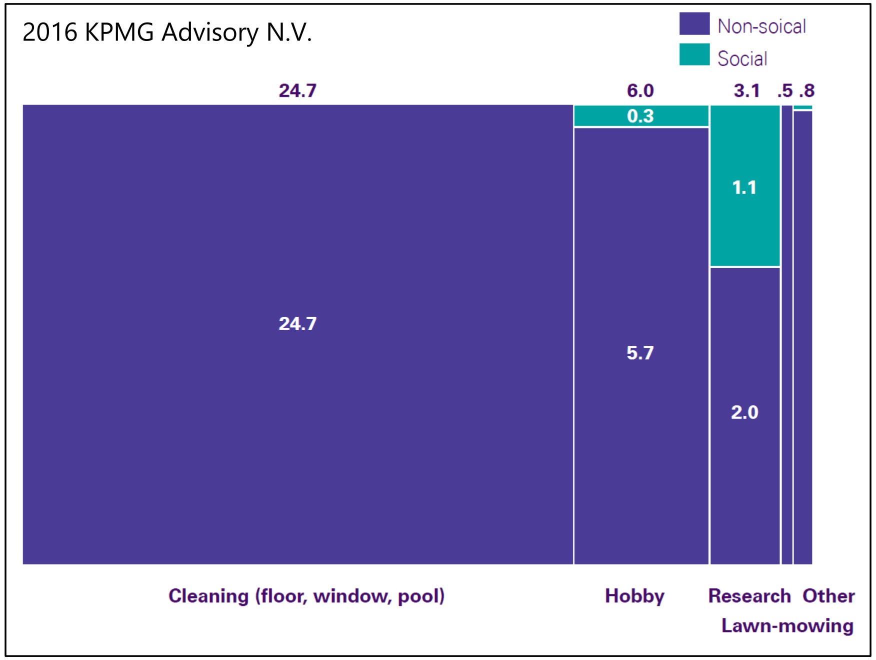

1,4 Mio. „sozial"
von 35,1 Mio.
= ca. 4%

220 Mio. „intelligente"
Assistenzgeräte 2020.

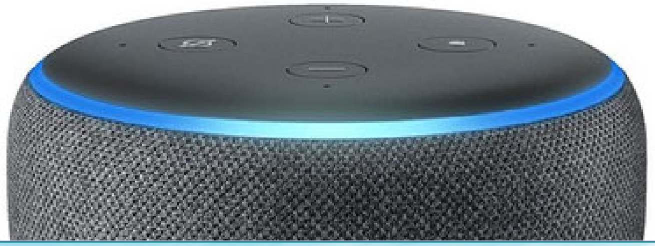

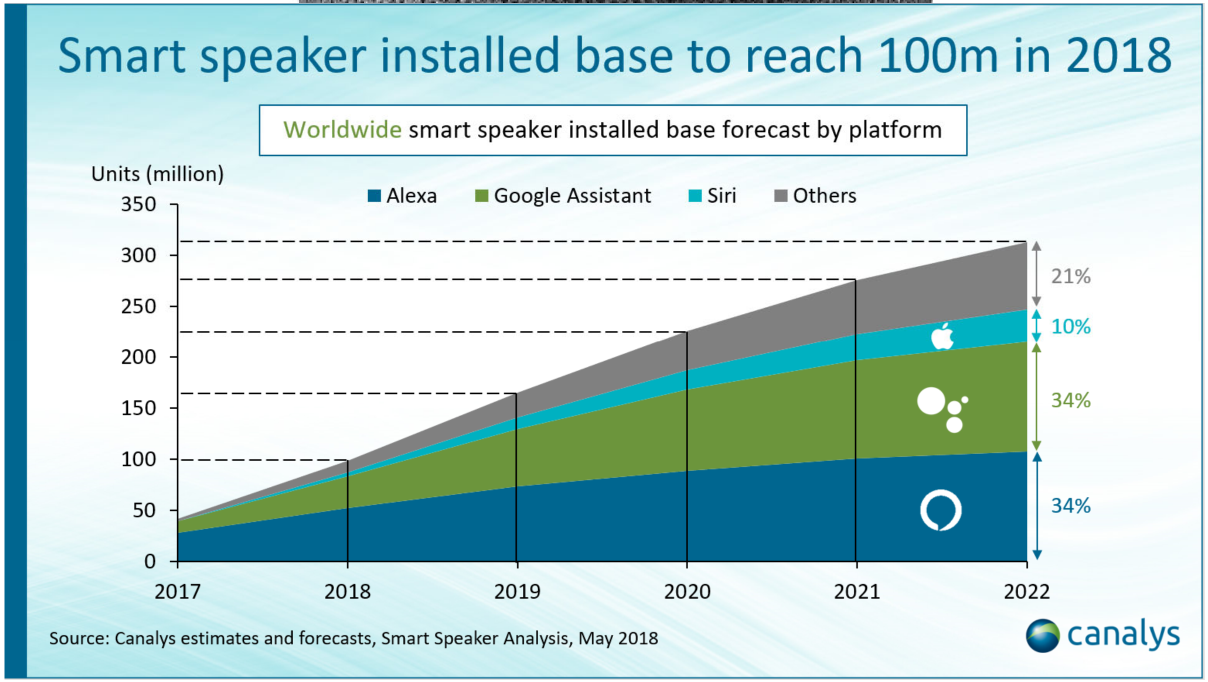

https://musically.com/2018/08/06/smart-speaker-sales-forecasts-market-share-amazon-google-apple/

Affective & Cognitive Institute
Hochschule Offenburg
offenburg.university

Soziale Roboter: Wie lernen Maschinen, Emotionen zu deuten?

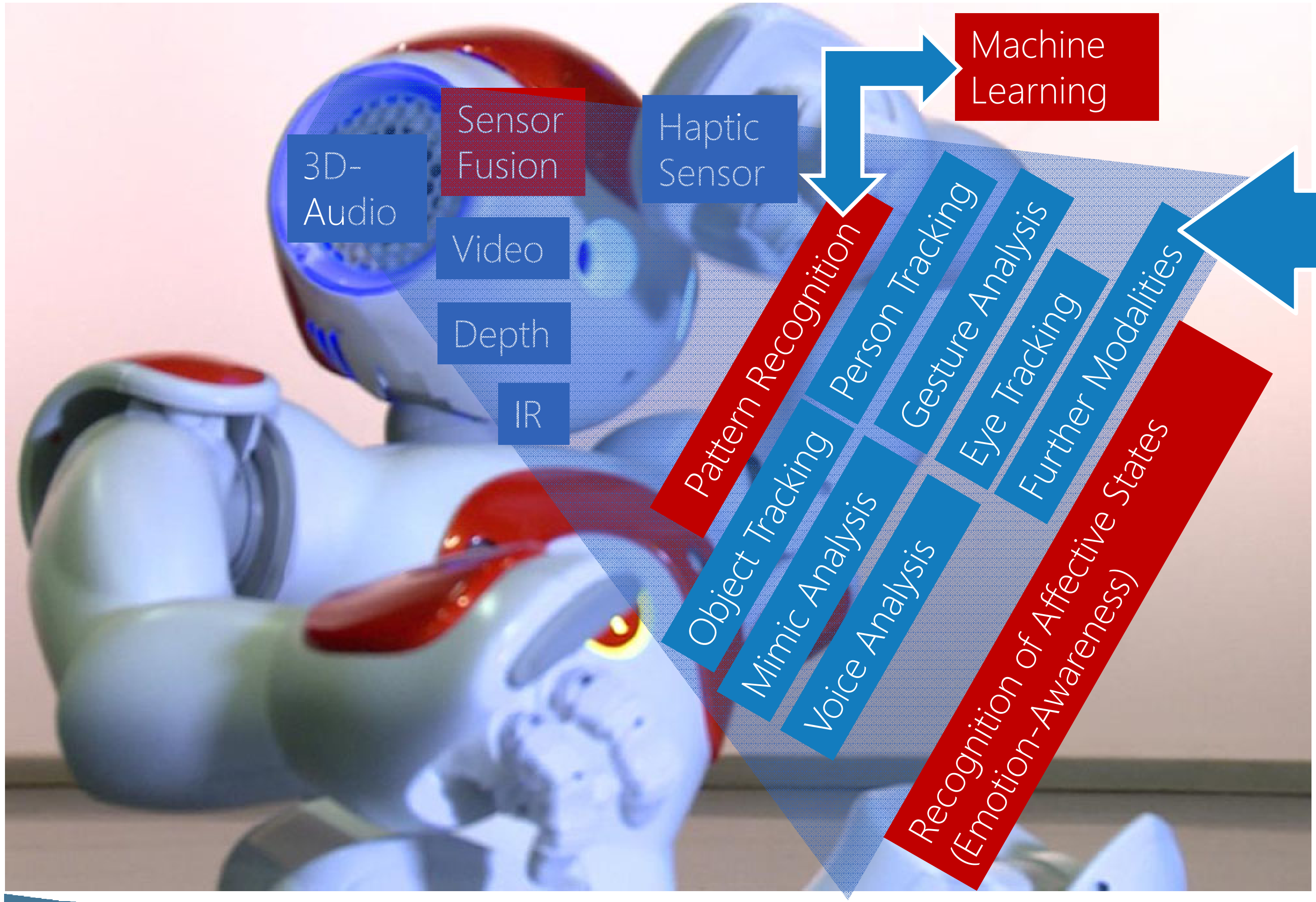

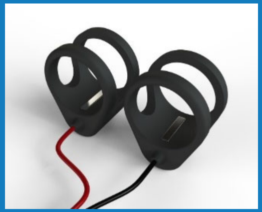

Electrodermal Activity

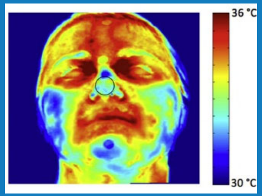

Thermal Regions in the Face

and several others…

Emotionserkennung: Beispiel Facial Tracking und Eye-Tracking

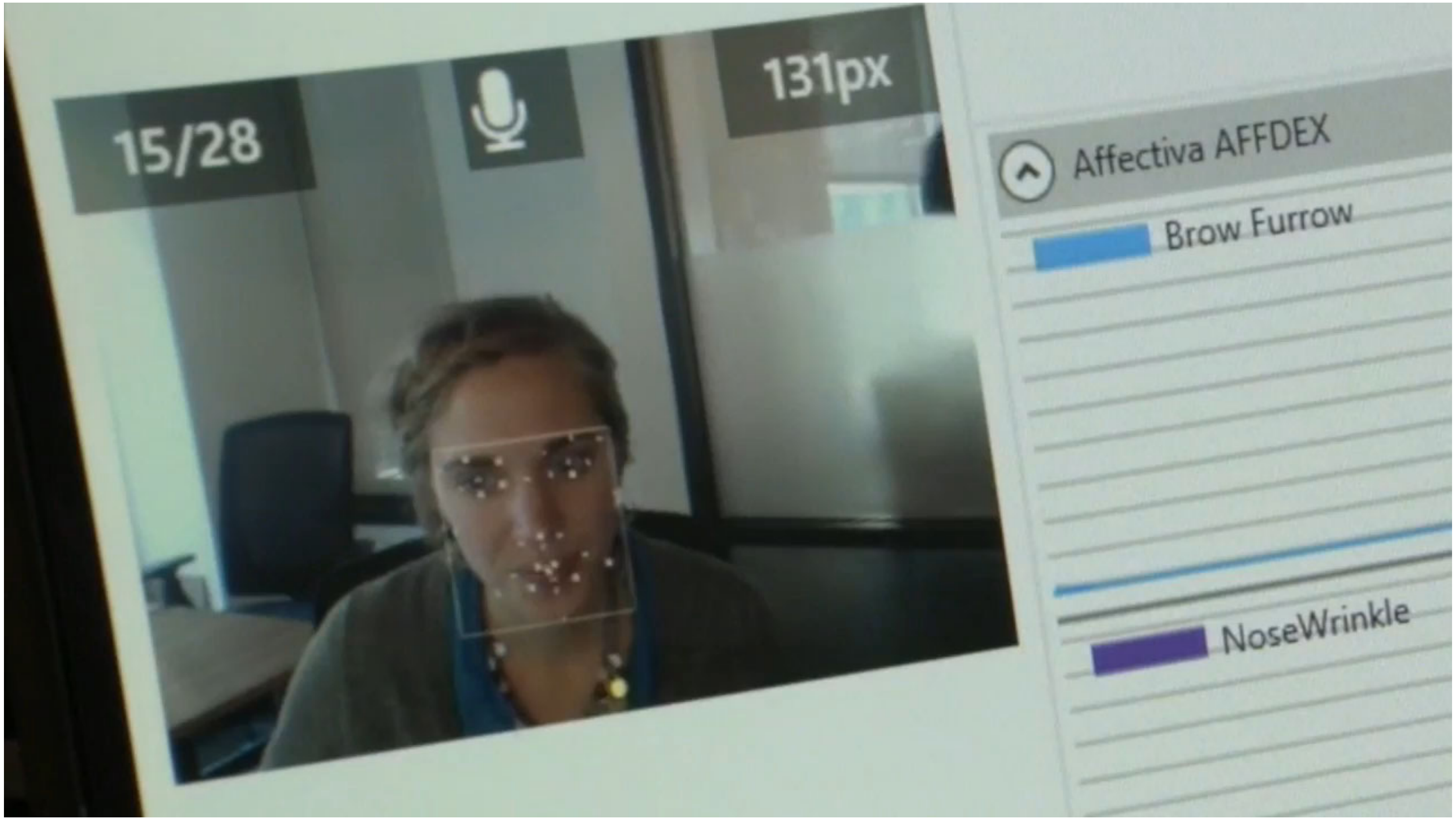

https://www.youtube.com/watch?v=136aVnTwpKU

iMotions

Zusammenarbeit zwischen Entwicklern, Psychologen und Nutzern

- **Entwickler:**
  Lieben die Technik – aber kennen die **Restriktionen**, z. B. die niedrige Auflösung vieler robotischer Kameras.
- **Psychologen und Kognitionswissenschaftler:**
  Kennen die **neuronalen Grundlagen.** Haben oft Probleme damit, dass Systeme auf Basis von Machine Learning zwar korrekte Interpretationen liefern, aber diese nicht „erklären" können, da sie **kein grundlegendes Verständnis** besitzen.
- **Nutzer:**
  Sind erstaunlich **gerne bereit**, an Experimenten teilzunehmen – und schreiben Systemen häufig **mehr kognitive und emotionale Fähigkeiten** zu, als diese derzeit besitzen.

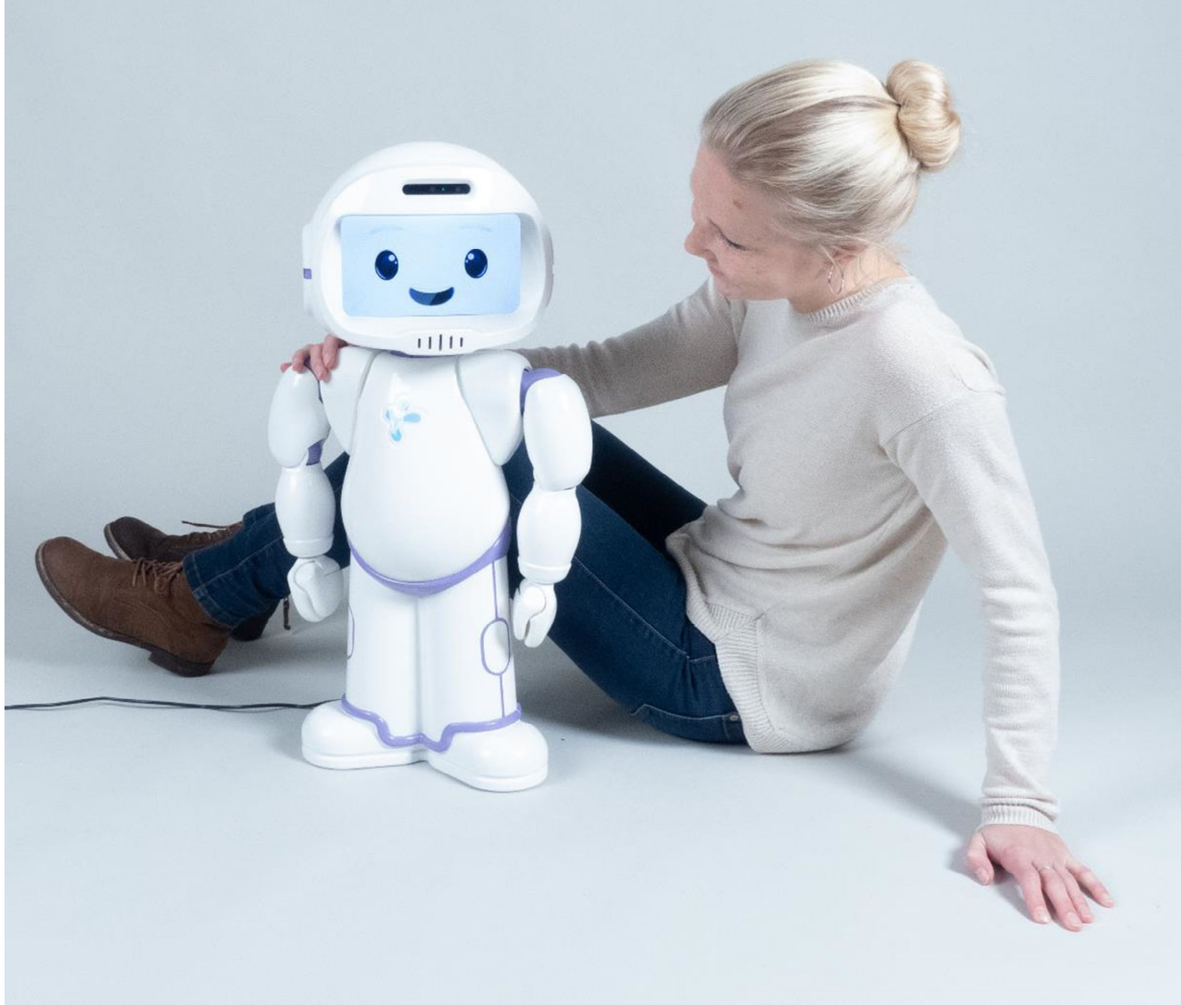

Beispiel: Studie zu Telepräsenz-Robotern in der Lehre

- **Anwender:**
  Studierende an der Hochschule Offenburg.
- **Ablauf:**
  Lehre wird einmal klassisch, einmal mit „Padbot“ und einmal mit einem „sozialem Roboter“ (hier: Sanbot) durchgeführt. Hierbei werden die Roboter direkt durch den Dozenten aus der Distanz gesteuert, sie sind NICHT autonom.
- **Ergebnis:**
  Technische Hürden trüben die Lehrerfahrung: Größe des Displays für Interaktionen, Stabilität der Datenverbindung (im Hochschulnetz!), langsame Fahrgeschwindigkeit.
- **Fazit:**
  Derzeitige Systeme eignen sich höchstens für die Interaktion mit kleinen Gruppen (2 bis maximal 5 Personen). Der soziale Roboter genießt anfangs einen Sympathie-Bonus (Novelty-Effekt).

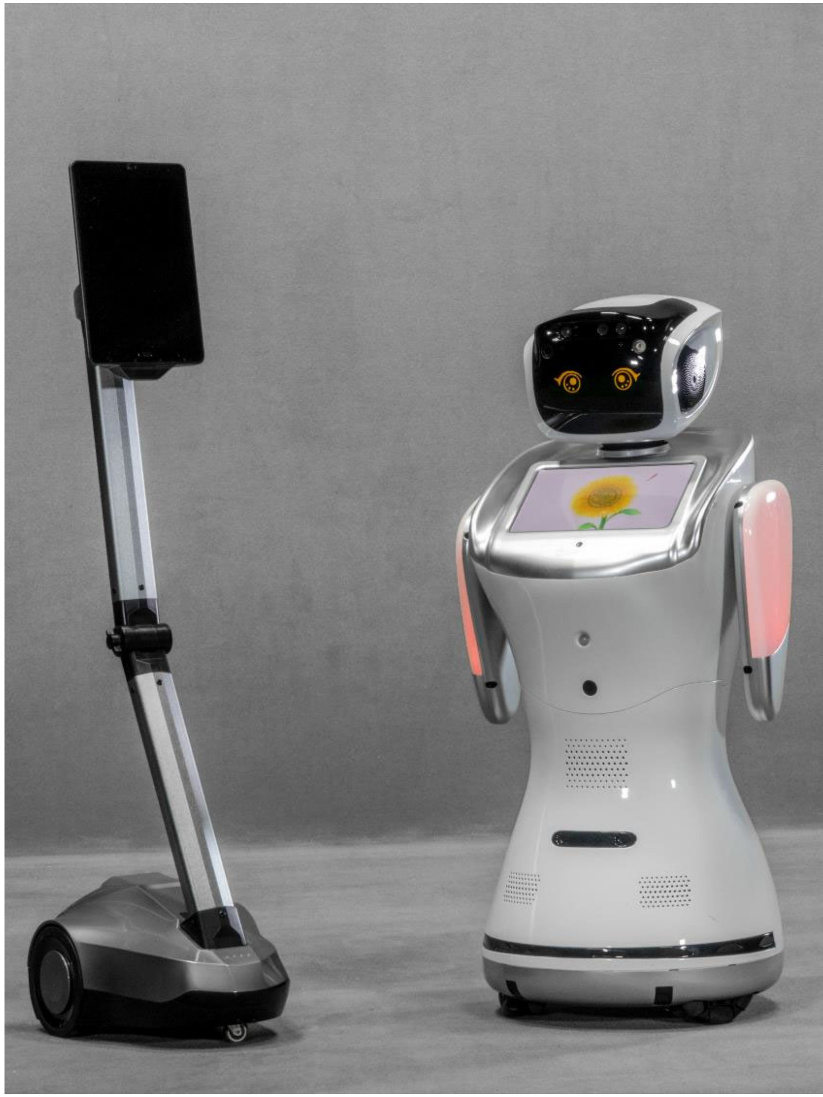

Soziale Roboter: Mögliche Weiterentwicklung

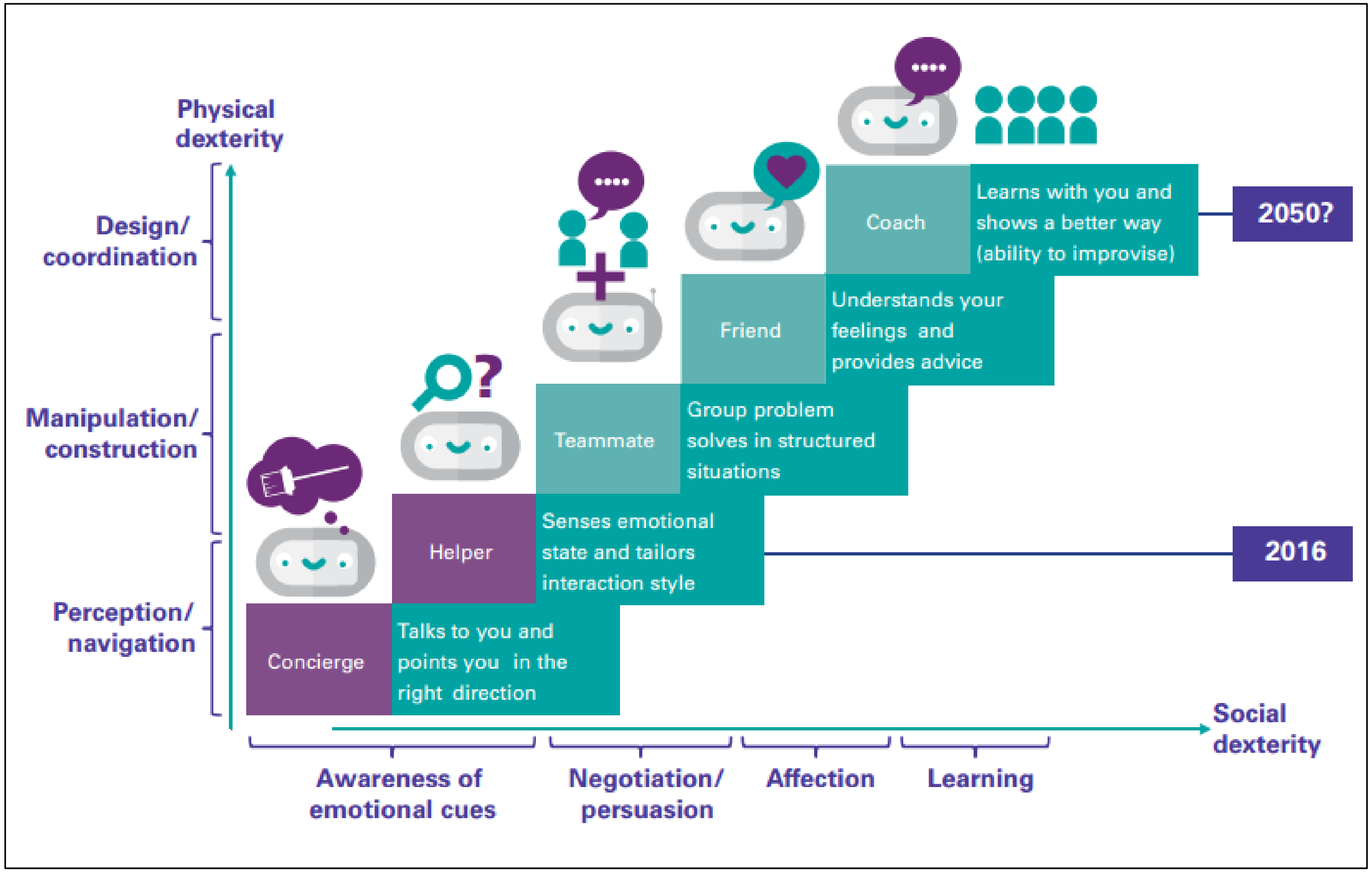

2016 KPMG Advisory N.V.

Nächste Schritte? Ein Buch und ein Wissenschafts-Comic

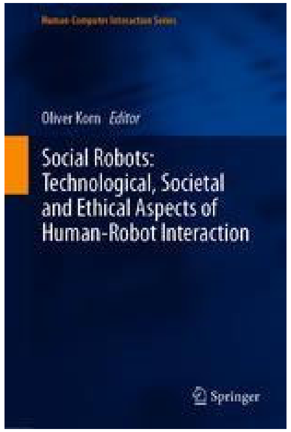

Korn, Oliver (Ed.) (2019): *Social Robots: Technological, Societal and Ethical Aspects of Human-Robot Interaction.*
Springer Berlin Heidelberg, ISBN 978-3-030-17106-3; 978-3-030-17107-0 (online).
https://doi.org/10.1007/978-3-030-17107-0

- Wissenschafts-Comic zu sozialen Robotern – mit vielen Hintergrundinformationen und Debatten zur ethischen Dimension.
- Plot: Ein sozialer Roboter wird gestohlen, und die junge Inspektorin Kira muss viel über diese Roboter lernen, um ihn zu finden.

https://affective-lab.org/comics/

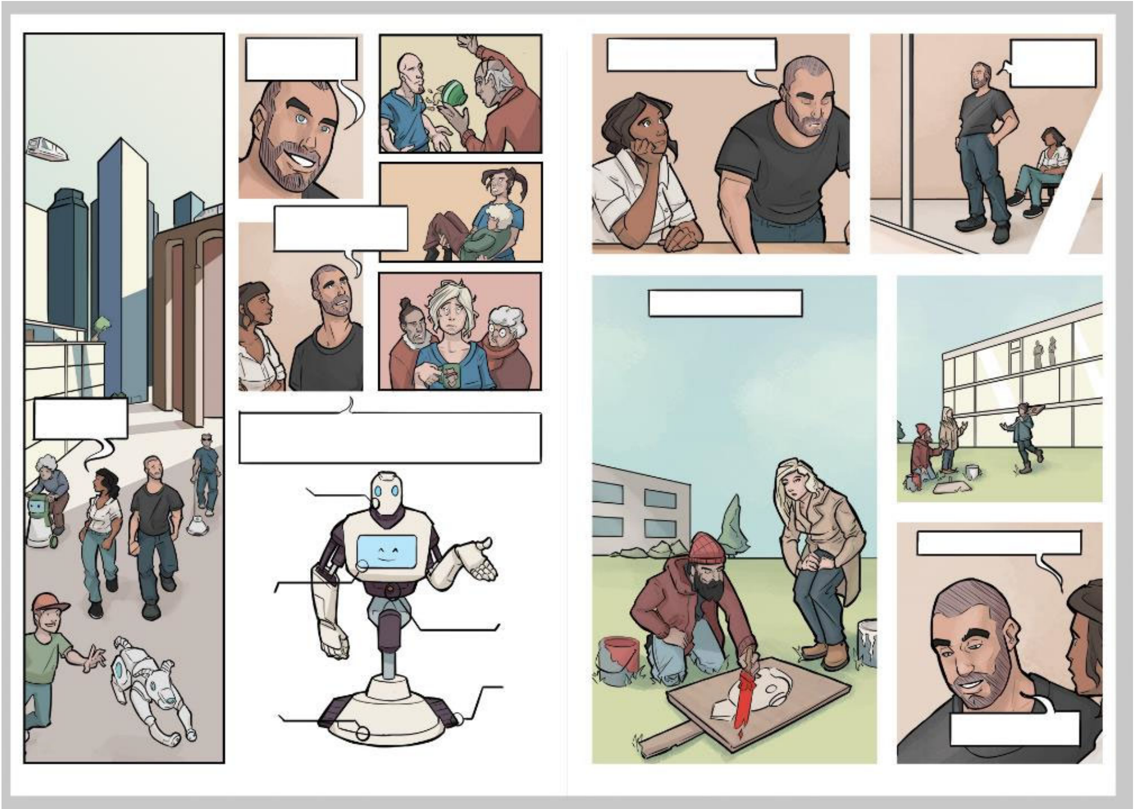

Einschätzung zur Zukunft sozialer Roboter

- Soziale Roboter sind **Publikumsmagnete**. Diese Eigenschaft machen sich auch **Scharlatane** zu Nutze, z. B. in der irreführenden medialen Darstellung des Roboters Sophia.
- Bei aktuell verfügbareren sozialen Robotern sind die **hardware-basierten Fähigkeiten noch sehr begrenzt**: Sie sind weder schnell noch agil und daher von überschaubarem praktischem Nutzen.
- Die Akzeptanz von **Assistenzsystemen** wie Alexa und zugleich **Staubsaugrobotern** zeigt aber, dass ein **großer Markt** für unterstützende Systeme existiert – und zudem viele Konsumenten nach wie vor bereit sind, Datenschutz hinten anzustellen.
- Die **technische Entwicklung von Emotionserkennung ist weit fortgeschritten** und ermöglicht grundsätzlich schon heute die Herstellung emotionssensitiver sozialer Roboter.
- Für die weitere Verbreitung **fehlt derzeit ein robustes System**, das zum Preis etwa eines Mittelklassewagens **einfache Hilfsfunktionen** wie Holen und Bringen übernehmen könnte.

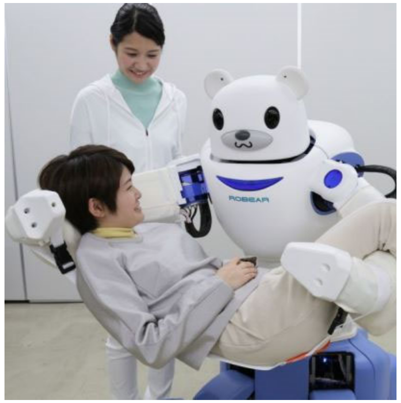

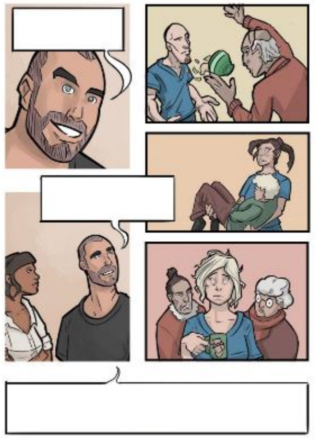

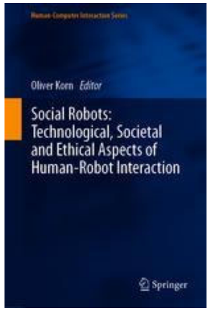

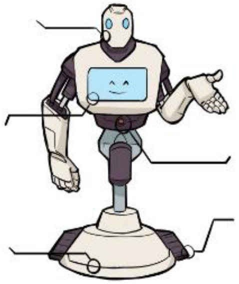

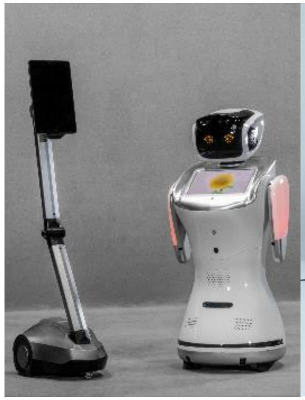

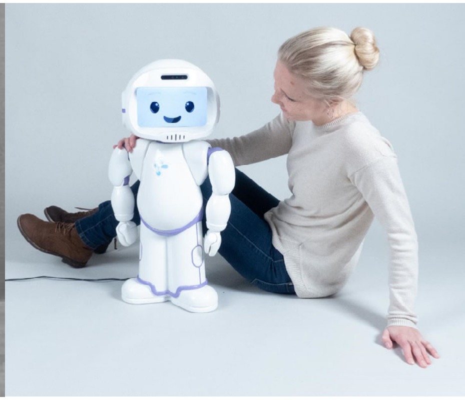

Danke!

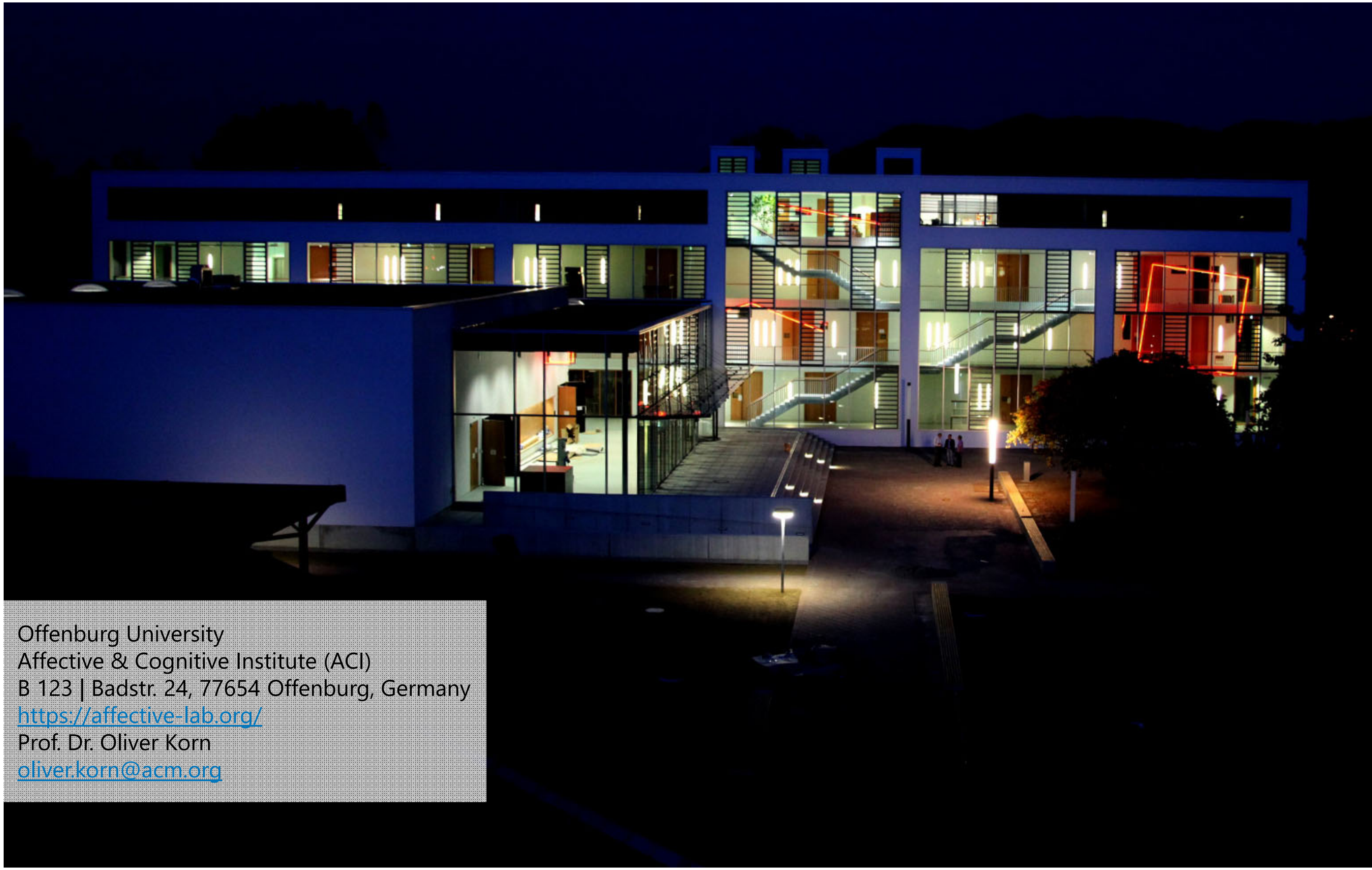

Offenburg University
Affective & Cognitive Institute (ACI)
B 123 | Badstr. 24, 77654 Offenburg, Germany
https://affective-lab.org/
Prof. Dr. Oliver Korn
oliver.korn@acm.org

Affective & Cognitive INSTITUTE
Hochschule Offenburg
offenburg.university

Fachhochschule Nordwestschweiz

Was macht Roboter sozial?

Prof. Dr. Oliver Bendel

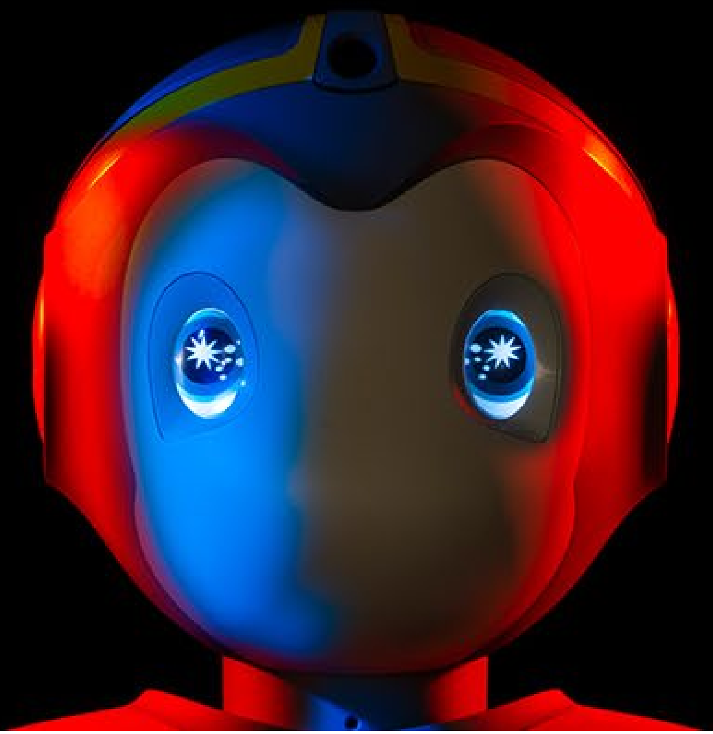

Quelle: Daimler und Benz Stiftung

Zur Person

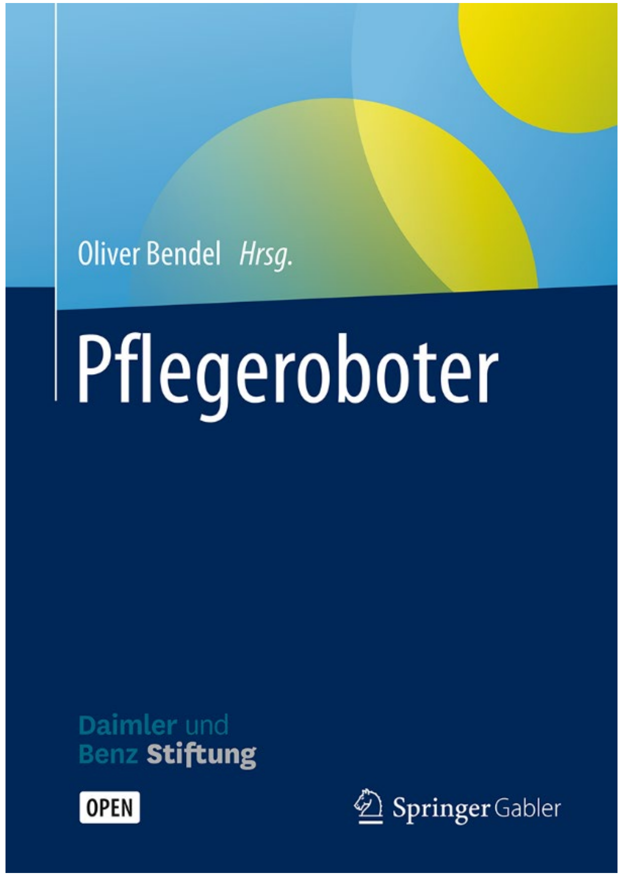

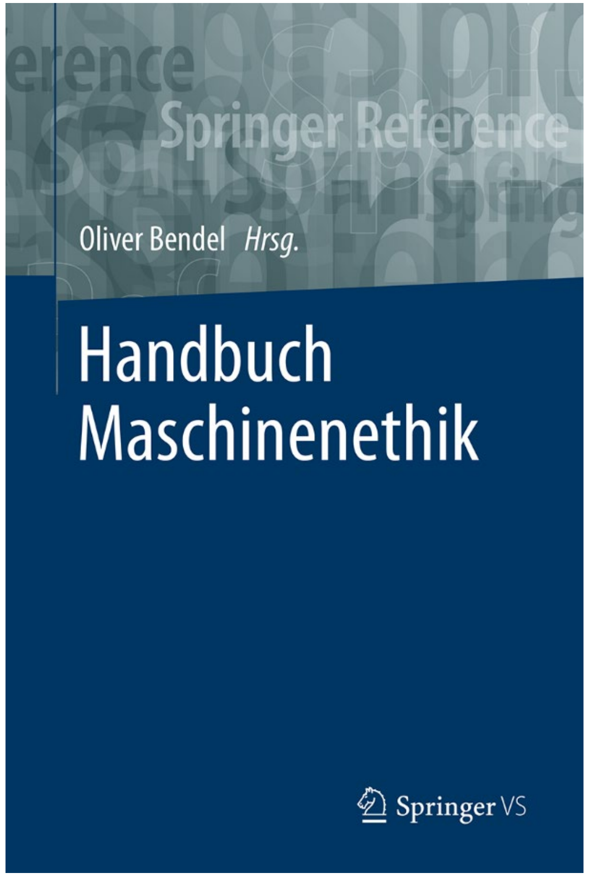

- Seit 2009 Professor für Wirtschafts-informatik und Ethik
- Seit den 1990er-Jahren Beschäftigung mit Informations- und Maschinenethik
- Aktuelle Bücher: «Pflegeroboter» (2018), «Handbuch Maschinenethik» (2019), «Maschinenliebe» (Ende 2020)
- Aktuelles Projekt: «Roboter, Empathie und Emotionen» (mit H. Schulze und M. Schubert, für TA-SWISS, 2019 – 2020)

Soziale Roboter

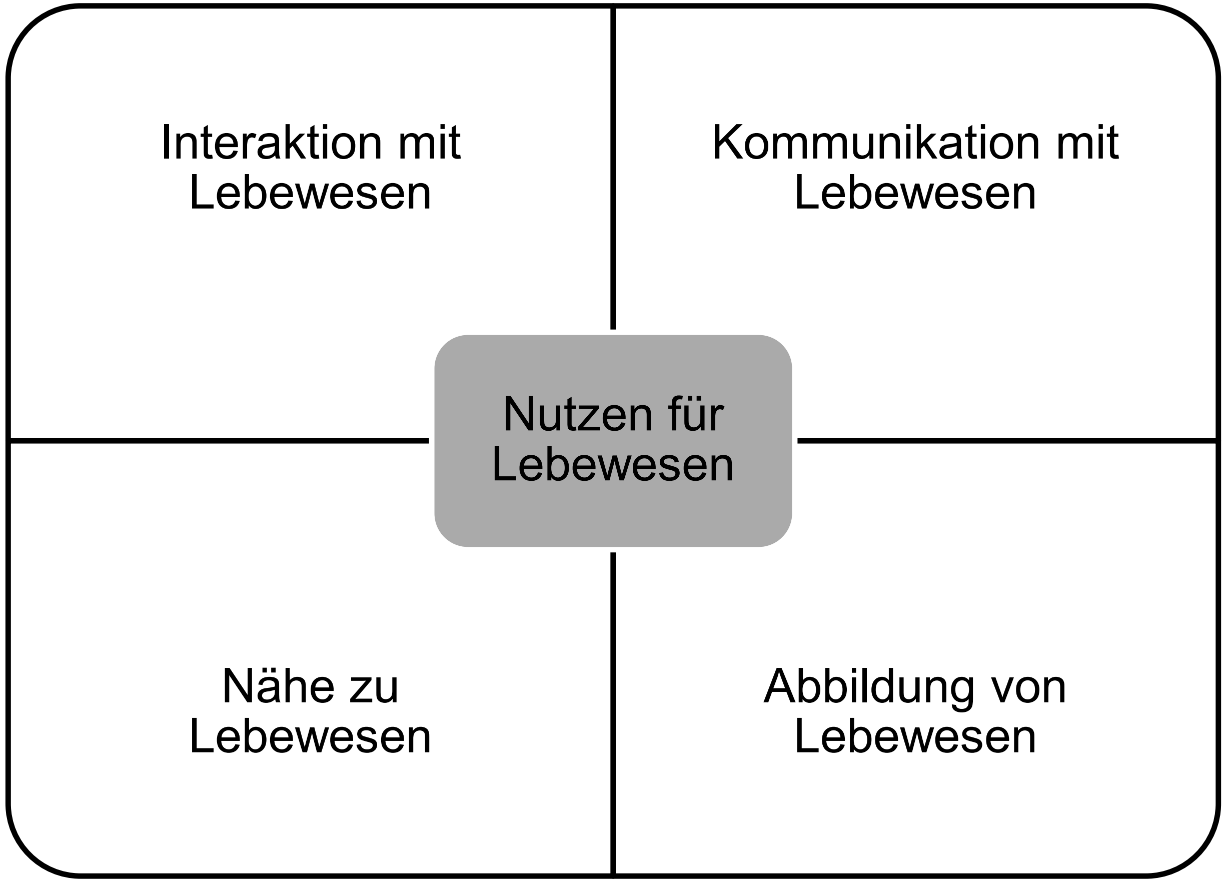

- Hardwareroboter (etwa Service- oder Spielzeugroboter) oder – bei einem weiten Begriff – Softwareroboter (etwa Chatbots, Voicebots, Social Bots)
- Manchmal dinghaft, oft tierähnlich (animaloid) oder menschenähnlich (humanoid) gestaltet
- Sozial im Sinne des Zusammenseins mit Menschen und Tieren oder der Unterstützung von Menschen und Tieren

Spielzeugroboter: Cozmo von Anki

Interaktion
Ist autonom oder steuerbar
Hat Gesichtserkennung
Spielt mit Benutzer
Stapelt Würfel
Ist programmierbar
Bewegt sich durch die Gegend

Kommunikation
Kann Gegenüber fixieren
Verändert Augenausdruck
Spricht natürliche Sprache
Nennt Namen des Benutzers
Zeigt Emotionen
Hat Kamera und Lautsprecher
Verbunden mit App

Nutzen
Unterhält und bespasst
Lehrt Programmierung

Nähe
Ist gedacht für Nähe zu Menschen
Kann Benutzer berühren
Kann Nähe zu Tieren haben
Teilt Zimmer des Benutzers
Benutzer soll Beziehung aufbauen

Abbildung
Ist abstrakt, animaloid, humanoid gestaltet
Hat beweglichen Kopf
Hat bewegliche Augen
Hat bewegliche Arme
Hat keine Beine, sondern Raupen

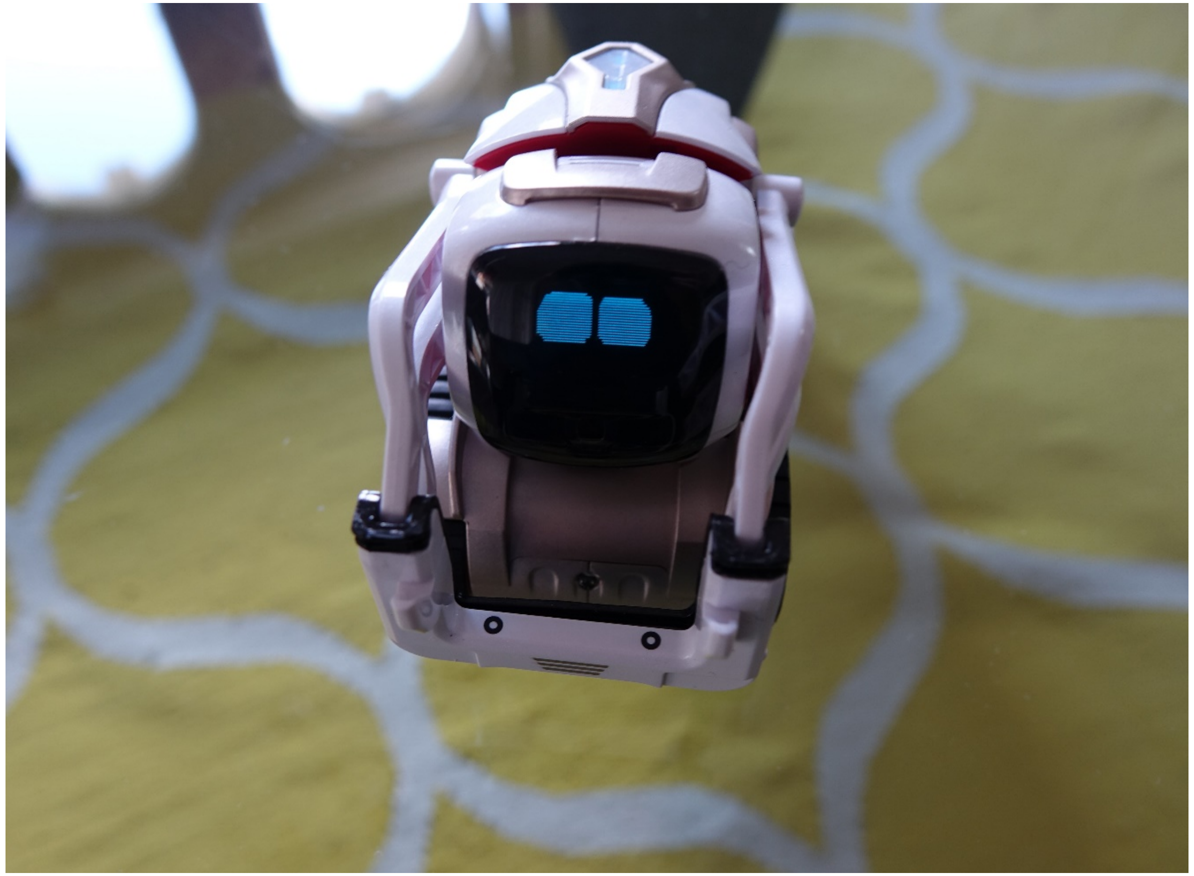

Sexroboter: Harmony von Realbotix

Interaktion
Ist autonom oder steuerbar
Hat Spracherkennung
Spielt mit Benutzer
Ist programmierbar
Ist penetrierbar
Verfügt über Avatar

Kommunikation
Kann Gegenüber fixieren
Verändert Augenausdruck
Spricht und versteht natürliche Sprache
Zeigt Emotionen
Nutzt separaten Touchscreen
Verbunden mit App
Hat Kameras und Mikrofone

Nutzen
Kann Partnerin sein
Unterhält und verschafft Lust

Nähe
Ist gedacht für Nähe zu Menschen
Kann zur Partnersexualität dienen
Besitzt Körperöffnungen, um mit Benutzern verschmelzen zu können
Teilt Bett und Zimmer des Benutzers
Benutzer soll Beziehung aufbauen

Abbildung
Ist humanoid gestaltet
Hat beweglichen Kopf
Hat bewegliche Augen und -lider
Hat beweglichen Mund
Hat bewegbaren Körper
Hat künstliche Intelligenz

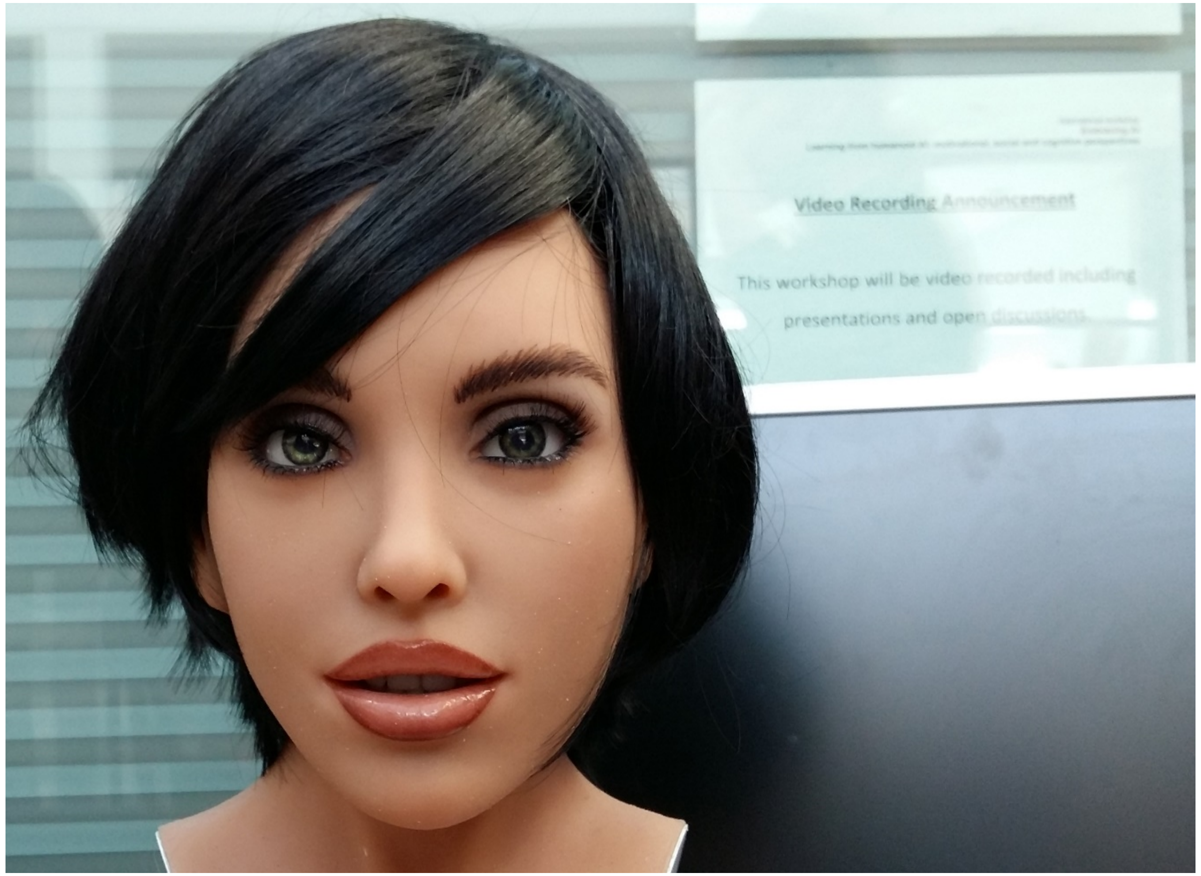

Pflegeroboter: P-Care von F&P Robotics/ZRFN Robotics

Interaktion
Ist autonom
Hat Gesichts- und Spracherkennung
Kann Gegenstände bringen, reichen und öffnen
Nutzt Cobot-Technologie
Bewegt sich durch die Gegend

Kommunikation
Kann Gegenüber fixieren
Verändert Augenausdruck
Spricht und versteht natürliche Sprache
Zeigt Emotionen
Nutzt integrierten Touchscreen
Hat Kameras und Mikrofone

Nutzen
Unterstützt Pflegekraft und -bedürftigen
Unterhält und informiert

Nähe
Ist gedacht für Nähe zu Menschen
Weicht Benutzer aus oder hält vor ihm an
Kann Benutzer umarmen
Kann Türen auf- und zumachen
Teilt Zimmer des Benutzers

Abbildung
Ist animaloid gestaltet
Hat beweglichen Kopf
Hat bewegliche Augen
Hat bewegliche Arme und Finger
Hat keine Beine, sondern Rollen
Hat künstliche Intelligenz

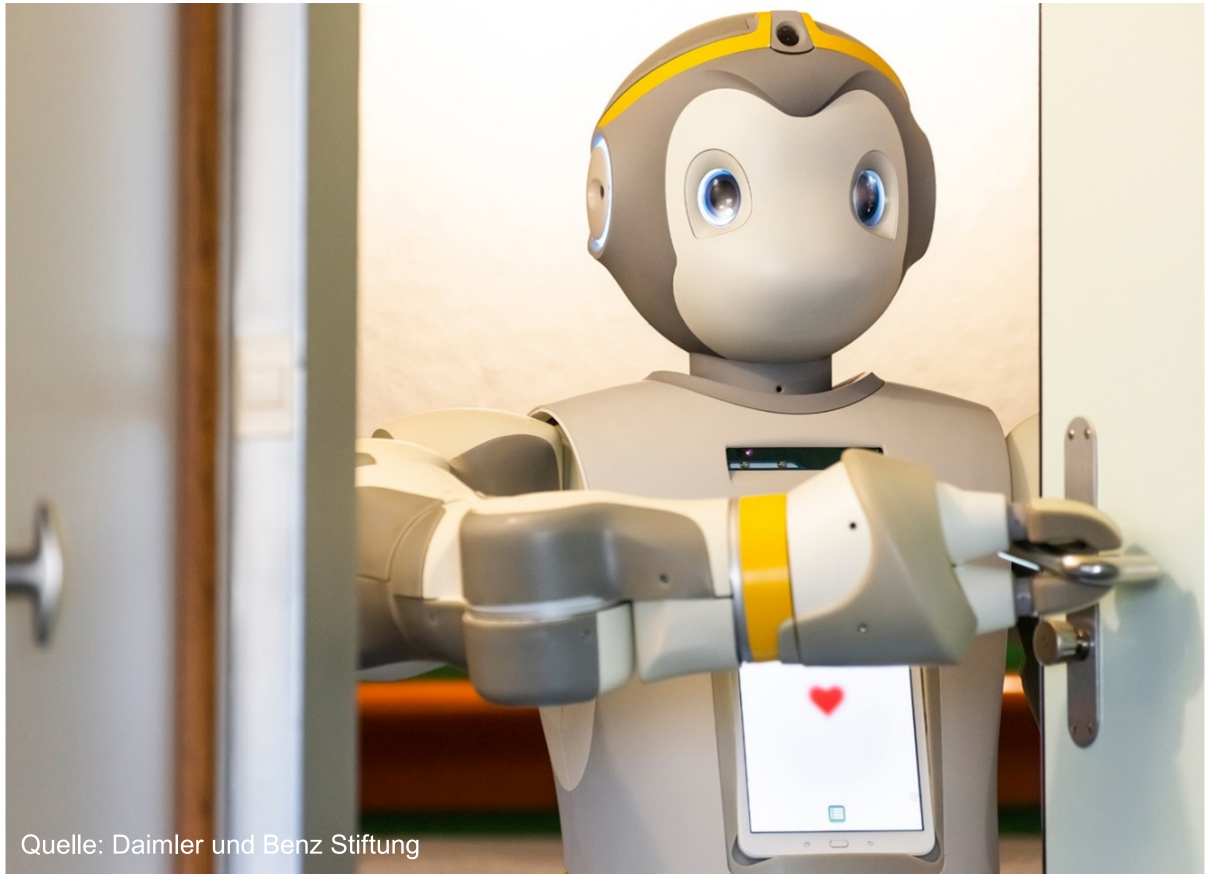
Quelle: Daimler und Benz Stiftung

Sicherheits- und Überwachungsroboter: Robocop/REEM von PAL Robotics

Interaktion
Ist autonom
Hat Gesichts-, Gestik- und Emotionserkennung
Zeigt Benutzern den Weg
Erlaubt Einzahlungen über Touchscreen
Filmt die Umgebung

Kommunikation
Wendet sich Benutzer zu
Spricht und versteht natürliche Sprache
Kann salutieren und grüßen
Nennt Namen des Benutzers
Nutzt integrierten Touchscreen
Hat Kameras und Mikrofone

Nutzen
Informiert und navigiert
Überwacht Umgebung

Nähe
Ist gedacht für Nähe zu Menschen
Weicht Benutzer aus oder hält vor ihm an
Begleitet den Benutzer
Teilt mit Benutzer den öffentlichen und halböffentlichen Raum

Abbildung
Ist humanoid gestaltet
Hat beweglichen Kopf
Hat Augen
Hat bewegliche Arme, Hände, Finger
Hat keine Beine, sondern Rollen
Trägt gleiche Mütze wie Beamte
Hat künstliche Intelligenz

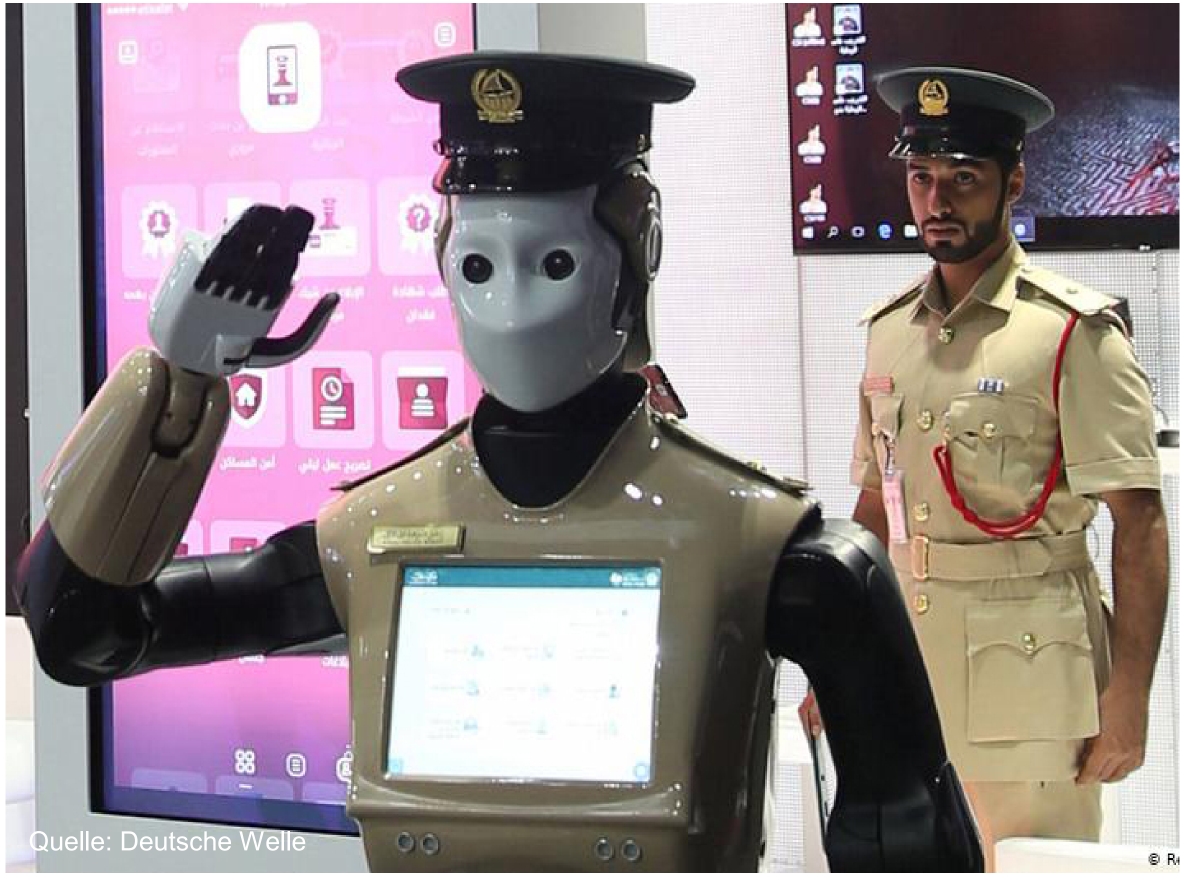

Quelle: Deutsche Welle

Haushalts- und Gartenroboter: Happy Hedgehog der HSW-FHNW

Interaktion
Ist autonom
Bewegt sich durch die Gegend
Erkennt Igel mit Wärmebildkamera und Bilderkennung
Unterbricht für Igel seine Arbeit
Folgt einer moralischen Regel

Kommunikation
Macht Geräusche beim Fahren
Hat im Betrieb ein Lichtsignal
Kann in einer Erweiterung den Benutzer informieren
Hat Kamera

Nutzen
Rettet Igel
Verschafft gutes Gewissen

Nähe
Ist gedacht für Nähe zu Tieren, insbesondere zu Igeln
Braucht keine Nähe zu Menschen

Abbildung
Ist abstrakt gestaltet
Hat keine Beine, sondern Rollen
Hat künstliche Intelligenz
Hat maschinelle Moral

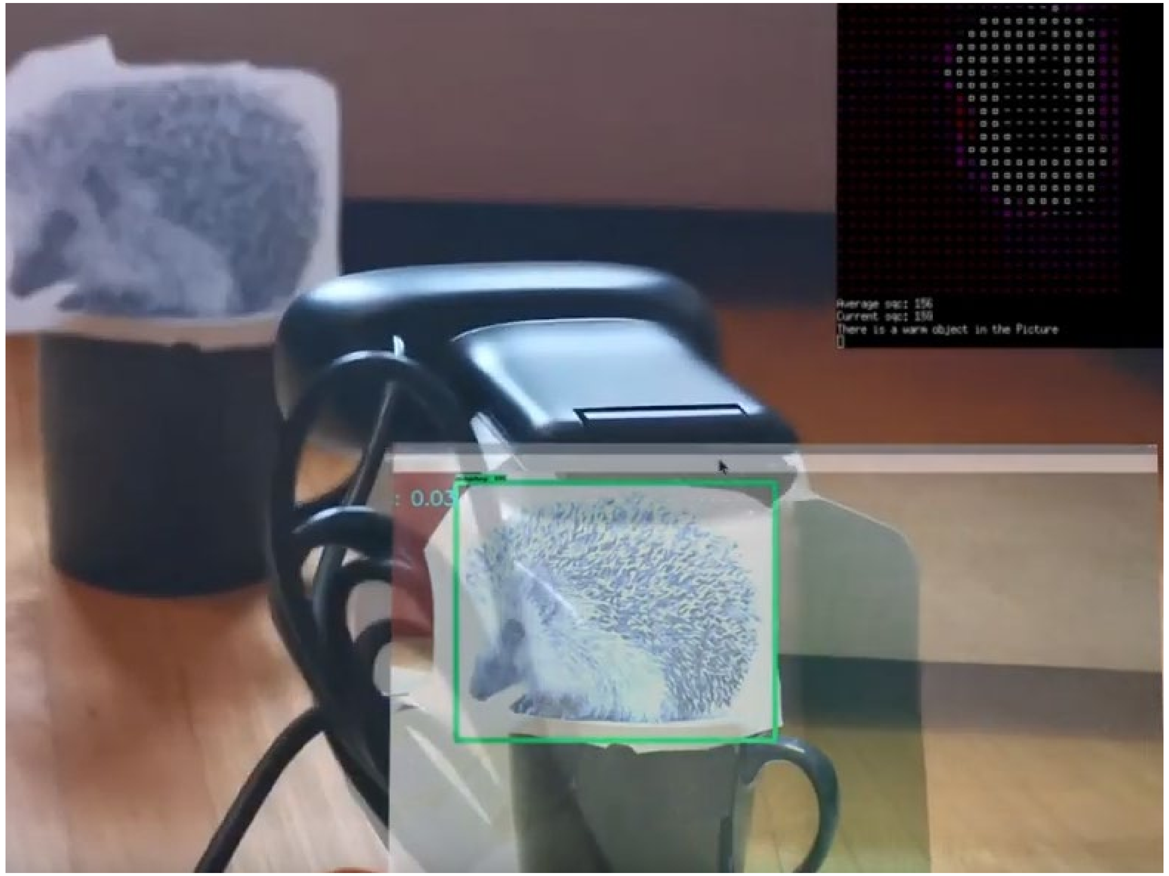

Zusammenfassung

Soziale Roboter gibt es in allen möglichen Anwendungsbereichen und mit unterschiedlichen Aufgaben.

Sie können soziale Funktionen mit Blick auf Menschen, aber auch mit Blick auf Tiere aufweisen.

Sie haben unterschiedliche Möglichkeiten und Ausprägungen in Bezug auf Interaktion, Kommunikation, Nähe und Abbildung.

Zusammenfassung

Ein paar Roboter können unsere Partner werden, wobei die Beziehungen einseitig bleiben.

Manche soziale Roboter zeigen Emotionen (die sie nicht haben), und wecken sie, was aus Sicht der Ethik problematisch sein kann.

Problematisch sind in moralischer Hinsicht auch die Möglichkeiten von Sprach- und Gesichtserkennung (Privat- und Intimsphäre).

Fachhochschule Nordwestschweiz

Kontakt

Prof. Dr. Oliver Bendel
Hochschule für Wirtschaft FHNW
Institut für Wirtschaftsinformatik
Bahnhofstrasse 6
CH-5210 Windisch

oliver.bendel@fhnw.ch

oliverbendel.net informationsethik.net
maschinenethik.net robophilosophy.com

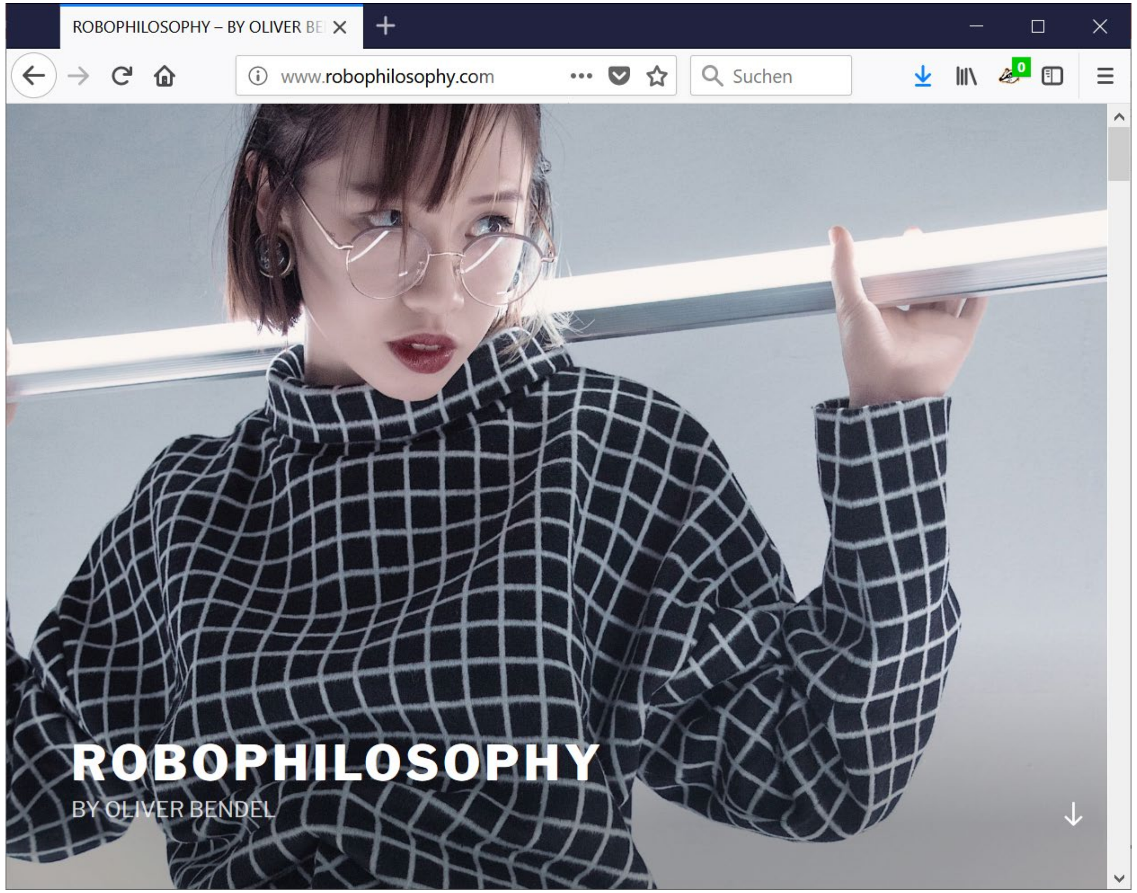

Supplement: Multimedia Appendix 2 [file jmir_v23i11e20046_app2.pdf]
